# The Computational and Neural Substrates of Ambiguity Avoidance in Anxiety

**DOI:** 10.5334/cpsy.67

**Published:** 2022-02-03

**Authors:** EMMA L. LAWRANCE, CHRISTOPHER R. GAGNE, JILL X. O’REILLY, JANINE BIJSTERBOSCH, SONIA J. BISHOP

**Affiliations:** Institute for Global Health Innovation, Imperial College London, Kensington, London SW7 2AZ, UK; Wellcome Centre for Integrative Neuroimaging, University of Oxford, FMRIB, John Radcliffe Hospital, Oxford, OX3 9DU, UK; Max Planck Institute for Biological Cybernetics, Tübingen, DE; Wellcome Centre for Integrative Neuroimaging, University of Oxford, FMRIB, John Radcliffe Hospital, Oxford, OX3 9DU, UK; Department of Experimental Psychology, University of Oxford, Anna Watts Building, Radcliffe Observatory Quarter, Woodstock Rd, Oxford OX2 6HG, US; Donders Centre for Cognition, Donders Institute, Montessorilaan 3, 6525 HR Nijmegen, NL; Department of Radiology, Washington University School of Medicine, St. Louis, MO, USA; Department of Psychology, UC Berkeley, Berkeley, California 94720, USA; Helen Wills Neuroscience Institute, UC Berkeley, Berkeley, California 94720, USA

**Keywords:** anxiety, ambiguity, probabilistic decision-making, fmri, uncertainty, computational modeling

## Abstract

Theoretical accounts have linked anxiety to intolerance of ambiguity. However, this relationship has not been well operationalized empirically. Here, we used computational and neuro-imaging methods to characterize anxiety-related differences in aversive decision-making under ambiguity and associated patterns of cortical activity. Adult human participants chose between two urns on each trial. The ratio of tokens (‘O’s and ‘X’s) in each urn determined probability of electrical stimulation receipt. A number above each urn indicated the magnitude of stimulation that would be received if a shock was delivered. On ambiguous trials, one of the two urns had tokens occluded. By varying the number of tokens occluded, we manipulated the extent of missing information. At higher levels of missing information, there is greater second order uncertainty, i.e., more uncertainty as to the probability of pulling a given type of token from the urn. Adult human participants demonstrated avoidance of ambiguous options which increased with level of missing information. Extent of ‘information-level dependent’ ambiguity aversion was significantly positively correlated with trait anxiety. Activity in both the dorsal anterior cingulate cortex and inferior frontal sulcus during the decision-making period increased as a function of missing information. Greater engagement of these regions, on high missing information trials, was observed when participants went on to select the ambiguous option; this was especially apparent in high trait anxious individuals. These findings are consistent with individuals vulnerable to anxiety requiring greater activation of frontal regions supporting rational decision-making to overcome a predisposition to engage in ambiguity avoidance at high levels of missing information.

In the 18^th^ Century, Benjamin Franklin wrote that “in this world nothing can be said to be certain, except death and taxes.” This axiom hints at the unease uncertainty can generate. Many of us are troubled by life’s uncertainties. For individuals with clinical or subclinical anxiety, difficulty handling uncertainty is hypothesized to contribute to elevated levels of worry and disruption to daily life (Dugas et al., 1998; Freeston et al., 1994). Indeed, scores on self-report measures of uncertainty intolerance show strong correlations with self-reported anxiety and propensity to worry and are elevated in individuals with anxiety disorders (Carleton, 2012; Carleton et al., 2007; Freeston et al., 1994). However, there is still ongoing debate as to how best to differentiate the constructs of intolerance of uncertainty, intolerance of ambiguity and fear of the unknown and as to whether existing measures assess unitary or multiple constructs (Birrell et al., 2011; Furnham & Marks, 2013; Grenier et al., 2005; Rosen et al., 2014).

Here, we take an alternate approach to examining the relationship between anxiety and the handling of uncertainty. Specifically, we take advantage of the precise operationalization of alternate forms of uncertainty provided by the computational decision-making literature and examine how different forms of uncertainty impact choice behaviors and whether this varies as a function of participants’ trait anxiety levels.

In some situations, for example when flipping an unbiased coin, a point estimate of outcome probability can be calculated precisely. This is often referred to as decision-making under ‘risk’ or first order uncertainty (Ellsberg, 1961). In other situations, it is not possible to calculate a sharp point estimate of outcome probability, i.e. there is second-order uncertainty (Bach et al., 2011). This can occur as a result of both contingency volatility, when outcome probabilities change across time, and contingency ambiguity, when the information required to estimate outcome probabilities is totally or partially missing (Bach et al., 2011; Behrens et al., 2007; Camerer & Weber, 1992; Ellsberg, 1961; Levy et al., 2010). Many of the decisions we make in every-day life are characterized by second-order uncertainty. For example, individual and governmental decision-making during the early stages of the COVID-19 pandemic were complicated both by ambiguity and volatility – lack of testing data and rapidly changing infection levels made it difficult to precisely estimate the probability of infection and transmission linked to different actions. Understanding decision-making under these forms of second-order uncertainty is of clear real-world importance.

In prior work, we have examined decision-making pertaining to threatening potential future outcomes when contingencies are volatile (Browning et al., 2015). We have shown that low trait anxious individuals are able to successfully adapt probabilistic decision-making between stable and volatile conditions but that individuals high in trait anxiety are less able to adapt probabilistic aversive learning to contingency volatility. Here, we use an adaptation of Ellsberg’s classic urn task to extend this work to examine whether high trait anxious individuals show biased probabilistic decision-making under ambiguity when attempting to avoid aversive outcomes and if this is contingent on level of second-order uncertainty as manipulated by varying the extent of missing information.

Healthy individuals have been shown to be ambiguity averse, typically preferring to choose risky options where a point estimate of outcome probability is available than ambiguous options where it is not, even when this is rationally disadvantageous (Bach et al., 2011; Camerer & Weber, 1992; Ellsberg, 1961; Levy et al., 2010). This has mainly been investigated in relation to reward-based decision-making. Hence, an important question is whether individuals also show similar biases in decision-making under ambiguity when outcomes are threat-related and whether this varies across individuals, in particular as a function of trait anxiety. To date, several studies have explored risk aversion in anxiety, for reviews see (Bishop & Gagne, 2018; Hartley & Phelps, 2012). However, there has been little empirical investigation of ambiguity aversion in anxiety, especially for threat-related outcomes. Importantly, many of the tasks used to study ambiguity aversion have used a simple binary comparison of choice under conditions when ambiguity is present (‘ambiguous trials’) or absent (‘risk trials’). This fails to distinguish the influence upon choice of the simple presence or absence of missing information (hereon referred to as ‘categorical’ ambiguity) from that of the level of missing information (hereon referred to as ‘parametric’ or ‘information-level dependent’ ambiguity), Bach et al., (2011). The greater the level of missing information, the broader the potential range of outcome probabilities, i.e., the higher the level of second-order uncertainty.

In the current study, our first aim was to seek to replicate prior findings that individuals become more ambiguity averse as missing information increases (Bach et al., 2011). Second, we aimed to test the hypothesis that this would be particularly true of individuals with elevated trait anxiety. This hypothesis was informed by our prior findings that high trait anxious individuals show poorer ability to adjust to second-order uncertainty produced by contingency volatility (Browning et al., 2015) and by the premise that individuals who struggle with estimating outcome probabilities under second-order uncertainty might be more inclined to avoid engaging with such options.

Our third aim was to determine if elevated trait anxiety is linked to altered processing of parametric ambiguity in medial or lateral frontal cortical regions. Previous work has implicated both medial and lateral subregions of frontal cortex in the normative processing of ambiguity (Hsu et al., 2006; Huettel et al., 2006; Krain et al., 2006; Levy et al., 2010). However, most of these studies have focused on categorical manipulations of ambiguity. As outlined above, increases in both contingency volatility and parametric ambiguity (i.e., extent of missing information) lead to increased imprecision, that is increased distribution breadth, around estimates of outcome probability. The dorsal anterior cingulate cortex (dACC) has been shown to track changes in contingency volatility (Behrens et al., 2007). If a common computational mechanism supports the processing of different instantiations of second-order uncertainty, a common neural substrate might well be expected to underlie both the processing of contingency volatility and parametric ambiguity. This led us to use a region of interest (ROI) based analysis to test the hypothesis that dACC would track changes in level of missing information. In the ambiguity task used in the current study, unlike our previously adopted volatility paradigm (Browning et al., 2015), participants can choose to engage with or avoid options characterized by high levels of second order uncertainty. Hence an important follow-on question was whether dACC activity to missing information at the time of choice would differ between trials where participants went on to choose the ambiguous or unambiguous option and if this would vary as a function of trait anxiety.

We also modelled activity in two further regions of interest. The first of these was the inferior frontal sulcus (IFS). The IFS has been found to be preferentially activated on ambiguous gambles with the IFS response to ambiguous gambles being highest in participants displaying an ambiguity preference (Huettel et al., 2006). Our third region of interest was the rostral lateral prefrontal cortex (RLPFC). Badre and colleagues reported that participants who took second-order uncertainty into account showed increased RLPFC activity to trial-by-trial changes in second-order uncertainty (Badre et al., 2012). We used the co-ordinates from this study to define our RLPFC ROIs.

## RESULTS

### EXAMINING THE EFFECT OF MISSING INFORMATION ON PROBABILISTIC DECISION-MAKING

**Figure 1:**
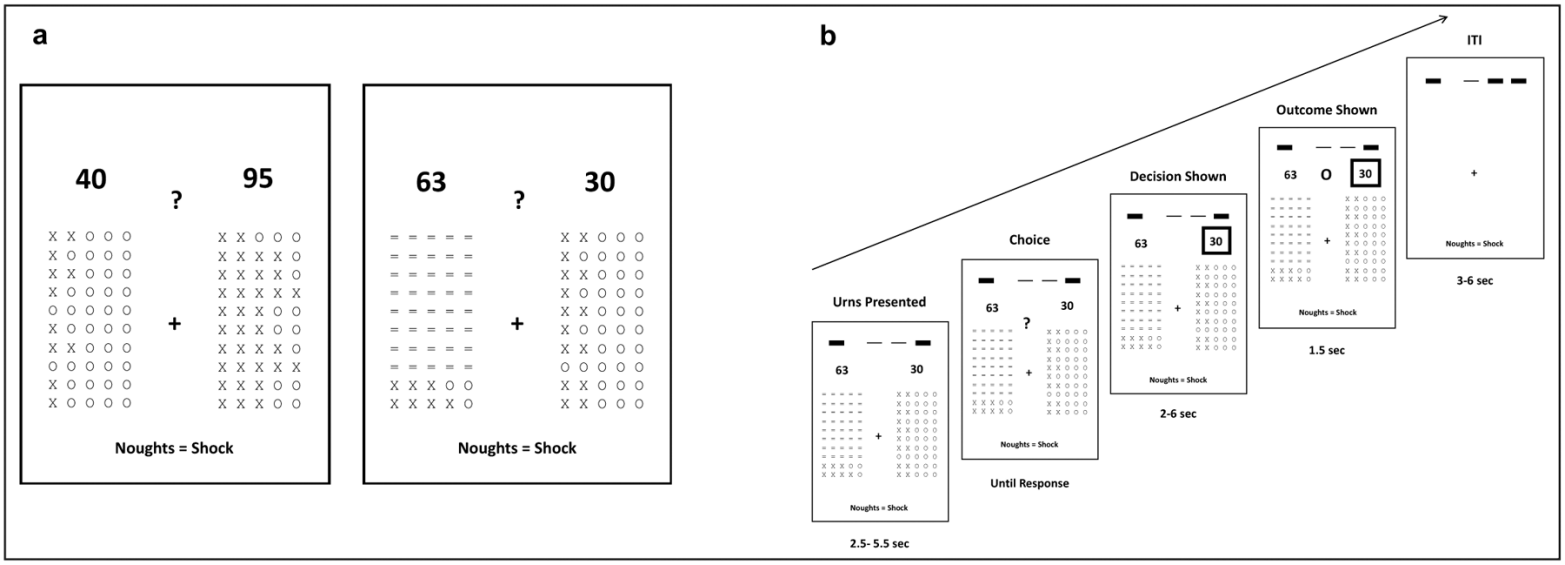
Decision-Making under Ambiguity Task. **(a).** Example Unambiguous Trial (left) and Ambiguous Trial (right). Participants chose one of two ‘urns’ from which to draw a token. The proportion of ‘X’s and ‘O’s varied between urns and across trials. The number above each urn (1–150) indicated the magnitude of electric shock that might be received if an ‘O’ was drawn. On 52% of trials (‘ambiguous trials’), a number of tokens in one urn (the ‘ambiguous urn’) were replaced by a “=” symbol. 10, 30, 40, 45, 46, 47, 48 or 49 of the 50 tokens were obscured. **(b).** Trial sequence and timing. Urn presentation (2.5–5.5s) was followed by a question mark (“?”) which indicated that participants could choose one of the two urns. Their decision was indicated by a square placed around the chosen urn magnitude. After a variable interval (2–6s), a token was randomly drawn from the chosen urn and displayed for 1.5s. There was a 3–6s interval prior to the next trial. Trial outcomes were stored across each block of five trials. At the end of the block, one outcome was selected at random; if an ‘O’ outcome was selected, this was delivered as an electric shock of corresponding magnitude (see Figure S1). During each block, stored outcomes were displayed by means of a summary histogram at the top of the screen (bins from left to right: ‘X’ outcome, ‘O’ outcome of magnitude 1–50, ‘O’ outcome of magnitude 51–100, ‘O’ outcome of magnitude 101–150).

We recruited 41 healthy adults aged between 18 and 40 years with varying levels of trait anxiety as indexed by the Spielberger State Trait Anxiety Inventory trait subscale (STAI-trait; Spielberger et al., 1983), see Methods. Participants performed an adaptation of Ellsberg’s classic urn task while functional magnetic resonance imaging (fMRI) data were collected, see Figure 1. Complete behavioral and fMRI datasets were obtained from thirty-three participants, see Methods. Data from two further participants were excluded due to outlying behavior and movement within the scanner. The final sample comprised 31 adults (21 females, 10 males; age:18–38 years, M = 21.6, SD = 4.1; STAI-trait scores: 20 to 53, M = 36.7, SD = 9.6).

The task comprised 200 trials; on each trial, participants were asked to choose between two 50 token urns. Each urn contained a different proportion of ‘X’ and ‘O’ tokens; the proportions were reset on each trial. A token was drawn randomly from the selected urn. An ‘O’ resulted in potential receipt of shock, see Methods. The magnitude of potential shock was indicated above each urn, see Figure 1. This magnitude value also varied between urns and was reset between trials. Forty-eight percent of trials (n = 96) were ‘unambiguous’ or ‘risky’. On these trials, all tokens were revealed in both urns. The remaining trials (n = 104) were ‘ambiguous’, that is one urn had a varying number of tokens obscured. Equal numbers of ambiguous trials had 10, 30, 40, 45, 46, 47, 48 or 49 tokens of one urn obscured.

Optimal rational behavior on this task can be achieved by participants using the revealed tokens as samples to conduct Bayesian inference about the underlying probability of drawing an ‘O’. Specifically, Pa, the probability of drawing an O if the ambiguous urn is selected, can be estimated as E(p), p~Beta(1+k, 1+n−k), where n is the total number of tokens revealed and k is the number of Os revealed. Changes in posterior uncertainty per token revealed are greatest when the majority of tokens are obscured, with an increase in one token revealed having a far greater impact when less than five tokens are revealed than when 30 or 40 tokens are revealed. We took this into account both in terms of our manipulation of number of tokens obscured and our definition of missing information. Missing information (‘*A*’) was defined as 1– √ (n/50) so that it would be non-linear in terms of number of tokens obscured but approximately linear in terms of the increase in posterior uncertainty. On ambiguous trials, outcome probability and magnitude for the ambiguous and unambiguous urn were manipulated orthogonally to missing information. Each participant received the same trial order with a pseudo-randomized interleaving of ambiguous and unambiguous trials.

### THE EFFECT OF MISSING INFORMATION ON CHOICE: MODEL FREE ANALYSIS

**Figure 2:**
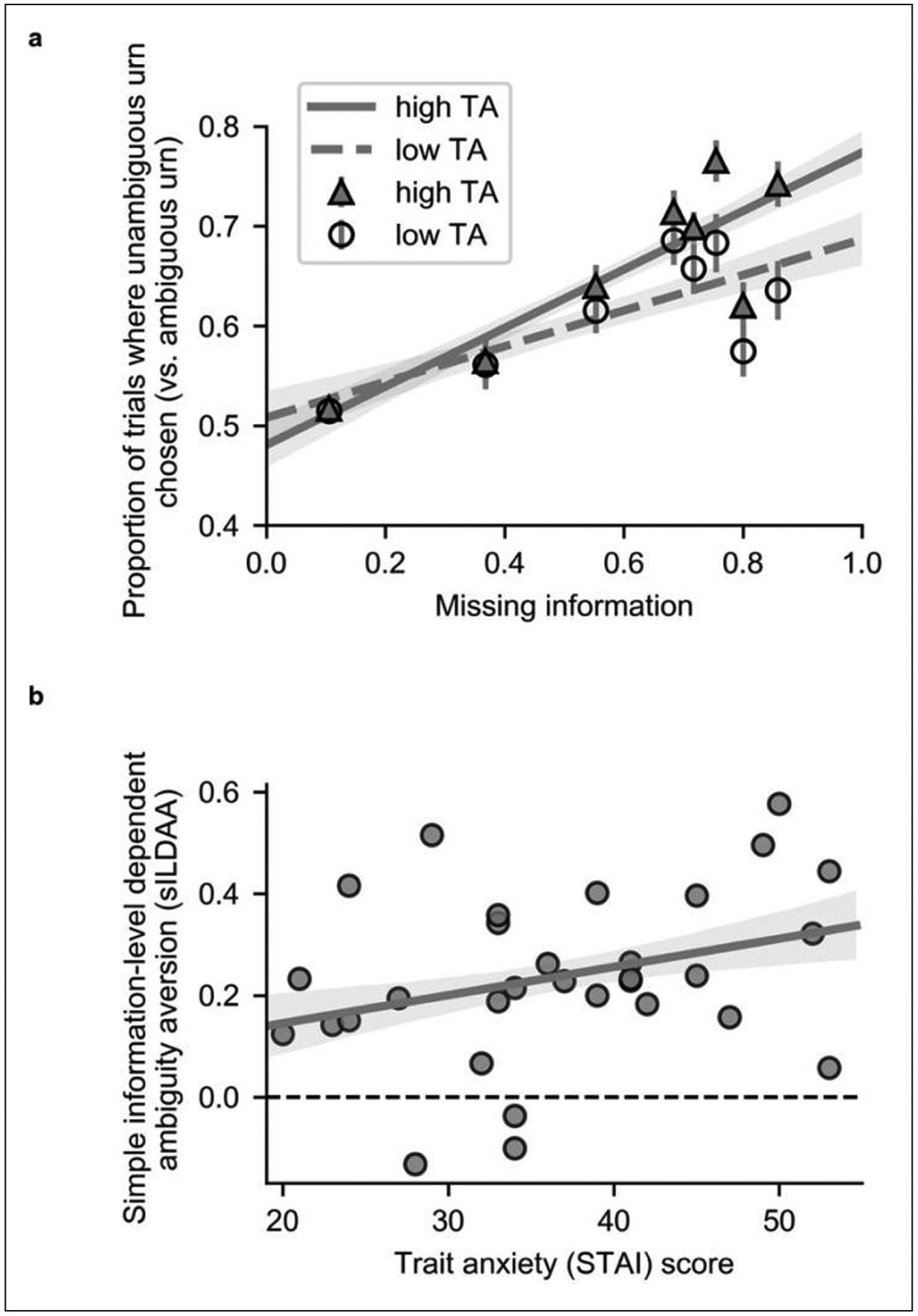
Model-free analysis reveals high trait anxious individuals show greater avoidance of ambiguous urns as a function of missing information. **(a)** The proportion of ambiguous trials on which participants chose the unambiguous urn (i.e., avoided the ambiguous urn) is plotted against missing information, *A*, where *A =* 1− √ (n/50). Participants were divided into two groups using a median split on STAI trait anxiety (TA) scores for illustrative purposes. **(b)** Simple Information-Level Dependent Ambiguity Aversion (s-ILDAA) is defined as the slope of the regression of the proportion of ambiguous trials on which the unambiguous urn is chosen against missing information (i.e., the slope of the regression shown in Figure 2a). Larger values are indicative of greater avoidance of ambiguity as a function of missing information. Here, s-ILDAA values for each participant are correlated against STAI trait anxiety. In line with predictions, elevated trait anxiety was associated with increased avoidance of the ambiguous urn as a function of missing information, r (29) = 0.3168; p = 0.041, Pearson, one-tailed; rho(29) = 0.33, p = 0.036, Spearman, one-tailed. Note: Shaded regions represent +/− one standard error in the regression coefficients (obtained by resampling the data 10,000 times with replacement).

Prior to computationally modeling participants’ choice behavior, we conducted a simple model-free analysis of the influence of extent of missing information upon choice. As detailed above, on ambiguous trials, outcome probability and magnitude for the ambiguous and unambiguous urn were manipulated orthogonally to missing information (*A*). Hence, it is possible to obtain a model-free measure of information-level dependent ambiguity aversion by examining how the proportion of trials on which the unambiguous urn is selected (i.e., the ambiguous urn avoided) varies as a function of missing information (*A*). Figure 2a show the relationship between missing information (*A*) and the proportion of trials on which the unambiguous urn was selected; for illustrative purposes, we show this for participants grouped by a median split on STAI trait anxiety scores. The slope of the regression of proportion of trials on which the unambiguous urn was chosen against missing information gives a simple measure of information-level dependent ambiguity aversion (s-ILDAA). To test the hypothesis that elevated trait anxiety is linked to increased avoidance of ambiguity as a function of missing information, we correlated s-ILDAA values against participant STAI trait anxiety scores; as predicted, this gave a significant positive correlation, r(29) = 0.3168; p = 0.041, Pearson, one-tailed; rho(29) = 0.33, p = 0.036, Spearman, one-tailed, Figure 2b. Note, s-ILDAA values were normally distributed; we also give the Spearman correlation to facilitate comparison with correlations of anxiety against model parameters, see next section, where non-parametric correlations were used due to non-normal distribution of parameter values.

### MODELING EFFECTS OF MISSING INFORMATION ON CHOICE: MODEL SELECTION

On any given trial, there are multiple variables that might influence participants’ choice behavior including the potential magnitude of shock linked to each urn, the ratio of revealed tokens in each urn, the presence or absence of ambiguity and, for ambiguous urns, the level of missing information. By modeling the influence of these parameters on participants’ choice and examining how this varies as a function of trait anxiety we can gain a better picture of the influence of trait anxiety upon decision-making under ambiguity.

**Figure 3:**
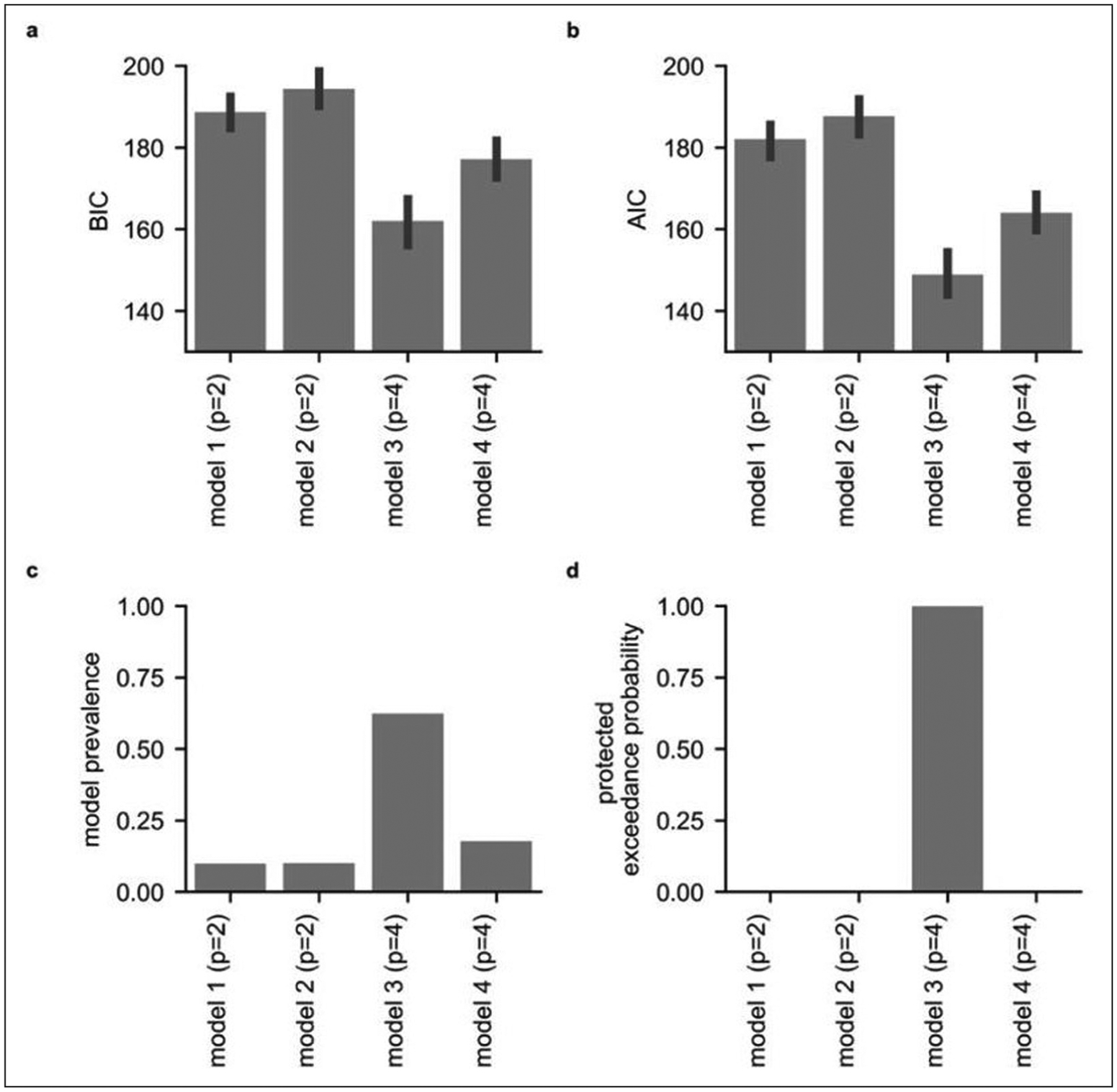
Model comparison results for the four main models. Panels **a** and **b** show penalized log-likelihood values for each model averaged across participants. In panel **a**, the results shown are for model comparison using the Bayesian information criterion (BIC); this penalizes models with a higher number of parameters more strictly than the Akaike information criterion (AIC). Results from model comparison using AIC are shown in panel **b**. Panels **c** and **d** show the results from Bayesian model selection (BMS; Stephan et al., 2009), see Methods. The BMS estimate of model prevalence, that is the population-level estimate of the proportion of participants best fit by each model, is given in Panel **c**. Panel **d** displays the protected exceedance probability for each model; this is the probability that each model is the most prevalent at the population level, i.e., that it is the most likely to explain behavior on the task. For trial-wise model fits see Figures S13 and S14.

We used model comparison to inform our parameterization of participants’ behavior on the task. We tested whether participants’ choice behavior was better captured by a model that included separate parameters for the influence of the relative probability of drawing an ‘O’ from each urn upon choice and for the influence of the relative magnitude of shock associated with each urn upon choice (Model 1, see Methods) or by a model in which the expected utility (EU, the weighted product of outcome magnitude and probability) of each urn was estimated prior to comparison between urns (Model 2, see Methods). We compared the fit of these baseline models against equivalent models (Models 3 and 4, respectively, see Methods) that additionally captured categorical ambiguity avoidance or ambiguity seeking, i.e., preference of unambiguous urns over ambiguous urns, or vice versa, and information-level dependent ambiguity avoidance/seeking (ILDAA), i.e., avoidance or seeking of ambiguous urns as a function of the level of missing information. Figure 3 gives the mean Bayes information criterion (BIC) and Akaike information criterion (AIC) penalized log-likelihoods for each model (panels a and b, respectively).

We compared the fit of Models 1 to 4 using Bayesian model selection (BMS; Stephan et al., 2009); this uses hierarchical Bayesian inference to estimate the prevalence of each model at the population level and to test statistically whether any one model is more prevalent than the others. This approach can reveal if different groups of participants are best fit by different models. BMS requires an estimate of the log model evidence for each model for each participant; here, we used BIC penalized log-likelihood values (Stephan et al., 2009). This analysis revealed that Model 3 (presented in Box 1) performed best overall, having the highest population-level prevalence and a protected exceedance probability close to 1 (Figure 3c, 3d).

We next examined whether relative model fit changed between the first and second half of the task; model 3 performed best across both halves of the task (Figure S2). Finally, we also used a median split on participants’ STAI trait scores and reconducted model comparison for the two resultant participant sub-groups. Model 3 performed best for data from both low and from high trait anxious participants, considered separately (Figure S3).

We note that the results of our model comparison did not favor models in which expected utility was estimated prior to urn comparison. Instead, participants’ behavior was better modeled by separate comparison of the two urns in terms of outcome probability and outcome magnitude. The occlusion of some of the tokens needed to estimate outcome probability for ambiguous urns may potentially have promoted a strategy of estimating outcome probabilities separately to outcome magnitudes to avoid having to integrate missing information, numbers of ‘X’s and ‘O’s revealed, and outcome magnitudes in a single step. It might then have been simpler for the participant to also apply this strategy to the interleaved unambiguous trials. Alternatively, the difference in the nature of information presentation – simple numerical presentation of outcome magnitude versus a display of varying numbers of ‘X’s and ‘O’s for outcome probability might have sufficed to drive separate evaluation of outcome probability and outcome magnitude. We note that using a distinct probabilistic learning under volatility task where there is no missing information but participants have to learn outcome probabilities from trial outcomes, our most recent modeling also supports separate evaluation of outcome probability and outcome magnitude (Gagne et al., 2020).

We compared the winning model, Model 3, against a number of additional models of potential interest (see the Supplementary Modeling Note). We used this additional modeling to further investigate the influence of ambiguity on behavior by examining the impact of removing the beta binomial correction to the calculation of P_a_ or of removing the parameters capturing either categorical ambiguity avoidance (β_0_) or increased avoidance of the ambiguous urn as a function of missing information level (β_3_). In addition, we investigated whether the presence or level of missing information influenced the weighting given to probability or magnitude information. We also explored whether participants’ performance could be explained by individual differences in the reliance on pessimistic or optimistic priors when faced with missing information. For the results of this more extensive model comparison see Supplementary Figures 4–7. None of the additional models significantly out-performed model 3.

### PARAMETER RECOVERY AND MODEL IDENTIFICATION ANALYSES

Parameter recovery analyses were conducted for models 1 to 4 using standard procedures (see Methods for full details). For each model, a range of possible values was selected for each parameter and new parameter values were chosen randomly from across this range. This was repeated 100 times and each set of ‘ground truth’ parameter values used to generate a simulated dataset.

The model in question was then fit to these 100 simulated datasets and parameter values were re-estimated or ‘recovered’. These recovered parameter values were correlated against their ground truth values. For model 3, recovered parameter values showed strong correlations with ground truth parameter values, rs (98) > 0.9, Pearson 2-tailed, confirming that the model parameters were recoverable, Figure S8. This was also the case for model 1 (i.e., the base model version of model 3 omitting parameters for categorical and parametric ambiguity). For the two EU models, models 2 and 4, parameter recovery was moderate but not as good, Figure S8.

We also conducted a model identification analysis; this reveals how often model comparison, using BIC-penalized log-likelihood values, correctly selects the model that was used to simulate the data (i.e., the true model). Here, we used the 100 simulated datasets generated for models 1 to 4, as described above. All four models were fit to each dataset and the best fitting model (i.e., the model with the lowest BIC-penalized log-likelihood value) was determined. The proportion of times that each model was identified as the best- fitting model was assessed. Model identification performance is indexed by the frequency with which the model that was used to generate the data (i.e., the true model) is correctly selected as the best-fitting model. The winning model, model 3, was identified as the best fitting model 89% of the time when it was indeed used to generate the data, Figure S9. Models 1, 2 and 4, were also correctly identified on over 80% of occasions, Figure S9. This analysis confirms that these four models make sufficiently distinct predictions in our task to be distinguishable from one another on the basis of participants’ choice data.

### MODELING CHOICE BEHAVIOR: GROUP-LEVEL RESULTS

Here we report the results obtained by fitting model 3 to participants’ choice behavior. In line with prior findings from reward-based ambiguity tasks (Camerer & Weber, 1992; Hsu et al, 2006; Levy et al., 2010), participants’ choice behavior showed a bias towards ambiguity aversion. A large majority of participants had positive beta values for both categorical ambiguity avoidance (β_0_, 30 out of 31) and information level dependent ambiguity avoidance (β_3_, 28 out of 31). At a group level, values for both parameters differed significantly from 0, β_0_: t (30) = 7.9, p = 8.6e-9; β_3_: t (30) = 6.0, p = 1.4e-6, two- tailed; see Table S1 for parameter means. This indicates that participants preferentially chose the unambiguous urn over the ambiguous urn (indexed by β_0_), with this bias increasing as a function of the level of missing information (indexed by β_3_). As predicted under aim 1, this group-level result replicates prior findings (Bach et al., 2011). As expected, participants’ decisions were also strongly informed by both the difference in magnitude (β_1_) and probability (β_2_) of urn outcomes, β_1_: t (30) = 11.2, p = 2.9e–12; β_2_: t (30) = 13.6, p = 2.5e–14, two-tailed.

Model parameters deviated from normal distributions, Figure S10, Table S2. We note that t-tests are fairly robust to violations of normality; hence we report t-test results here and non-parametric test results in Table S2. In each case, the non-parametric test results replicated the findings reported above. Given the potentially greater influence of outliers on correlational analyses, we used non-parametric statistical tests for the correlation analyses reported below.

### EFFECTS OF TRAIT ANXIETY UPON BEHAVIOR

**Figure 4:**
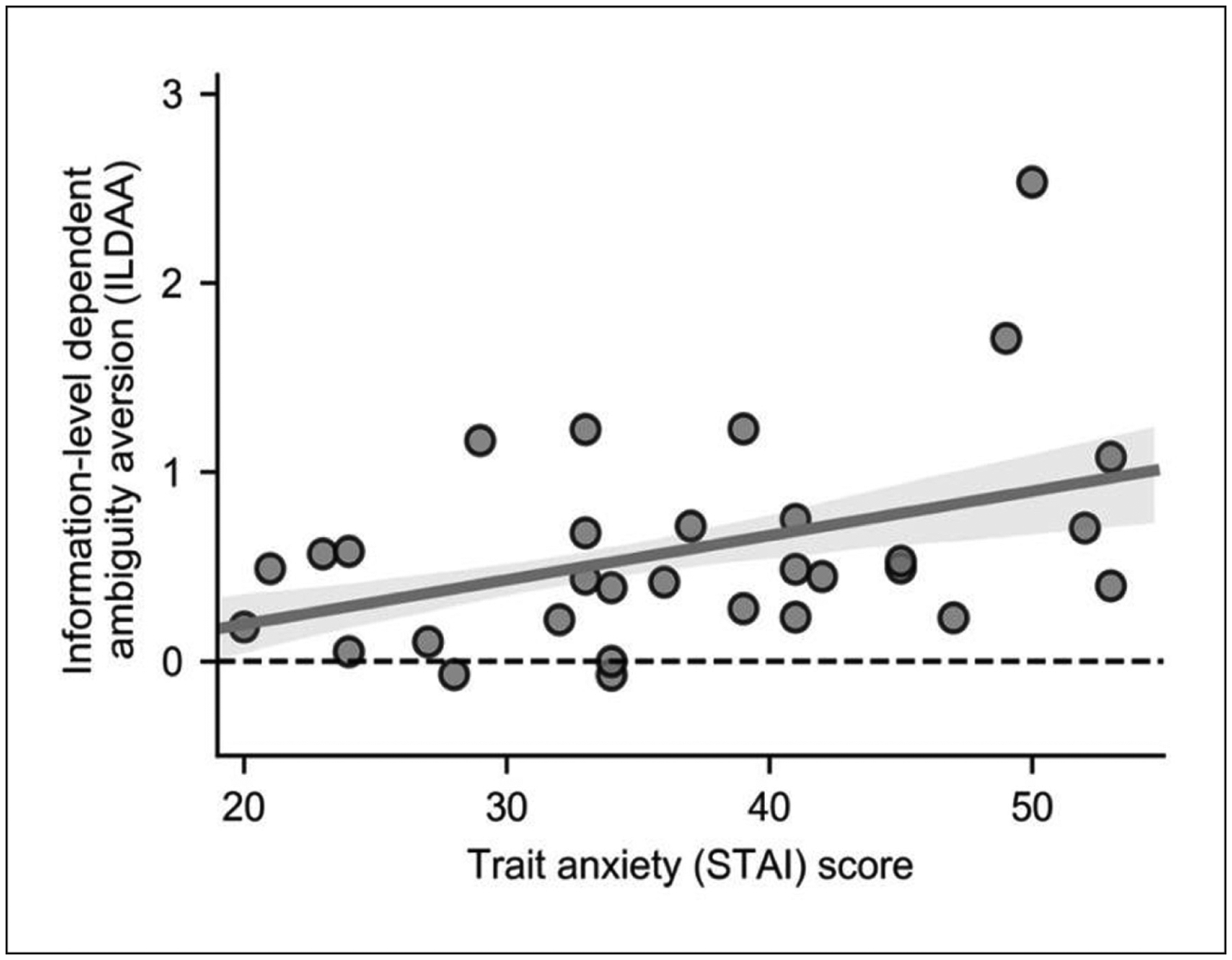
Information-Level Dependent Ambiguity Aversion (ILDAA), as indexed by β_3_, is positively correlated with trait anxiety, rho (29) = 0.36, p = 0.023, Spearman, one-tailed. In model 3, the influence of missing information on selection of the unambiguous urn is captured by parameter estimates for β_3_; large positive values indicate greater ambiguity avoidance as a function of missing information. In this model, parameters are also included to control for the influence upon choice of difference between the two urns in outcome probability, outcome magnitude and categorical ambiguity (the presence or absence of ambiguity). The majority of participants showed increased avoidance of the ambiguous urn as level of missing information increased (this holds for all data points above the dotted line, 28/31 participants). The expected value of unambiguous and ambiguous urns was approximately matched across trials within information levels. Hence, rational behavior is associated with an ILDAA value of zero, or close to zero. Note: Shaded regions represent +/− one standard error in the regression coefficients (obtained by resampling the data 10,000 times with replacement).

Our second main aim was to determine if elevated trait anxiety was associated with increased avoidance of the ambiguous urn as a function of level of missing information; this was indeed the case: (β_3_), rho (29) = 0.36, p = 0.023, Spearman, one-tailed, Figure 4. The results from this analysis support those from our model-free analysis, Figure 2b. In contrast, there was not a statistically significant relationship between trait anxiety and baseline categorical ambiguity aversion (β_0_), rho (29) = 0.15, p = 0.21, Spearman, one-tailed. These findings suggest that high trait anxious individuals incrementally engage in non-rational choice behavior as level of missing information increases.

For those participants who showed ambiguity avoidant behavior that increased with level of missing information (i.e., a positive value for β_3_; n = 28 of 31), we observed a near-significant correlation between the extent of this information-level dependent ambiguity avoidance (β_3_) and cumulative shock received across ambiguous trials, rho (26) = 0.31, p = 0.056, Spearman, one-tailed.

This is consistent with the sub- optimality of information-level dependent ambiguity avoidance (ILDAA). Trait anxiety, itself, was positively correlated with cumulative shock scores across ambiguous trials within these participants, rho (26) = 0.37, p = 0.026, Spearman, one-tailed.

Heightened trait anxiety was also associated with a greater influence of difference in outcome probability between urns on choice behavior (β_2_), rho (29) = 0.37, p = 0.043, (Spearman, two-tailed), Supplementary Figure 11a. There was no significant relationship between trait anxiety and the influence of difference in outcome magnitude between urns on choice behavior (β_1_), p = 0.99, (Spearman, two-tailed), Supplementary Figure 11b.

### fMRI RESULTS

FMRI data collected during performance of the urn task was preprocessed using the Human Connectome Project standardized pre-processing pipeline (see Methods). General linear regression was used to model voxel-wise Blood Oxygen Level Dependent (BOLD) activity yoked to each stage of each trial (see Methods). Baseline regressors were included that indicated whether a given trial was ambiguous (AT) or unambiguous (UT). The computational model used to fit behavioral performance on the task (model 3) guided the inclusion of additional parameters. For both ambiguous and unambiguous trials, we included regressors for difference in outcome magnitude and the log modulus of difference in outcome probability between urns (using the beta-binomial correction for P_a_ described earlier). We also included a regressor for level of missing information on ambiguous trials *(A*, also as described earlier). Additional regressors indicated the outcome of each trial, signed and unsigned outcome surprise, and the outcome selected for delivery at the end of each block of 5 trials (see Methods for further details of these additional regressors). Each of these regressors was convolved with the hemodynamic response function. Nuisance regressors (e.g., for movement) were also included (see Methods). In an additional model, we further broke down ambiguous trials into those where the ambiguous urn was selected and those where the unambiguous urn was selected.

We conducted region-of-interest (ROI) based fMRI analyses and supplementary whole brain analyses (see Supplementary Analyses: fMRI analyses). BOLD activity associated with each parameter or contrast of interest was averaged within each of our five regions of interest (dACC, left and right IFS, and left and right RLPFC), see Methods. We focused on activity during the decision-making period (i.e., yoked to the ‘Urns Presented’ time), see Figure 1 and Methods. In particular, we examined changes in ROI activity as a function of the presence and level of missing information. We controlled for trial-wise differences in the relative log modulus probability of drawing an ‘O’ from each urn and in the relative outcome magnitude of each urn by inclusion of these parameters in the model. We also modeled the influence of these and other parameters (binary outcome, outcome magnitude, outcome surprise and signed surprise) upon ROI activity at outcome presentation (see Methods). We used Bonferroni correction to control for multiple comparisons across ROIs.

### GROUP-LEVEL RESULTS

We first investigated the response to categorical ambiguity in each of our ROIs. Here, we compared BOLD activity yoked to urn presentation for ambiguous versus unambiguous trials (AT-UT). Bilateral IFS showed significantly higher activity on ambiguous trials, left: t(30) = 7.1, p = 6.9e–8, p_corr_ = 3.5e–7, two-tailed, right: t(30) = 4.3, p = 1.9e–4, p_corr_ = 9.3e–4, two-tailed, as previously reported by Huettel and colleagues (Huettel et al., 2006). Activation differences between ambiguous and unambiguous trials in the other ROIs did not survive correction for multiple comparisons.

We next investigated the response to parametric ambiguity in each of our ROIs. Following findings reported by Behrens and colleagues (Behrens et al., 2007), we tested the hypothesis that, on ambiguous trials, dACC activity yoked to urn presentation would track level of second order uncertainty, here operationalized as level of missing information (*A*). In addition, we conducted parallel analyses for the IFS and RLPFC ROIs. Activity in both dACC and right IFS increased linearly as a function of the level of missing information, dACC: t (30) = 3.5, p = 0.0015, p_corr_ = 0.0074, two-tailed, right IFS: t (30) = 3.8, p = 7.4e–4, p_corr_ = 0.0037, two-tailed. The effect of missing information on activity at urn presentation was not significant in left or right RLPFC and did not survive correction for multiple corrections in left IFS, t (30) = 2.5, p = 0.019, p_corr_ = 0.093, two-tailed.

Regions responsive to the level of missing information might simply be engaged in monitoring the level of second-order uncertainty. However, increased activation as a function of missing information might also be seen in regions supporting ambiguity aversion or in those engaged in overcoming ambiguity aversion and rationally evaluating the options at hand. To further explore the dACC and IFS response to missing information, we divided ambiguous trials on the basis of participant choice. We fitted a new model to participants’ BOLD data in which we separately modeled ambiguous trials where participants went on to choose the unambiguous urn (unambiguous chosen, UC) and ambiguous trials where participants went on to choose the ambiguous urn (ambiguous chosen, AC), see Methods. Across participants, dACC and IFS activation yoked to urn presentation increased as a function of missing information level on trials where participants subsequently chose the ambiguous urn, dACC: t (30) = 3.0, p = 0.0054, p_corr_ = 0.027, left IFS: t(30) = 3.8, p = 0.00059, p_corr_ = 0.0030, right IFS: t(30) = 4.8, p = 4.2e–5, p_corr_ = 2.1e–4, all two-tailed. The dACC and IFS response to missing information level was weaker on trials where participants chose the unambiguous urn, dACC: t (30) = 1.8, left IFS: t (30) = 1.5, right IFS: t (30) = 2.1, ps_corr_ > 0.1, two-tailed, in line with the contention that the dACC and IFS response to parametric ambiguity might support overcoming ambiguity aversion. However, the difference in response to missing information by trial type did not reach significance, dACC: t (30) = 1.3; left IFS: t (30) = 1.2, right IFS: t (30) = 1.6, ps > 0.1, two-tailed. Additional analyses revealed that neither right nor left RLPFC showed a significant difference in activity to missing information as a function of urn choice.

### EFFECTS OF TRAIT ANXIETY UPON THE PREFRONTAL CORTICAL RESPONSE TO MISSING INFORMATION

**Figure 5:**
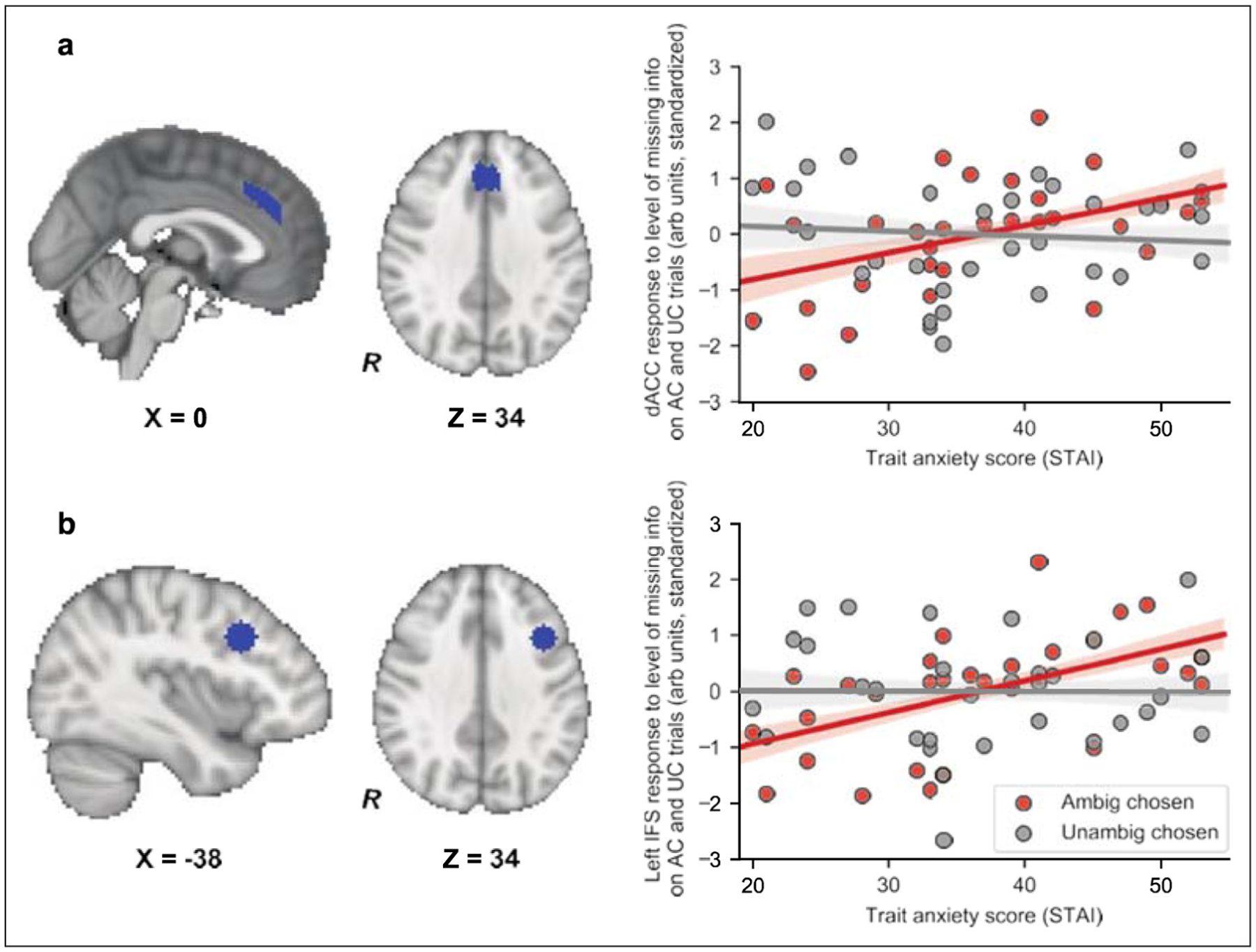
Trait anxiety was linked to increased dACC and IFS activation as a function of missing information on trials where the ambiguous urn was subsequently chosen. **(a)**. Left: Sagittal and axial views of the dorsal anterior cingulate (dACC) ROI. Right: We divided ambiguous trials according to whether participants chose the ambiguous or unambiguous urn (see Methods). The extent to which mean dACC activation, time-locked to urn presentation, varied as a function of level of missing information (*A*) was estimated for each participant for each trial type. The resulting z-score values are plotted against participant trait anxiety level (red circles: Ambiguous urn chosen trials; grey circles: Unambiguous urn chosen trials). Trait anxiety was positively correlated with dACC activation to missing information level on Ambiguous chosen trials, rho (29) = 0.48, p = 0.0061, p_corr_ = 0.031, but not on Unambiguous chosen trials, rho (29) = −0.09, p > 0.1. **(b)** Left: Sagittal and axial views of the left Inferior Frontal Sulcus ROI (IFS). Right: Z-scores for the mean left IFS response to level of missing information (*A*) at time of urn presentation are plotted against participant trait anxiety (red circles: Ambiguous urn chosen trials; grey circles: Unambiguous chosen trials). Trait anxiety was positively correlated with left IFS activation to missing information level on Ambiguous chosen trials, rho (29) = 0.60, p = 3.9e–4, p_corr_ = 0.0020, but not on Unambiguous chosen trials, rho (29) = −0.04, p > 0.1. Note. All correlations reported are Spearman, two-tailed. Shaded regions represent +/− one standard error in the regression coefficients (obtained by resampling the data 10,000 times with replacement).

The group-level analyses reported above indicated that the parametric response to missing information in dACC and IFS was strongest on trials where participants went on to select the ambiguous urn. Correlational analyses revealed that this pattern was amplified in high trait anxious individuals. On trials where participants chose the ambiguous urn, trait anxiety was significantly positively correlated with the response to level of missing information in both dACC and left IFS, dACC: rho (29) = 0.48, p = 0.0061, p_corr_ = 0.031, Spearman, two-tailed, left IFS: rho (29) = 0.60, p = 3.9e-4, p_corr_ = 0.0020, Spearman, two-tailed, Figure 5. No equivalent relationship was observed in either dACC or left IFS on trials where the unambiguous urn was selected, dACC: rho(29) = −0.09, left IFS: rho(29) = −0.04, ps > 0.1, Spearman, two- tailed, Figure 5, and the difference in parametric activation to missing information as a function of whether the ambiguous or the unambiguous urn was selected significantly increased with trait anxiety in both regions, dACC: rho(29) = 0.36, p = 0.049, Spearman, two-tailed, left IFS, rho(29) = 0.41, p = 0.021, Spearman, two-tailed. When collapsing across all ambiguous trials, only dACC activity to missing information showed a relationship with trait anxiety and this effect did not survive correction for multiple comparisons, rho (29) = 0.36, p = 0.044, pcorr = 0.22, Spearman, two-tailed.

We conducted an additional analysis of RLPFC activity across all ambiguous trials and for ambiguous trials broken down by urn selected. Here we had fewer clear hypotheses given the lack of a group-level RLPFC response to missing information across all trials, to missing information as a function of urn selected, or indeed to categorical ambiguity. Across all ambiguous trials, trait anxiety was associated with an increased parametric response to missing information in left RLPFC, r (29) = 0.50, p = 0.0038, p_corr_ = 0.019, Spearman, two-tailed, see Figure S12. A similar effect was also observed in right RLPFC but this effect did not survive correction for multiple comparisons (right RLPFC: r (29) = 0.39, p = 0.032, p_corr_ = 0.16). In both left and right RLPFC, the relationship between trait anxiety and response to missing information did not vary significantly as a function of urn chosen (ps > 0.2, Spearman, two-tailed).

### ADDITIONAL RESULTS

Trait anxiety was not significantly correlated with the response to the categorical presence of ambiguity (AT-UT) in any of our regions of interest, ps > 0.1, Spearman, two-tailed. Results from a supplementary ROI analysis using an alternate method for defining ROIs (group-level contrasts orthogonal to the information-level contrast of interest) are provided in the Supplementary Information (see Supplementary Analyses: fMRI analyses). We note that the results from these alternate analyses for dACC and IFS are highly consistent with those reported here. Insufficient RLPFC activity to the group-level contrast was obtained to define RLPFC ROIs in this manner.

Given the positive relationship between trait anxiety and both information-level dependent ambiguity aversion and increased dACC and IFS activity to missing information, in particular on trials where the ambiguous option was engaged with, we conducted a supplementary analysis where we directly examined the relationship between ILDAA scores and activity in our regions of interest as a function of missing information level. Here, we observed that higher ILDAA scores were associated with increased dACC activity as a function of level of missing information; rho (29) = 0.488, p = 0.0059, p_corr_ = 0.030, Spearman, two-tailed. Other associations did not survive correction for multiple comparisons; for further details, see Supplementary Analyses: fMRI analyses.

We were not primarily interested in activity at time of outcome presentation. However, for completeness, we present outcome time analyses in the Supplementary Information (see Supplementary Analyses: fMRI analyses). We note that no significant effects of trait anxiety were observed for outcome-yoked activity in any of our frontal ROIs. For these outcome time analyses, we also examined activity within subcortical ROIs, namely the amygdala and nucleus accumbens. Here activity was found to vary as a function of trait anxiety (see Supplementary Analyses: fMRI analyses.) Given these exploratory analyses were not a-priori planned nor corrected for the total number of ROIs examined we do not discuss these findings further here.

## DISCUSSION

We are faced daily with decisions rendered challenging by missing information. In addition, the amount of information available to inform our decisions can vary considerably. In the current study, we demonstrate for the first time that elevated anxiety is associated with increased behavioral avoidance of ambiguous options as a function of the level of missing information. This quantifiable sensitivity of trait anxious participants to missing information might lead to suboptimal behavioral choices and potentially underlie the distress and maladaptive decision-making shown by anxious individuals when confronted with ambiguous situations in everyday life.

Our fMRI findings further reveal that decision-making under ambiguity is linked to activation of frontal cortical regions; activity in both IFS and dACC increase with the level of missing information. Dividing trials according to participants’ choice behavior showed that increases in IFS and dACC activity with extent of missing information was primarily observed on trials where participants went on to choose the ambiguous urn. This differential activation was strongest in high trait anxious individuals. These results are consistent with a network of frontal regions facilitating rational evaluation of options under second order uncertainty and being recruited to override engagement in ambiguity avoidance. The extent of activation required to support choice of the ambiguous urn was greatest for high trait anxious participants faced with high levels of missing information (i.e., when faced with conditions where they disproportionally engaged in ambiguity avoidance.)

Whereas several empirical studies have investigated the link between trait anxiety and risk aversion (for reviews, see Bishop & Gagne, 2018; Hartley & Phelps, 2012), the current study is the first to examine if quantifiable differences in decision-making under ambiguity are associated with heightened trait anxiety. Our group-level findings indicate that participants show both categorical ambiguity aversion and increased avoidance of the ambiguous urn as a function of level of missing information. Importantly, the extent to which participants showed increased avoidance of ambiguous options as a function of missing information was found to correlate significantly with participant trait anxiety. This bias towards avoiding options where little information is available was not rational as missing information was varied orthogonally to the probability difference and outcome magnitude difference between urns.

In previous work, we have shown that trait anxiety is also associated with an inability to adapt learning rate to match contingency volatility (Browning et al., 2015). Contingency volatility is another form of second-order uncertainty that, similar to missing information, reduces the ability to precisely calculate point estimates of outcome probabilities. In light of these earlier results, our current data suggest that a reduced capacity to factor second-order uncertainty into the decision process might reflect a common pathological mechanism underlying impoverished decision-making under conditions of both volatility and ambiguity in anxiety. If this is the case, we might also expect a common neural substrate to be activated by second-order uncertainty across both empirical manipulations of volatility and level of missing information.

Behrens and colleagues investigated the neural mechanisms supporting adaptation of learning to contingency volatility (Behrens et al., 2007). They reported that a dorsal region of anterior cingulate cortex (dACC) showed activity that positively correlated with trial-wise levels of volatility. In our current study, we find that dACC activation shows a significant positive correlation with level of missing information. This is consistent with dACC responding to second-order uncertainty regardless of whether the source of this second-order uncertainty is contingency volatility or missing information. IFS showed a similar pattern of activation while also responding significantly to the categorical presence versus absence of ambiguity.

In the volatility task used by Behrens and colleagues and in our own prior work (Behrens et al., 2007; Browning et al., 2015), volatility varies across trials and participants are unable to choose to avoid it. In contrast, in the task used in the current study, participants are able to choose between ambiguous and unambiguous options, whenever ambiguity is present. This enabled us to explore whether dACC and IFS activation to missing information was primarily observed on trials where participants went on to select or avoid the ambiguous option. We found that increases in dACC and IFS activation as a function of missing information, time-locked to urn presentation, were primarily observed on trials where participants went on to choose the ambiguous urn. This is consistent with a role for these regions in overcoming ambiguity aversion.

Ambiguity aversion has been posited to stem from the transfer of an often ecologically valid heuristic, “avoid betting when you lack information others may have”, to situations where this heuristic is sub-optimal (Frisch & Baron, 1988). A bias towards ambiguity aversion might reflect the triumph of an ambiguity avoidance heuristic over rational evaluation of the probability and magnitude of potential outcomes given selection of the ambiguous versus unambiguous urn. Our data indicate that ambiguity avoidance is greatest on trials with high missing information and that this is particularly true for high trait anxious individuals. Prior work has implicated the dACC and IFS in rational evaluation of alternate courses of action (de Berker et al., 2019; Kim et al., 2009; Kolling et al., 2016; Rogers et al., 1999) including integration of outcome probabilities and magnitudes (Boorman et al., 2011; Kennerley et al., 2009; Kolling et al., 2012, 2016). Our current findings suggest greater activation of this network on high missing information trials when participants go on to choose the ambiguous option. Interestingly, the extent to which dACC and IFS activity on high missing information trials was elevated when the ambiguous option was chosen varied positively with trait anxiety. This could conceivably be explained by high trait anxious individuals requiring stronger activation of these frontal regions to overcome instinctual engagement of an ambiguity avoidance heuristic. Support for this interpretation comes from prior findings by De Martino et al. (2006). They reported that dACC showed increased activation when participants acted in opposition to framing effects engendering risk aversion or risk seeking. The authors contended that framing biases such as risk aversion might reflect application of an ‘emotional’ heuristic that at times is suboptimal and requires overriding for rationally optimal behavior. It is possible that this is also the case for ambiguity aversion and that anxious individuals might require increased engagement of dACC and other frontal regions to overcome their predisposition to ambiguity avoidance.

Given the similar activation pattern of dACC and IFS to levels of missing information, it is difficult in the context of the current study to determine if these regions play identical or complementary roles. Elsewhere, the IFS has been reported to respond strongly to the level of ambiguity even when no action can be taken (Bach et al., 2009). Bach and colleagues argued that the IFS might support information-directed exploration, with this region being activated even when such exploration is curtailed, for example when action is not allowed. In our study, the information missing was never revealed. However, the outcome of each trial when the ambiguous option was chosen could be used to assess outcome ‘surprise’ – that is how unexpected the outcome was given the urn contents. This could potentially aid the participant in improving their use of information level to conduct a Bayesian correction of outcome probability on future trials. Hence, one possibility is that, even in our task, IFS might be supporting information-directed exploration to some extent. Here, it is of note that on ambiguous trials where participants chose the ambiguous urn there was a significantly stronger response in IFS to outcome surprise than for those trials where participants chose the unambiguous urn (see Supplementary fMRI Analyses: outcome time analyses).

It has previously been reported that activity in both dACC and IFS increases as a function of task difficulty (Bishop et al., 2007; Shenhav et al., 2014, 2016). This raises the issue of whether high trait anxious individuals might find evaluation of urns on ambiguous trials more challenging than low trait anxious individuals. This might plausibly result in greater reliance on an ambiguity avoidance heuristic and increased activation of frontal regions on trials where the ambiguous urn is actually chosen. While it is hard to rule this possibility out completely, reaction time analyses show no relationship between trait anxiety and time taken to choose between urns as a function of the extent of missing information, or whether the ambiguous urn was selected (ps >.1), see Supplementary Analyses: Reaction time analyses. In addition, as missing information levels increase, participants in general, and high trait anxious participants in particular, tend to only chose ambiguous urns when there is a big difference in absolute expected value between the two urns; bigger differences in expected value tend to make choice easier and indeed we found larger absolute differences in expected value to be associated with shorter reaction times (see Supplementary Analyses: Reaction time analyses).

High trait anxious individuals also showed increased RLPFC activation as a function of missing information. However, this did not vary as a function of whether the ambiguous urn or the unambiguous urn was chosen. This might tentatively suggest a role for RLPFC in tracking as opposed to resolving the presence of second order uncertainty. Previous work has implicated the RLPFC in metacognition (Fleming et al., 2012) and reported increases in RLPFC activation as decision confidence reduces (De Martino et al., 2013). Difficulty in assessing second order uncertainty might well lead to reduced confidence in choice evaluation. In interpreting this finding, it is important to note that the positive relationship between trait anxiety and RLPFC activity as a function of missing information was the sole positive effect for RLPFC observed in our current study. The lack of group-level activity in this region in our whole brain analyses (see Supporting Analyses: fMRI analyses) also prevented us from conducting a confirmatory analysis using whole-brain activity to an orthogonal contrast to define this ROI. Hence, caution in interpretation is required. More generally, investigation of the relationship between outcome probability estimation under second order uncertainty and decision-confidence, and the modulation of this by anxiety, would be an interesting avenue for further study.

Whereas intolerance of uncertainty, in general, and ambiguity, in specific, has long been theorized to be a core feature of anxiety (Dugas et al., 1998; Freeston et al., 1994), more recently it has been suggested that elevated self-reported intolerance of uncertainty might be a transdiagnostic marker of Internalizing Psychopathology more broadly (Boswell et al., 2013; Carleton et al., 2012; Gentes & Ruscio, 2011). In the current study, we chose to focus on trait anxiety given the greater literature relating intolerance of uncertainty to anxiety than to depression and given the relative absence of trait measures of depression (many studies use neuroticism as a proxy but this is also linked to vulnerability to anxiety; Kendler et al., 2007). This is a limitation as we cannot address whether information-level dependent ambiguity aversion, and alterations in dACC and IFS engagement when selecting options characterized by high levels of missing information, is specific to anxiety or common to both anxiety and depression. In future work we hope to address this by using bifactor analysis to tease apart variance in item-level questionnaire responses linked to anxiety- and depression-specific latent factors versus that captured by a general or ‘common’ negative affect factor. Initial work using such bifactor models has shown that deficits in adapting learning to volatility – another form of second-order uncertainty – is common to both anxiety and depression and predominantly related to scores on the general negative affect factor (Gagne et al., 2020). It will be of interest to determine whether this also holds for information-level dependent ambiguity aversion and altered recruitment of frontal regions when engaging with high missing information options. This might be predicted to be the case based on by findings that elevated intolerance of uncertainty is characteristic of both patients with anxiety and depressive disorders (Boswell et al., 2013; Carleton et al., 2012; Gentes & Ruscio, 2011).

Another limitation of the current study was that we did not directly assess self-reported intolerance of uncertainty using the Intolerance of Uncertainty scale or other related measures. It would be of interest to establish if elevated intolerance of uncertainty is linked to categorical ambiguity aversion or information-level dependent ambiguity aversion. If the former is observed and not the latter, this might suggest that distress from having to handle the presence of uncertainty does not scale with the level of missing information. Alternatively, if intolerance of uncertainty is associated with information-level dependent ambiguity aversion, an interesting question will be whether this mediates the link between trait anxiety, or other measures of internalizing psychopathology and unwillingness to engage with options characterized by high levels of missing information.

A final area of potential importance for future investigation concerns the relationship between avoidance of options characterized by missing information and other avoidance behaviors seen in anxiety disorders and studied using fear conditioning paradigms. That this might be of interest is suggested by findings linking elevated self-reported prospective intolerance of uncertainty to increased frequency of avoidance responses during the instrumental learning phase of a fear conditioning task and higher resistance to extinction (Flores et al., 2018). Here, work examining the neural mechanisms underlying reversal learning, in fear conditioning, and its disruption in PTSD (Li et al., 2011) may also be of pertinence as this provides a paradigm where second-order uncertainty also varies across trials in a manner that can be captured using computational modeling.

To conclude, the findings reported here reveal that high trait anxious individuals show increased information-level dependent ambiguity aversion. Activation in dACC and IFS varied positively with level of missing information, especially on trials where participants overcame the bias to avoid ambiguous options. High trait anxious individuals showed greater differential activation of these regions on high missing-information trials when the ambiguous option was engaged with, versus avoided. This potentially reflects the need for greater activation of dACC and IFS to enable rational decision-making to win out given a strong predisposition to engage ambiguity avoidance heuristics. More broadly this work illustrates how modeling approaches can be used to computationally characterize behaviors such as ambiguity avoidance and to explore the neuro-cognitive mechanisms disrupted in high trait anxious individuals, who are at elevated risk of developing anxiety disorders. In turn, this will hopefully advance development of treatment targets for future intervention-oriented research.

## METHODS

### PARTICIPANTS

Participants comprised local residents and UC Berkeley students and staff. Recruitment was via flyers posted around campus and downtown Berkeley and via two websites maintained by the UC Berkeley Department of Psychology. The first website (‘RSVP’) enables local residents and UC staff and students to take part in research studies for a small honorarium. The second website (‘RPP’) enables UC students taking psychology courses to take part in research studies for course credit. Participants received $25 or 1 credit per hour (typically $50 or 2 credits in total) for taking part.

To be eligible, participants had to be right-handed and aged between 18 and 40 years of age. No participant was excluded as a function of sex or ethnicity. Exclusion criteria included current receipt of psychoactive medication or psychological therapy, neurological illness, or contraindications for MRI participation. Forty-one participants were recruited into the study. Participants’ data were excluded if (i) fMRI data for the full task was not successfully obtained; (ii) debriefing revealed that the participant had not understood the task, (iii) if participants showed excessive movement (multiple spikes, especially if in excess of 3mm) or (iv) participants’ behavioral data gave outliers (+−3SD) across multiple parameters and different models. In total, data from eight participants was excluded during the data-collection phase – two participants did not complete all task runs; there were projection issues for two participants; data or data log-files were lost during transfer for three participants and one participant did not fully understand the task (established in debriefing). At the data analysis stage, data from two further participants were excluded. One showed large movement spikes and debriefing indicated difficulty understanding the task, the second had multiple outlying beta values (>3SD from the mean) across models of interest. The final sample comprised thirty-one healthy volunteers (age range 18–38, M = 21.6, SD = 4.1, 21 females). Twenty-eight participants shared their ethnicity. Of these participants, 15 were Caucasian, 10 Asian and 3 of mixed race; in addition, two of the mixed-race participants and one of the Caucasian participants were Hispanic. All participants performed at over 90% accuracy on trials where there was an obviously superior option (unambiguous trials where both outcome probability and outcome magnitude supported picking the same option).

### PROCEDURE

The study was approved by the Institutional Review Board of the University of California, Berkeley and all participants gave written informed consent prior to taking part. Participants completed the study at the UC Berkeley Helen Wills Brain Imaging Centre.

#### Trait Anxiety assessment

Participants completed the Spielberger State-Trait Anxiety Inventory (STAI; Spielberger et al., 1983) at the beginning of the experimental session. Participants’ scores on the STAI trait subscale ranged from 20 to 53 (M = 36.7, SD = 9.6). This range of scores is similar to published norms – working adults aged 19 to 39: female M = 36.2, SD = 9.5; male M = 35.6, SD = 9.76; college students M = 38.3, SD = 9.2; female M = 40.4, SD = 10.2 (Spielberger et al., 1983). There is no set clinical cut off. A meta-analysis of patient data indicates a higher, but overlapping range of scores in patients with anxiety disorders (Social Anxiety Disorder: M = 51.3, 95% CI = 40.0 – 62.7, Generalized Anxiety Disorder: M = 54.5, 95% CI = 51.8 – 57.2, Panic Disorder: M = 49.4, 95% CI = 47.3 – 51.5, Cuijpers et al., 2016).

#### Task

We devised an Ellsberg style urn task in which participants made a series of choices between pairs of urns with the aim to minimize the amount of electrical stimulation they received. We chose to use electrical stimulation rather than reward-based outcomes as theoretical models of anxiety focus primarily on avoidance of aversive events. Electrical stimulation was delivered as trains of 2 ms pulses using a DS7AH constant current electrical stimulator (Digitimer). This was controlled by the stimulus presentation computer and connected to the volar surface of participants’ non-dominant forearm using a bipolar electrode.

Prior to task performance, a calibration procedure was conducted to set the levels of electrical stimulation for each participant so that subjective pain levels were equated across participants as closely as possible. This procedure is described fully by Browning et al. (2015). Throughout calibration, participants reported the pain intensity of the shock received using a 1–10 scale, on which 1 corresponded to “minimal pain”, 10 to the “worst possible pain” and 7 to the worst pain which the participant was willing to tolerate receiving for the sake of the study. We first identified the amplitude of a single 2 ms electrical pulse that corresponded to a subjective pain level of ‘1’. We then increased the number of 2 ms pulses delivered in a single burst until a subjective intensity of ‘7’ was reported. To form a mapping from subjective pain scores between 1–7 and the number of pulses delivered, participants subsequently received 14 additional bursts of stimulation, each containing a number of pulses randomly selected from the range between that equating to level ‘1’ and that equating to level ‘7’. The subjective ratings given to these stimulation bursts were used to fit a sigmoid curve describing the mapping between number of delivered pulses and subjective pain. This curve was used to transform shock outcome magnitude (on a 1–150 scale) to a stimulation burst of a given pulse number. Specifically, an outcome magnitude of ‘1’ would correspond to a single 2 ms pulse delivery, while a magnitude of ‘150’ would elicit delivery of the number of pulses required to produce a subjective pain rating of ‘7’. The sigmoidal fit was used to determine all other magnitude level to number of pulses conversions.

Following calibration, participants completed 20 practice trials to ensure task comprehension. Participants then performed 4 runs of 50 trials of the aversive decision-making task while fMRI data were acquired; details of fMRI acquisition and analysis are given below. Each trial consisted of a choice between two urns of 50 ‘tokens’ (see Figure 1). The ‘tokens’ were ‘X’s and ‘O’s, with the ratio set separately for each urn and each trial. A token was randomly selected from the chosen urn. The selection of an ‘O’ was associated with potential receipt of electric shock. The magnitude of the shock that might be received was shown as a number above each urn. This magnitude varied from 1 to 150. Each magnitude was converted to a burst of electrical stimulation of a given duration based on participants’ subjective pain ratings during calibration (see above). Trials were divided into blocks of five. On each trial in a given block, the outcome for that trial was stored and displayed in a summary histogram at the top of the screen to avoid working memory strain on participants. At the end of each block of five trials, the outcome of one of the five trials was randomly selected. If the outcome selected was an ‘O’, an electrical shock of the corresponding magnitude was delivered (see Supporting Figure 1).

The appearance of a question mark indicated that participants should select one of the two urns by button press (Figure 1b). Participants were instructed to consider both the probability that an ‘O’ would be drawn (based on the proportion of ‘O’s in each urn) and the magnitude of shock they might receive. On 50% of trials, participants had full information about the proportion of ‘O’s, as they could see all tokens in each urn. We refer to these trials as ‘unambiguous trials’ (UT). On the other 50% of trials one urn had missing information, as some tokens were hidden by ‘=’ signs (Figure 1a). We refer to these trials as ‘ambiguous trials’ (AT). Ambiguous and unambiguous trials were interleaved in a pseudo-randomized order. On ambiguous trials, the level of missing information was varied evenly across 8 levels (number of occluded tokens = 10, 30, 40, 45, 46, 47, 48 or 49 out of 50). Participants were instructed that the tokens revealed in the ambiguous urn provided an estimate of the true proportion but with associated uncertainty. Specifically, we instructed participants that:

“What you can see is a random sample chosen from the urn. So in this case the k tokens you can see gives you some information about what’s in the urn, but you are also missing information. The proportion of ‘X’s to ‘O’s in what you can see might not exactly match the true proportion in the urn, just because you have only taken a small sample out to look at. The more you can see, the more information you have and the more likely it will be that the proportion in what you can see is closer to the true proportion of ‘X’s to ‘O’s in the urn”.

The proportion of ‘O’s (P) and magnitude of potential shock (M) for each urn was reset between trials, so no learning was required. This was explained to participants. The expected value (EV) difference (*EV*_2_ − *EV*_1_ = (*M*_2_*P*_2_ − *M*_1_*P*_1_)) for the urns was balanced (close to zero) on 2/3 of trials, to better elicit relative valuation of shock probability and magnitude while maintaining unpredictability. Outcome probabilities and magnitudes were balanced across missing information levels. For revealed tokens only, ‘X’s were repositioned to the left of the urn and ‘O’s to the right, to improve ease of estimation of the proportion of ‘O’s. We described this procedure to participants and explained that given only revealed tokens were re-positioned, knowing the identity of tokens in a partially occluded row provided no specific information as to the identity of other tokens in that row (i.e., samples were merely informative as to what might be pulled out of the urn in general).

### ANALYSIS OF BEHAVIORAL DATA

#### Model-free analysis of whether anxiety is linked to increased ambiguity avoidance as a function of level of missing information

Prior to computationally modeling participants’ choice behavior, we conducted a simple model-free analysis of the influence of extent of missing information upon choice. We examined the proportion of trials on which the unambiguous urn was chosen (i.e., the ambiguous urn avoided) as a function of missing information. As the difference in outcome probability and magnitude were both manipulated orthogonally to missing information level, an individual whose choices are not irrationally influenced by missing information would not show a changing proportion of occasions on which the unambiguous urn was selected as a function of missing information. Conversely, if the proportion of trials on which the unambiguous urn was selected increased (or decreased) with missing information level, this would indicate information-level dependent ambiguity aversion (or ambiguity seeking).

For each participant, we regressed the proportion of trials on which the unambiguous urn was selected onto missing information *A*:

$$P(U)=\beta_{0}+\text{sILDAA}*A$$

Here, “simple” information-level dependent ambiguity aversion (sILDAA) is given by the slope of this regression function. We use the term sILDAA to distinguish this measure from the information-level dependent ambiguity aversion parameter estimated in our model-based analyses. To examine if elevated trait anxiety was linked to increasing ambiguity aversion as a function of missing information, we correlated sILDAA against participants’ STAI trait anxiety scores. sILDAA values and trait anxiety scores were both normally distributed; for consistency with our model-based analyses, where the key parameter ILDAA is not normally distributed across participants, we report Spearman as well as Pearson correlation coefficients.

A one-tailed test is used as we had the directional hypothesis that high trait anxious individuals would show increasing ambiguity aversion at high levels of missing information.

#### Model-based analysis of behavioral data

On any given trial, there are multiple variables that might influence participants’ choice behavior including the potential magnitude of shock linked to each urn, the ratio of revealed tokens in each urn, the presence or absence of ambiguity and, for ambiguous urns, the level of missing information. By modeling the influence of these parameters on participants’ choice and examining how this varies as a function of trait anxiety we can gain a better picture of the influence of trait anxiety upon decision-making under ambiguity.

We used model comparison to inform our parameterization of participants’ behavior on the task. We focused primarily on four alternate models. Additional models, considered for comprehensiveness, are presented in a Supplementary Modeling Note. The four main models differed in two important aspects. First, we examined two alternate parametrizations of choice behavior as a function of outcome probability and outcome magnitude. Here, we assessed whether participants’ choice behavior was better captured by including separate parameters for the influence upon choice of the relative probability of drawing an ‘O’ from each urn and the relative magnitude of shock associated with each urn or by assuming that participants calculate the expected utility (EU) of each option to hand, using a weighted product of outcome probability and outcome magnitude, before comparing options. Second, for each of these two classes of model, we compared the fit of baseline models against models that additionally captured categorical ambiguity avoidance or ambiguity seeking, i.e., preference for unambiguous urns over ambiguous urns, or vice versa, and information-level dependent ambiguity avoidance/seeking (ILDAA), i.e., avoidance/seeking of ambiguous urns as a function of the level of missing information. The four resultant models are described below.

#### Model 1: Additive, baseline


$$P(U)=\frac{1}{1+\text{exp}\left(-\left(\beta_{1}*M_{\text{diff }}+\beta_{2}*|\text{log}|P_{\text{diff }}\right)\right)}$$

There are two parameters fit here: β_1_, β_2_

On each ambiguous trial, P(U) is the event that the unambiguous urn is chosen. On unambiguous trials, P(U) is replaced with P(1), the event that Urn 1 is chosen. To avoid side biases, the left urn was labelled Urn 1 on 50% of unambiguous trials (selected randomly) and the right urn was labelled Urn 1 on the remaining 50% of unambiguous trials. *M_diff_* and |log|*P_diff_* are z scored across trials*. M_diff_* is the difference in potential outcome magnitude between urns. On ambiguous trials, *M_diff_* = M_a_ – M_u_ where M_a_ is the magnitude for the ambiguous urn and M_u_ the magnitude for the unambiguous urn. On unambiguous trials, *M_diff_* = (M_2_ – M_1_) where M_2_ is the magnitude for Urn 2 and M_1_ is the magnitude for Urn 1. β_1_ is estimated across both ambiguous and unambiguous trials. *P_diff_* is the difference in probability of drawing an ‘O’ between urns. We used the log modulus (|log|) of *P_diff_* to reduce the influence of extreme probability differences between urns. On ambiguous trials, *P_diff_* = P_a_ – P_u_; on unambiguous trials, *P_diff_* = P_2_ – P_1_. β_2_ is estimated across both ambiguous and unambiguous trials. P_u_, P_1_ and P_2_ are estimated as k/50, where k = number of ‘O’s shown. P_a_ is estimated by E(p), p~Beta (1+k, 1+n−k) where k =number of ‘O’s shown and n = the total number of tokens revealed. This estimation of P_a_ allows for the rational use of missing information to inform urn choice.

#### Model 2: Expected Utility, baseline


$$P(U)=\frac{1}{1+\text{exp}\left(-\left(\beta_{1}*\left(EU_{a}-EU_{u}\right)\right)\right)}\;\text{EU}={\text{M}}^{\lambda }*\text{P}$$

There are two parameters fit here: β_1_, *λ*

As in Model 1, on each ambiguous trial, P(U) is the event that the unambiguous urn is chosen. On unambiguous trials, P(U) is replaced with P(1), the event that Urn 1 is chosen, *EU_u_* is replaced by *EU*_1_ and *EU_a_* is replaced by *EU*_2_ (see Model 1 for further details on balancing of left and right urns between Urn 1 and Urn 2, respectively). M_a_, M_u_, M_1_, M_2_, P_a_, P_u_, P_1_ and P_2_ are as defined in Model 1. The expected utility (EU) is calculated for each urn, on each trial, as follows: EU = M^λ^ P. Both λ and β_1_ are estimated across both ambiguous and unambiguous trials. Expected utilities are not z-scored.

#### Model 3: Additive, baseline plus parameters allowing for additional influences of categorical ambiguity and level of missing information upon choice


$$P(U)=\frac{1}{1+\text{exp}\left(-\left(\beta_{0}*C+\beta_{1}*M_{\text{diff }}+\beta_{2}*|\text{log}|P_{\text{diff }}+\beta_{3}*A\right)\right)}$$

This model includes 4 parameters: β_0_, β_1_, β_2_, β_3_

Model 3 is the model selected following comparison of models 1–4; results using this model to fit task performance are reported in the main manuscript. Model 3 extends Model 1. As in Model 1, on each ambiguous trial, P(U) is the event that the unambiguous urn is chosen. On unambiguous trials, P(U) is replaced with P (1), the event that Urn 1 is chosen. M_a_, M_u_, M_1_, M_2_, P_a_, P_u_, P_1_ and P_2_, *M_diff_*, *P_diff_*, β_1_ and β_2_ are as described in Model 1. β_0_ allows for an additional influence of the categorical presence or absence (*C* = 1,0) of ambiguity (on unambiguous trials, β_0_ is 0 as *C* is 0). β_3_ allows for the influence of missing information *(A*) on choice where *A* = 1− √(n/50). *A* is set to 0 on unambiguous trials as there is no missing information; values of *A* are z scored across ambiguous trials.

#### Model 4: Expected Utility baseline plus parameters allowing for additional influences of categorical ambiguity and level of missing information upon choice


$$P(U)=\frac{1}{1+\text{exp}\left(-\left(\beta_{0}*C+\beta_{1}*\left(EU_{a}-EU_{u}\right)+\beta_{3}*A\right)\right)}$$

This model includes 4 parameters: β_0_, β_1_, *λ*, β_3_

Model 4 extends Model 2. As in Model 2, on each ambiguous trial, P(U) is the event that the unambiguous urn is chosen. On unambiguous trials, P(U) is replaced with P(1), the event that Urn 1 is chosen. EU_a_, EU_u_, EU_1_, EU_2_, β_1_ and *λ* are as described in Model 2. As in Model 3 above, β_0_ allows for an additional influence of the categorical presence or absence (*C* = 1,0) of ambiguity (on unambiguous trials, β_0_ is 0 as *C* is 0). β_3_ allows for the influence of missing information *(A*) on choice. *A* = 1− √(n/50). *A* is set to 0 on unambiguous trials as there is no missing information; values of *A* are z scored across ambiguous trials.

#### Model comparison

A maximum likelihood criterion was used to individually optimize the model parameters for each participant and each model. We approximated the Bayesian model evidence for each model by penalizing model log-likelihoods using the Bayes information criterion (BIC) and Akaike information criterion (AIC), Figure 3a, b. The BIC more strictly penalizes models with a higher number of parameters. We compared the fit of models 1 to 4 across participants by Bayesian model selection (BMS; Stephan et al., 2009). This uses hierarchical Bayesian inference to estimate the prevalence of each model at the population level and to statistically test whether any one model is more prevalent than the others. This approach can reveal if different groups of participants are best fit by different models. BMS requires an estimate of the log model evidence for each model for each participant; here, we used BIC penalized log-likelihood values (Stephan et al., 2009). We investigated which of models 1 to 4 had the highest population-level prevalence across all participants; that is the population-level estimate of the proportion of participants best fit by each model. We also determined which of models 1 to 4 had the highest protected exceedance probability across all participants; this is the probability that each model is the most prevalent at the population level, i.e., that it is the most likely to explain behavior on the task. In addition, we used a median split on participants’ STAI trait scores and reconducted model comparison within the two resultant participant sub-groups to determine if the same model provided the best fit to data from high and low anxious participants (Figure S3).

#### Parameter recovery and model identification analyses

Parameter recovery analyses were conducted for models 1 to 4. For each model, a range of possible values were selected for each parameter (0–10 for β_0_, β_1_, β_2_ and β_3_ and 0–2 for *λ*). The range for each parameter was chosen to encompass the estimated values of all participants across models 1–4. New parameter values were chosen randomly from across this range. This was repeated 100 times and each set of ‘ground truth’ parameter values used to generate a simulated dataset. The model in question was then fit to these 100 simulated datasets and parameter values were re-estimated or ‘recovered’. These recovered parameter values were correlated against their ground truth values.

We also conducted a model identification analysis – this reveals how often model comparison, using BIC-penalized log-likelihood values, correctly selects the model that was used to simulate the data (i.e., the true model). Here, we used the 100 simulated datasets generated for models 1 to 4, as described above. For each dataset, all four models were fitted and compared using BIC and the best fitting model (i.e., the model with the lowest BIC-penalized log-likelihood value) was determined. The proportion of times that each model was identified as the best-fitting model was assessed. Model identification performance was indexed by the frequency with which the model that was used to generate the data (i.e., the true model) was correctly selected as the best-fitting model.

#### Comparison of parameter estimates across participants

Model 3 (the winning model) was fitted to each participant’s data using a maximum likelihood criterion to optimize model parameters for each participant. Values for β_0_, β_1_, β_2_ and β_3_ were estimated for each participant. These parameter values were explored for normality. Results of the tests of normality are presented in Table S2. To test the hypothesis that elevated levels of anxiety would be associated with increased avoidance of ambiguity as a function of missing information, we conducted a one-tailed directional test as to whether there was a significant positive correlation between STAI trait anxiety scores and β3 parameter values (also referred to as information-level dependent ambiguity avoidance (ILDAA) scores.) β_3_ parameter values were not normally distributed, so a Spearman rank correlation was conducted. For completeness we also examined whether β_3_ parameter values differed from 0 at a group level (i.e., whether participants as a group showed increased avoidance of ambiguity as a function of missing information), investigated the correlations between STAI trait anxiety scores and β_0_, β_1_ and β_2_ parameter values, and examined if β_0_, β_1_ and β_2_ parameter values differed from 0 at a group level.

### fMRI DATA ACQUISITION

MRI data were acquired using a 3T Siemens TIM Trio scanner with a 32-channel coil. Functional scans were collected using a gradient echo planar sequence with repetition time (TR) = 2.25s, echo time (TE) = 34 ms, flip angle = 74, voxel size = 2.38 × 2.38, slice thickness = 3.0 mm (2.4 mm slice and 0.6 mm inter- slice gap), matrix size = 98×98, and field of view = 234 × 234 mm. For each participant, 29 axial slices were collected in descending order with a slice tilt of between 28 and 35 degrees to maximally cover frontal cortex. Data were acquired over four fMRI runs, each of approximately 15 minutes duration. Data from these runs were concatenated prior to analysis. Anatomical data were collected using a T1-weighted MP- RAGE sequence with the following parameters: voxel resolution 1×1×1 mm^3^, Echo Time (TE) = 2.98 ms, Inversion time (TI) = 900 ms, Repetition time (TR) = 2300 ms.

### fMRI ANALYSIS

Pre-processing was conducted using FSL (FMRIB Software Library, Version 6.00, www.fmrib.ox.ac.uk/fsl), following the Human Connectome Project standardized pre-processing pipeline. After conversion of the fMRI data from DICOM to NIFTI format, we conducted skull removal using FSL’s Brain Extraction Tool (BET; Smith, 2002). Subsequent preprocessing steps included motion correction (conducted using FMRIB’s linear image registration tool MCFLIRT; Jenkinson et al., 2002; Jenkinson & Smith, 2001), slice-timing correction, functional to structural registration (conducted using Boundary Based Registration; Greve & Fischl, 2009), and nonlinear structural to standard space (template) registration. Spatial smoothing was conducted using a Gaussian kernel of FWHM 6.0 mm and high pass temporal filtering using a 120s full-width cut off.

For each participant, fMRI data were analyzed in an event-related manner. Two general linear models (GLMs) were fit to the data in FSL. For the first, trials were divided into Unambiguous Trials (UT) and Ambiguous trials (AT). For the second, Ambiguous Trials were further divided into trials where participants went on to choose the ambiguous urn and trials where they went on to choose the unambiguous urn. This resulted in three trial types: Unambiguous trials (UT); ambiguous trials, ambiguous urn chosen (AC); and ambiguous trials, unambiguous urn chosen (UC). In both GLMs, we included the following regressors (stick functions of 0.5 seconds duration) for each trial type. First, regressors were included to represent onset of the following trial stages: ‘Urns Presented’, ‘Response Made’ and ‘Outcome Presented’. We also included a number of parametric regressors of interest. For each trial type, and for two time points, ‘Urns Presented’ and ‘Outcome Presented’, we included outcome magnitude difference (M_a_–M_u_ or M_2_–M_1_) and the log modulus of outcome probability difference (|log|(P_a_-P_u_), or |log|(P_2_-P_1_)). For all ambiguous trials, a parametric regressor indicating missing information level (*A*) was also included at both these time points. At the ‘Outcome Presented’ time for each trial type, we also included a binary outcome regressor (O = −1; X = 1); and parametric regressors indicating outcome magnitude, outcome surprise (as given by –log (P_outcome token_ (either X or O) drawn from the chosen urn)), and signed surprise (positive values correspond to a better than expected outcome, negative values correspond to a worse than expected outcome). Note, for trials where the ambiguous urn was selected, the beta-binomial corrected probability was used for P_outcome token_ as for P_a_. We also included regressors at each trial stage to indicate trials where no response was made. Finally, an additional regressor was yoked to the outcome delivery screen at the end of each block, and two further regressors at the same time-point specified if a shock was delivered and the corresponding shock magnitude (if delivered). These regressors were all convolved with the hemodynamic response function. All parametric regressors were normalized prior to convolution. The models were temporally filtered with the same high-pass filter applied to the fMRI time series. Confound regressors indicating volumes with outlying effects of motion for each participant (found using FslMotionOutliers) were included in the models together with regressors indicating that volumes belonged to a given run. The default settings for FslMotionOutliers were used; volumes were treated as outliers if they had a root mean squared intensity difference with the reference volume exceeding the 75th percentile plus 1.5 times the interquartile range. The number of volumes removed ranged from 45 to 151 (M = 100.4, SD = 34.1); this represented at most 10% of the data collected. The number of volumes removed did not correlate with trait anxiety (p > 0.2, Spearman, two-tailed).

#### Region of Interest (ROI) Definition

##### Dorsal Anterior Cingulate Cortex (dACC) ROI

Behrens and colleagues reported that dorsal anterior cingulate cortex (dACC) activity tracks trial-wise contingency volatility during a reward-based probabilistic learning task (Behrens et al., 2007). Subsequent work in our lab using an aversive version of this task (with electrical stimulation as outcomes) found volatility-related activity in a dorsal medial prefrontal region of interest that overlapped with the dACC activation cluster reported by Behrens. This ROI was originally defined in a study exploring the relationship between sub-dimensions of anxious affect and resting state functional connectivity (Bijsterbosch et al., 2014), and extends 10 mm anterior and 10 mm posterior from central coordinates [0 32 36] (Bijsterbosch et al., 2014).

##### Left and Right Inferior Frontal Sulcus ROIs

Spherical ROIs of diameter 8 mm were defined in standard space. The central co-ordinates [38, 16, 34] and [−38, 16, 34] were informed by activation peaks reported by Huettel et al. (2006) from an analysis correlating preference for ambiguity seeking with activity to ambiguous versus risky trials.

##### Left and Right Rostrolateral Prefrontal Cortex (RLPFC)

Spherical ROIs of diameter 8 mm were defined in standard space. Central co-ordinates [27, 50, 28] and [−27, 50, 28] were used. This was informed by work by Badre et al. (2012) who reported that those participants who used second-order uncertainty to guide their choice behavior showed significant responses to second-order uncertainty in right RLPFC, with peak activation at [27, 50, 28].

##### Alternate definition of ROIs

Results from a supplementary ROI analysis using an alternate method for defining ROIs (group-level contrasts orthogonal to the information-level contrast of interest) are provided in the Supplementary Information (see Supplementary Analyses: fMRI analyses). This supplementary analysis differed in the approach taken to ROI definition but used the same approach for analysis as described below.

#### Region of Interest (ROI) Analyses

Z-score maps from contrasts of interest for each participant were transformed to standard space using the combined nonlinear transformation warp from EPI space to standard space estimated in pre- processing (applywarp, FMRIB Software Library). The mean z-scores for each contrast of parameter estimates (COPE) of interest for each subject were extracted from each ROI and entered into group level analyses conducted using SPSS. T-tests were used to determine if activity associated with a given contrast, within a given ROI, differed significantly from zero. If assumptions of normality were violated, we additionally conducted non-parametric sign rank tests (see Table S2). Correlational analyses were used to examine the relationship between ROI activity and trait anxiety. Here, we used non- parametric correlational analyses (Spearman) due to the greater potential for outliers to impact the results of parametric versus non- parametric analyses. Spearman correlations were also used for the analyses examining the relationship between ROI activity and ILDAA activity reported in the Supplementary Information.

## Supplementary Material

TablesS1S2

SuppFigures

SuppModels

SuppAnalyses:RT

SuppAnalyses:fmri

## Data Availability

Behavioral data is available at https://osf.io/M6WCH/. Standard functions from Matlab and Python were used to perform the behavioral modeling. Models implemented using these functions are given in the main text. Code is available from EL or CG upon request. fMRI modeling was conducted in FSL (FMRIB Software Library, Version 6.00, www.fmrib.ox.ac.uk/fsl).

## Box 1 The selected model

Model 3.
$$P(U)=\frac{1}{1+\text{exp}\left(-\left(\beta_{0}*C+\beta_{1}*M_{\text{diff }}+\beta_{2}*|\text{log}|P_{\text{diff }}+\beta_{3}*A\right)\right)}$$

On each ambiguous trial, P(U) is the event that the unambiguous urn is chosen. On unambiguous trials, P(U) is replaced with P(1), the event that Urn 1 is chosen. To avoid side biases, the left urn was labelled Urn 1 on 50% of unambiguous trials, selected randomly, and the right urn was labelled Urn 1 on the remaining 50% of unambiguous trials. *M_diff_*, |log|*P_diff_* and *A* are z scored across trials*. M_diff_* is the difference in potential outcome magnitude between urns. On ambiguous trials, *M_diff_* = M_a_ – M_u_ where M_a_ is the magnitude for the ambiguous urn and M_u_ the magnitude for the unambiguous urn. On unambiguous trials, *M_diff_* = (M_2_ – M_1_) where M_2_ is the magnitude for Urn 2 and M1 is the magnitude for Urn 1. β_1_ is estimated across both ambiguous and unambiguous trials. |log|*P_diff_* is the log modulus of *P_diff_*, i.e., sign(P_diff_) × log(|P_diff_|+1), where *P_diff_* is the difference in probability of drawing an ‘O’ between urns. On ambiguous trials, *P_diff_* = P_a_ – P_u_; on unambiguous trials, *P_diff_* = P_2_ – P_1_. β2 is estimated across both ambiguous and unambiguous trials. P_u_, P_1_ and P_2_ are estimated as k/50, where k = number of ‘O’s shown. P_a_ is estimated by E(p), p~Beta(1+k, 1+n−k) where k = number of ‘O’s shown and n = the total number of tokens revealed. This estimation of P_a_ allows for the rational use of missing information to inform urn choice. Model comparison revealed that estimating P_a_ in this manner improved model fit (see the Supplementary Modeling Note). It also allows for simple comparison of a rational and non-rational model (Model 1 includes *P_diff_* and *M_diff_* terms calculated in this manner but sets β_0_ and β_3_ to 0; see Methods). *C* represents the categorical presence or absence of ambiguity (1,0). *A* represents missing information where *A* = 1− √(n/50). β_0_ and β_3_ are only estimated on ambiguous trials as *C* and *A* are set to 0 on unambiguous trials. β_0_ captures variance explained by a general, categorical, preference for the unambiguous urn over the ambiguous urn. β_3_ gives a measure of the influence of missing information on choice over and above the rational use of missing information captured by the beta-binomial correction of P_a_. Positive values of β_3_ indicate information-level dependent ambiguity aversion (ILDAA). Including an additional intercept for unambiguous trials did not improve model fit.

## References

[R1] Bach DR, Hulme O, Penny WD, & Dolan RJ (2011). The known unknowns: Neural representation of second-order uncertainty, and ambiguity. The Journal of Neuroscience: The Official Journal of the Society for Neuroscience, 31(13), 4811–4820. DOI: 10.1523/JNEUROSCI.1452-10.2011 21451019 PMC3166851

[R2] Bach DR, Seymour B, & Dolan RJ (2009). Neural activity associated with the passive prediction of ambiguity and risk for aversive events. The Journal of Neuroscience: The Official Journal of the Society for Neuroscience, 29(6), 1648–1656. DOI: 10.1523/JNEUROSCI.4578-08.2009 19211872 PMC2688025

[R3] Badre D, Doll BB, Long NM, & Frank MJ (2012). Rostrolateral prefrontal cortex and individual differences in uncertainty-driven exploration. Neuron, 73(3), 595–607. DOI: 10.1016/j.neuron.2011.12.025 22325209 PMC3285405

[R4] Behrens TEJ, Woolrich MW, Walton ME, & Rushworth MFS (2007). Learning the value of information in an uncertain world. Nature Neuroscience, 10(9), 1214–1221. DOI: 10.1038/nn1954 17676057

[R5] Bijsterbosch J, Smith S, Forster S, John OP, & Bishop SJ (2014). Resting State Correlates of Subdimensions of Anxious Affect. Journal of Cognitive Neuroscience, 26(4), 914–926. DOI: 10.1162/jocn_a_00512 24168223 PMC3960721

[R6] Birrell J, Meares K, Wilkinson A, & Freeston M (2011). Toward a definition of intolerance of uncertainty: A review of factor analytical studies of the Intolerance of Uncertainty Scale. Clinical Psychology Review, 31(7), 1198–1208. DOI: 10.1016/j.cpr.2011.07.009 21871853

[R7] Bishop SJ, & Gagne C (2018). Anxiety, Depression, and Decision Making: A Computational Perspective. Annual Review of Neuroscience, 41(1), 371–388. DOI: 10.1146/annurevneuro-080317-062007 29709209

[R8] Bishop SJ, Jenkins R, & Lawrence AD (2007). Neural Processing of Fearful Faces: Effects of Anxiety are Gated by Perceptual Capacity Limitations. Cerebral Cortex, 17(7), 1595–1603. DOI: 10.1093/cercor/bhl070 16956980

[R9] Boorman ED, Behrens TE, & Rushworth MF (2011). Counterfactual Choice and Learning in a Neural Network Centered on Human Lateral Frontopolar Cortex. PLOS Biology, 9(6), e1001093. DOI: 10.1371/journal.pbio.1001093 21738446 PMC3125157

[R10] Boswell JF, Thompson-Hollands J, Farchione TJ, & Barlow DH (2013). Intolerance of Uncertainty: A Common Factor in the Treatment of Emotional Disorders. Journal of Clinical Psychology, 69(6). DOI: 10.1002/jclp.21965 PMC3712497 23381685

[R11] Browning M, Behrens TE, Jocham G, O’Reilly JX, & Bishop SJ (2015). Anxious individuals have difficulty learning the causal statistics of aversive environments. Nature Neuroscience, 18(4), 590–596. DOI: 10.1038/nn.3961 25730669 PMC4644067

[R12] Camerer C, & Weber M (1992). Recent developments in modeling preferences: Uncertainty and ambiguity. Journal of Risk and Uncertainty, 5(4), 325–370. DOI: 10.1007/BF00122575

[R13] Carleton RN (2012). The intolerance of uncertainty construct in the context of anxiety disorders: Theoretical and practical perspectives. Expert Review of Neurotherapeutics, 12(8), 937–947. DOI: 10.1586/ern.12.82 23002938

[R14] Carleton RN, Mulvogue MK, Thibodeau MA, McCabe RE, Antony MM, & Asmundson GJG (2012). Increasingly certain about uncertainty: Intolerance of uncertainty across anxiety and depression. Journal of Anxiety Disorders, 26(3), 468–479. DOI: 10.1016/j.janxdis.2012.01.011 22366534

[R15] Carleton RN, Norton MAPJ, & Asmundson GJG (2007). Fearing the unknown: A short version of the Intolerance of Uncertainty Scale. Journal of Anxiety Disorders, 21(1), 105–117. DOI: 10.1016/j.janxdis.2006.03.014 16647833

[R16] Cuijpers P, Gentili C, Banos RM, Garcia-Campayo J, Botella C, & Cristea IA (2016). Relative effects of cognitive and behavioral therapies on generalized anxiety disorder, social anxiety disorder and panic disorder: A meta-analysis. Journal of Anxiety Disorders, 43, 79–89. DOI: 10.1016/j.janxdis.2016.09.003 27637075

[R17] De Berker AO, Kurth-Nelson Z, Rutledge RB, Bestmann S, & Dolan RJ (2019). Computing Value from Quality and Quantity in Human Decision-Making. The Journal of Neuroscience, 39(1), 163–176. DOI: 10.1523/JNEUROSCI.0706-18.2018 30455186 PMC6325261

[R18] De Martino B, Kumaran D, Seymour B, & Dolan RJ (2006). Frames, Biases, and Rational Decision-Making in the Human Brain. Science, 313(5787), 684–687. DOI: 10.1126/science.1128356 16888142 PMC2631940

[R19] De Martino B, Fleming SM, Garrett N, & Dolan RJ (2013). Confidence in value-based choice. Nature Neuroscience, 16(1), 105–110. DOI: 10.1038/nn.3279 23222911 PMC3786394

[R20] Dugas MJ, Gagnon F, Ladouceur R, & Freeston MH (1998). Generalized anxiety disorder: A preliminary test of a conceptual model. Behaviour Research and Therapy, 36(2), 215–226. DOI: 10.1016/S0005-7967(97)00070-3 9613027

[R21] Ellsberg D (1961). Risk, Ambiguity, and the Savage Axioms. The Quarterly Journal of Economics, 75(4), 643–669. DOI: 10.2307/1884324

[R22] Fleming SM, Huijgen J, & Dolan RJ (2012). Prefrontal contributions to metacognition in perceptual decision making. The Journal of Neuroscience: The Official Journal of the Society for Neuroscience, 32(18), 6117–6125. DOI: 10.1523/JNEUROSCI.6489-11.2012 22553018 PMC3359781

[R23] Flores A, López FJ, Vervliet B, & Cobos PL (2018). Intolerance of uncertainty as a vulnerability factor for excessive and inflexible avoidance behavior. Behaviour Research and Therapy, 104, 34–43. DOI: 10.1016/j.brat.2018.02.008 29524740

[R24] Freeston MH, Rhéaume J, Letarte H, Dugas MJ, & Ladouceur R (1994). Why do people worry? Personality and Individual Differences, 17(6), 791–802. DOI: 10.1016/0191-8869(94)90048-5

[R25] Frisch D, & Baron J (1988). Ambiguity and rationality. Journal of Behavioral Decision Making, 1(3), 149–157. DOI: 10.1002/bdm.3960010303

[R26] Furnham A, & Marks J (2013). Tolerance of Ambiguity: A Review of the Recent Literature. Psychology, 04(09), 717–728. DOI: 10.4236/psych.2013.49102

[R27] Gagne C, Zika O, Dayan P, & Bishop SJ (2020). Impaired adaptation of learning to contingency volatility in internalizing psychopathology. ELife, 9, e61387. DOI: 10.7554/eLife.61387 33350387 PMC7755392

[R28] Gentes EL, & Ruscio AM (2011). A meta-analysis of the relation of intolerance of uncertainty to symptoms of generalized anxiety disorder, major depressive disorder, and obsessive-compulsive disorder. Clinical Psychology Review, 31(6), 923–933. DOI: 10.1016/j.cpr.2011.05.001 21664339

[R29] Grenier S, Barrette A-M, & Ladouceur R (2005). Intolerance of Uncertainty and Intolerance of Ambiguity: Similarities and differences. Personality and Individual Differences, 39(3), 593–600. DOI: 10.1016/j.paid.2005.02.014

[R30] Greve DN, & Fischl B (2009). Accurate and robust brain image alignment using boundary-based registration. NeuroImage, 48(1), 63–72. DOI: 10.1016/j.neuroimage.2009.06.060 19573611 PMC2733527

[R31] Hartley CA, & Phelps EA (2012). Anxiety and Decision-Making. Biological Psychiatry, 72(2), 113–118. DOI: 10.1016/j.biopsych.2011.12.027 22325982 PMC3864559

[R32] Hsu M, Bhatt M, Adolphs R, Tranel D, & Camerer C (2006). Neural Systems Responding to Degrees of Uncertainty in Human Decision-Making. Science, 310, 1680–1683. DOI: 10.1126/science.1115327 16339445

[R33] Huettel SA, Stowe CJ, Gordon EM, Warner BT, & Platt ML (2006). Neural signatures of economic preferences for risk and ambiguity. Neuron, 49(5), 765–775. DOI: 10.1016/j.neuron.2006.01.024 16504951

[R34] Jenkinson M, Bannister P, Brady M, & Smith S (2002). Improved optimization for the robust and accurate linear registration and motion correction of brain images. NeuroImage, 17(2), 825–841. DOI: 10.1006/nimg.2002.1132 12377157

[R35] Jenkinson M, & Smith S (2001). A global optimisation method for robust affine registration of brain images. Medical Image Analysis, 5(2), 143–156. DOI: 10.1016/S1361-8415(01)00036-6 11516708

[R36] Kendler KS, Gardner CO, Gatz M, & Pedersen NL (2007). The sources of co-morbidity between major depression and generalized anxiety disorder in a Swedish national twin sample. Psychological Medicine, 37(3), 453–462. DOI: 10.1017/S0033291706009135 17121688

[R37] Kennerley SW, Dahmubed AF, Lara AH, & Wallis JD (2009). Neurons in the frontal lobe encode the value of multiple decision variables. Journal of Cognitive Neuroscience, 21(6), 1162–1178. DOI: 10.1162/jocn.2009.21100 18752411 PMC2715848

[R38] Kim S, Hwang J, Seo H, & Lee D (2009). Valuation of uncertain and delayed rewards in primate prefrontal cortex. Neural Networks, 22(3), 294–304. DOI: 10.1016/j.neunet.2009.03.010 19375276 PMC2693219

[R39] Kolling N, Behrens T, Wittmann M, & Rushworth M (2016). Multiple signals in anterior cingulate cortex. Current Opinion in Neurobiology, 37, 36–43. DOI: 10.1016/j.conb.2015.12.007 26774693 PMC4863523

[R40] Kolling N, Behrens TEJ, Mars RB, & Rushworth MFS (2012). Neural Mechanisms of Foraging. Science, 336(6077), 95–98. DOI: 10.1126/science.1216930 22491854 PMC3440844

[R41] Krain AL, Wilson AM, Arbuckle R, Castellanos FX, & Milham MP (2006). Distinct neural mechanisms of risk and ambiguity: A meta-analysis of decision-making. NeuroImage, 32(1), 477–484. DOI: 10.1016/j.neuroimage.2006.02.047 16632383

[R42] Levy I, Snell J, Nelson AJ, Rustichini A, & Glimcher PW (2010). Neural representation of subjective value under risk and ambiguity. Journal of Neurophysiology, 103(2), 1036–1047. DOI: 10.1152/jn.00853.2009 20032238

[R43] Li J, Schiller D, Schoenbaum G, Phelps EA, & Daw ND (2011). Differential roles of human striatum and amygdala in associative learning. Nature Neuroscience, 14(10), 1250–1252. DOI: 10.1038/nn.2904 21909088 PMC3268261

[R44] Rogers RD, Owen AM, Middleton HC, Williams EJ, Pickard JD, Sahakian BJ, & Robbins TW (1999). Choosing between Small, Likely Rewards and Large, Unlikely Rewards Activates Inferior and Orbital Prefrontal Cortex. Journal of Neuroscience, 19(20), 9029–9038. DOI: 10.1523/JNEUROSCI.19-20-09029.1999 10516320 PMC6782753

[R45] Rosen NO, Ivanova E, & Knäuper B (2014). Differentiating intolerance of uncertainty from three related but distinct constructs. Anxiety, Stress, & Coping, 27(1), 55–73. DOI: 10.1080/10615806.2013.815743 23849047

[R46] Shenhav A, Cohen JD, & Botvinick MM (2016). Dorsal anterior cingulate cortex and the value of control. Nature Neuroscience, 19(10), 1286–1291. DOI: 10.1038/nn.4384 27669989

[R47] Shenhav A, Straccia MA, Cohen JD, & Botvinick MM (2014). Anterior Cingulate Engagement in a Foraging Context Reflects Choice Difficulty, Not Foraging Value. Nature Neuroscience, 17(9), 1249–1254. DOI: 10.1038/nn.3771 25064851 PMC4156480

[R48] Smith SM (2002). Fast robust automated brain extraction. Human Brain Mapping, 17(3), 143–155. DOI: 10.1002/hbm.10062 12391568 PMC6871816

[R49] Spielberger CD, Gorsuch RL, Lushene R, & Vagg PR (1983). Manual for the state-trait anxiety inventory, 1983. Consulting Psychologists’ Press. DOI: 10.1037/t06496-000

[R50] Stephan KE, Penny WD, Daunizeau J, Moran RJ, & Friston KJ (2009). Bayesian model selection for group studies. NeuroImage, 46(4), 1004–1017. DOI: 10.1016/j.neuroimage.2009.03.025 19306932 PMC2703732

