# Supplementary material for: The Computational and Neural Substrates of Ambiguity Avoidance in Anxiety
Source: Comput Psychiatr. 2022 Feb 3;6(1):8–33. doi: 10.5334/cpsy.67 (PMC9223033; doi:10.5334/cpsy.67)
Supplement: Supplementary Tables. — Supplementary Tables s1 and s2. [file cpsy-6-1-67-s1.pdf]

Supplementary Table 1 and Supplementary Table 2

		β_0 (effect of categorical ambiguity, C=1 or 0)	β_1 (*Mdiff*)	β_2 (|log|*Pdiff*)	β_3 (effect of missing information level, *A*)
**All Participants**	**Mean**	1.85	2.49	3.34	0.692
	**Std**	1.31	1.23	1.37	0.641
**Low Trait Anxiety (median split)**	**Mean**	1.45	2.38	2.91	0.461
	**Std**	0.973	0.872	1.15	0.456
**High Trait Anxiety (median split)**	**Mean**	2.22	2.59	3.74	0.909
	**Std**	1.49	1.52	1.47	0.725

Supplementary Table 1: Means and standard deviations for betas from the main model (model 3).

The means and standard deviations for the betas from model 3 are presented for the whole participant sample, and for low and high trait anxiety participant subgroups created by a median split on STAI trait anxiety scores.

	T-stat (df=30)	P value of t-test (2-tailed, Bonferroni corrected for ROI data)	K-stat, Lilliefors test (test of normality)	P value, 2-tailed, Lilliefors test (test of normality)	Z-val, sign rank test	P value, sign rank test (2-tailed, Bonferroni corrected for ROI data)
**Behavioral Group Level Results**						
**Model 3**						
β_0 (C)	7.88	8.58E-09	0.19	0.01	4.84	1.30E-06
β_1 (*Mdiff*)	11.23	2.89E-12	0.16	0.04	4.86	1.17E-06
β_2 (|log|*Pdiff*)	13.56	2.49E-14	0.18	0.01	4.86	1.17E-06
β_3 (A)	6.01	1.35E-06	0.17	0.03	4.72	2.33E-06
**fMRI Group Level Results (ROI activity)**						
**Response to missing information on Ambiguous chosen trials**						
dACC	3.00	0.027	0.16	0.036	2.61	0.046
Left IFS	3.84	0.003	0.20	0.002	3.21	0.007

Supplementary Table 2: Test results for group-level behavioral and fMRI ROI analyses where there is evidence for a non-normal distribution of parameter values. T-tests are often robust to violations of normality and hence were used for our group-level behavioral and fMRI tests. Here, we report both the t-test results and non-parametric (sign rank) test results for those behavioral (top) and fMRI (bottom) analyses where assumptions of normality were violated. The k-statistic and p value for the Lilliefors test of normality are also provided. For the fMRI ROI analyses, a monte carlo approximation was used to calculate the p value for the Lilliefors test of normality, with a maximum monte carlo standard error of 0.01. We note that all the non-parametric analyses reported here replicate the findings of the associated parametric analyses reported in the main text.
