# Supplementary material for: The Computational and Neural Substrates of Ambiguity Avoidance in Anxiety
Source: Comput Psychiatr. 2022 Feb 3;6(1):8–33. doi: 10.5334/cpsy.67 (PMC9223033; doi:10.5334/cpsy.67)
Supplement: Supplementary Figures. — Supplementary Figures s1 to s14. [file cpsy-6-1-67-s2.pdf]

Supplementary Figures.

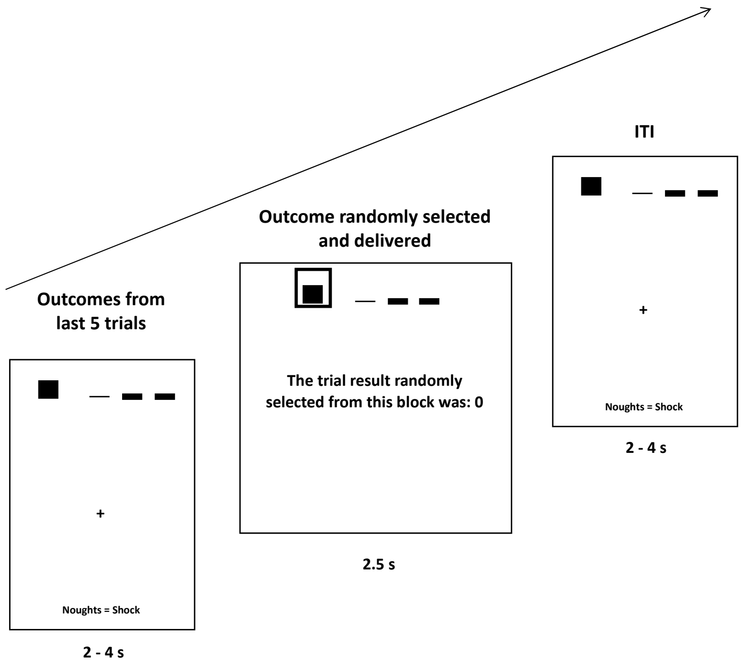

Supplementary Figure 1: Screens shown at the end of each block of 5 trials. After the fifth trial of each block, two additional intervals of 2-4s were added, one preceding and one following 'Outcome Selection and Delivery'. During these intervals, the outcomes from the prior five trials remained summarized in histogram form at the top of the screen. This histogram indicated the number of each of the following outcomes (from left to right): 'X', 'O' magnitude 1-50, 'O' magnitude 51-100, 'O' magnitude 101-150. This histogram was incremented at the end of each trial as described in **Figure 1**. At outcome selection and delivery, the outcome of one trial from the block was randomly selected, and displayed on the screen, both as the magnitude of the shock to be delivered and as a square around the corresponding summary histogram bar. If an 'X' outcome was selected, as in this example, the magnitude of shock delivered was 0 (no shock). If an 'O' outcome was selected, a shock of corresponding magnitude would be delivered. The post-outcome 2-4s intertrial interval (ITI) was added to reduce contamination of the BOLD response to the next trial from any delivered shocks. This preceded the standard 3-6s ITI before the next trial.

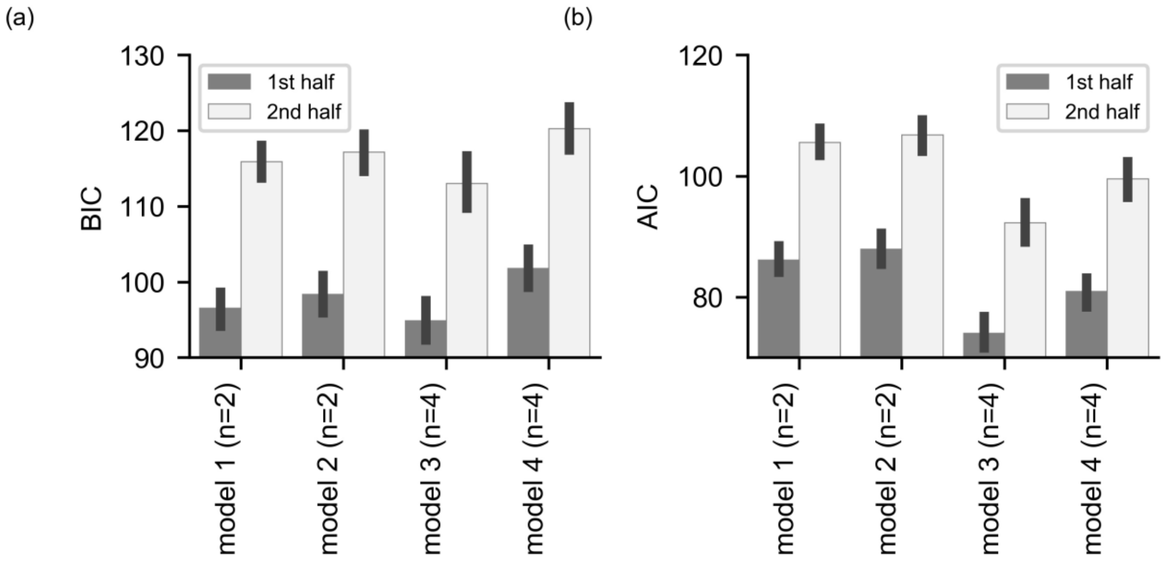

Supplementary Figure 2. Model fit for first and second half of task performance. To examine changes in model fit as participants became more familiar with the task, or more tired from participation, we divided trials into two sets: the first 100 (trials 1-100) and the second 100 (trials 101-200). We fit models 1 to 4 separately to each participant's choice data for each set of trials. The resulting BIC penalized log likelihoods (mean and standard deviation across participants) are given in panel (a) and the resulting AIC penalized log likelihoods (mean and standard deviation across participants) are given in panel (b). It can be seen that, for both sets of trials, model 3 performed best. All models showed better fits to data from the first half of the task; this likely reflects effects of fatigue during the second half of the task.

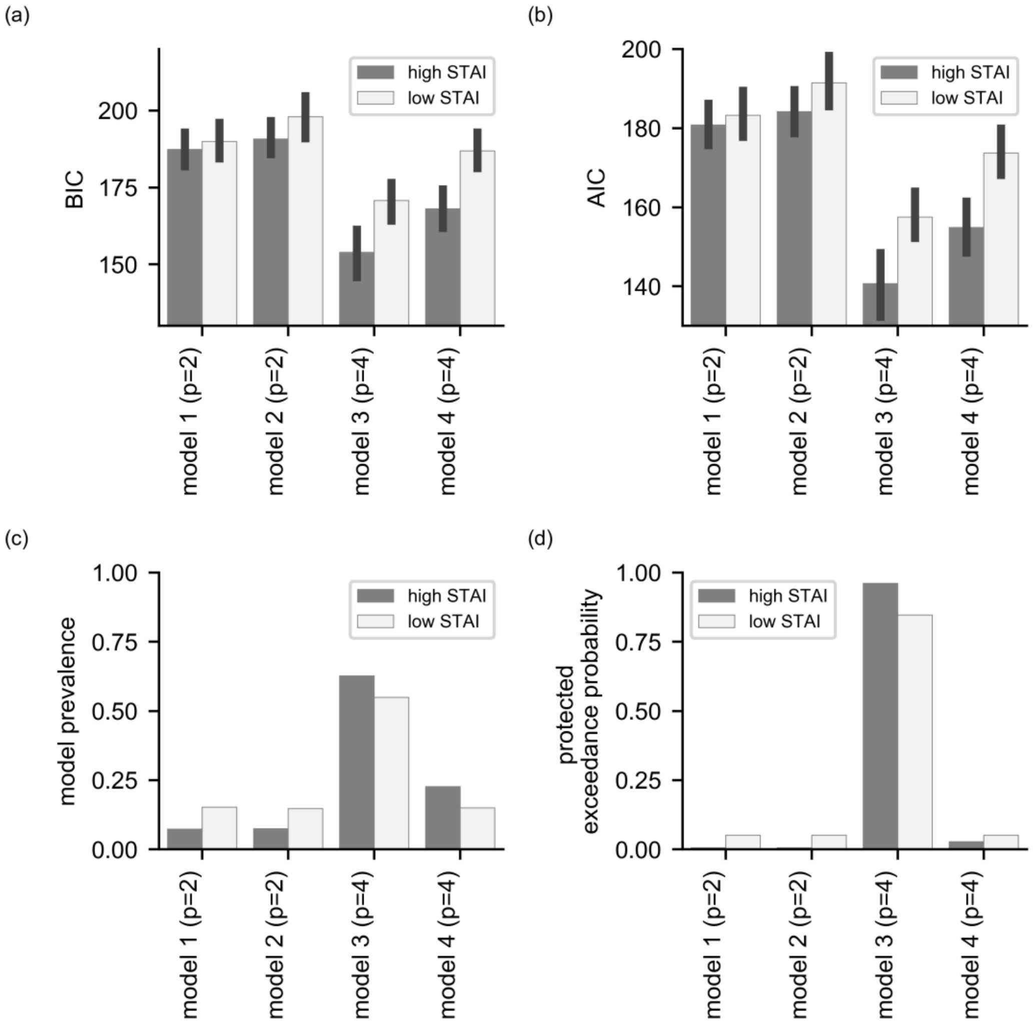

Supplementary Figure 3: Comparison of the four main models within low and high trait anxious subgroups. To explore model fit in low and high trait anxious participants, we used a median split on STAI trait scores and conducted model comparison separately for each of the two sub-groups created in this manner. In each panel of this figure, dark grey bars give model comparison results for individuals in the above-median, i.e. 'high' STAI-trait anxiety group; light grey bars give model comparison results for individuals in the below-median, i.e. 'low' STAI-trait anxiety group. The top row shows penalized log-likelihood values for each model averaged across participants. In panel (a), results shown are for model comparison using the Bayesian information criterion (BIC). Results from model comparison using the Akaike information criterion (AIC) are shown in panel (b). The bottom row shows the results from Bayesian model selection (BMS[22]). The BMS estimate of model prevalence, that is the population-level estimate of the proportion of participants best fit by each model, is given in panel (c). Panel (d) displays the protected exceedance probability for each model, this is the probability that each model is the most prevalent at the population level, i.e. the most likely to explain behavior on the task.

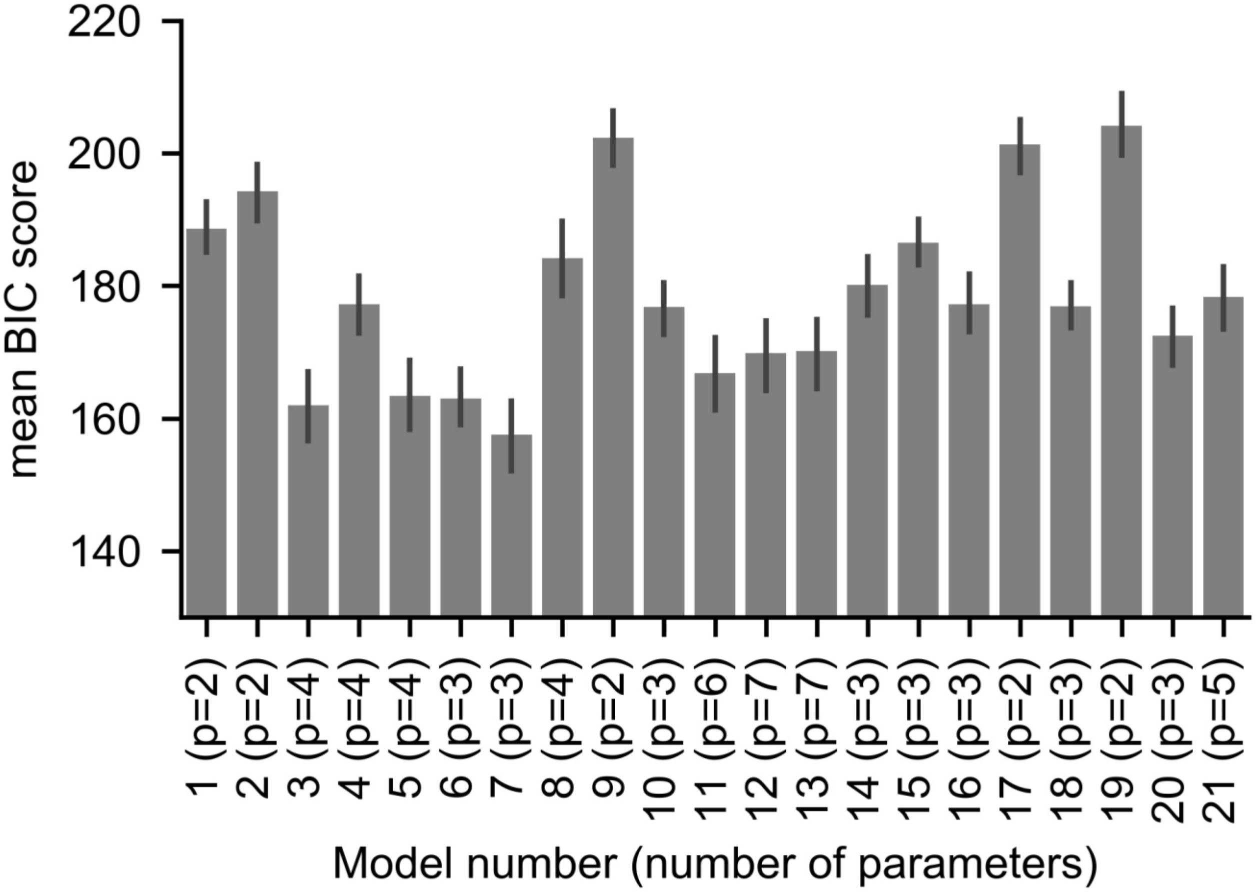

Supplementary Figure 4: BIC log likelihood estimates for all 21 models. The penalized log-likelihood values estimated using the Bayesian information criterion (BIC) and averaged across participants (error bars indicate standard deviation) are shown for the four main models described in the main text and **Methods** and for additional models 5 to 21 described in the **Supplementary Modeling Note.** For each model, the model number is given together with the number of parameters (p) in parentheses. Model 7 performs slightly but not significantly better than model 3; consideration of AIC results (shown in **Figure S4**) suggest that this largely reflects BIC's penalization of model 3's additional parameter. We chose to retain model 3 as our main model to enable us to control for categorical ambiguity while interrogating the effect of missing information upon choice over and above that captured by the rational beta-binomial correction of P_a.

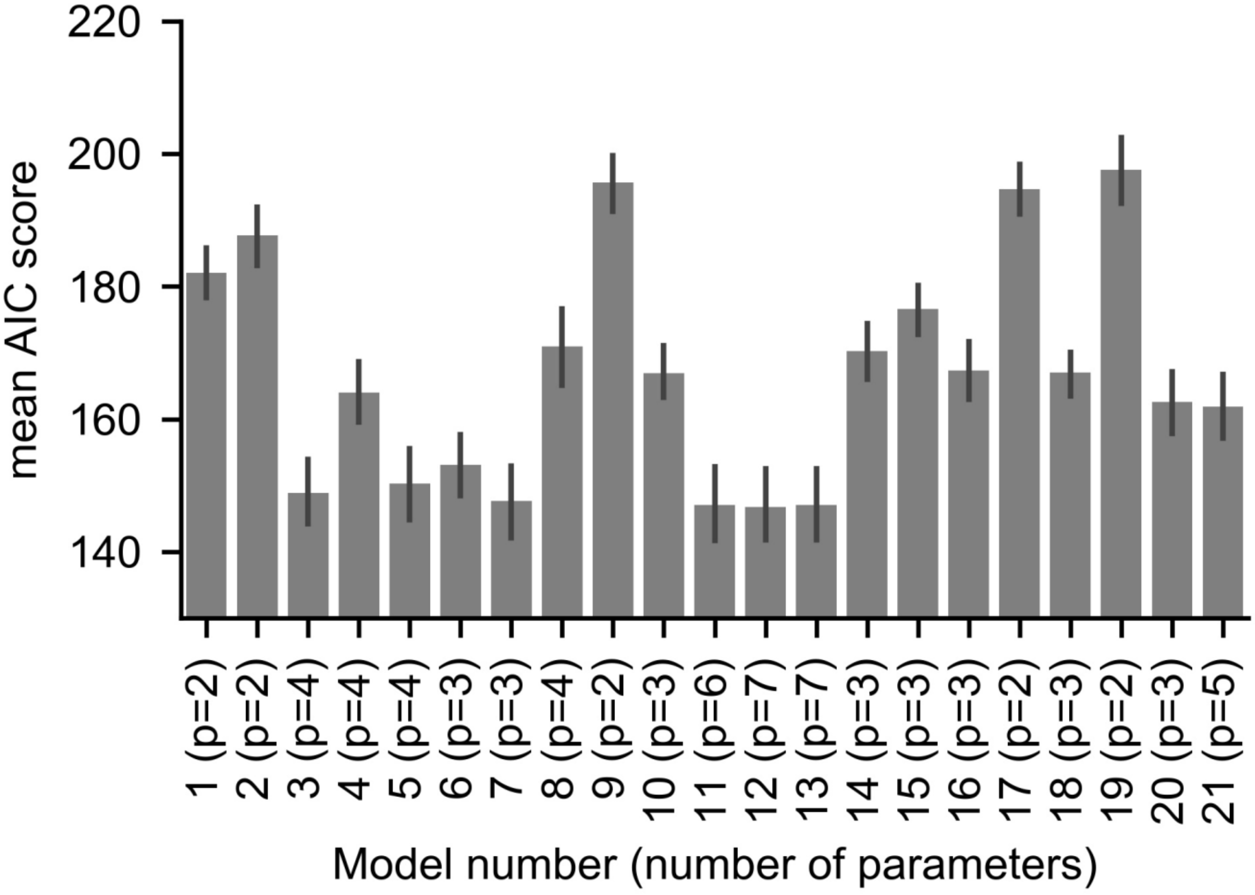

Supplementary Figure 5: AIC log likelihood estimates for all 21 models. The penalized log-likelihood values estimated using the Akaike information criterion (AIC) and averaged across participants (error bars indicate standard deviation) are shown for the four main models described in the main text and **Methods** and additional models 5 to 21 described in the **Supplementary Modeling Note.** For each model, the model number is given together with the number of parameters (p) in parentheses.

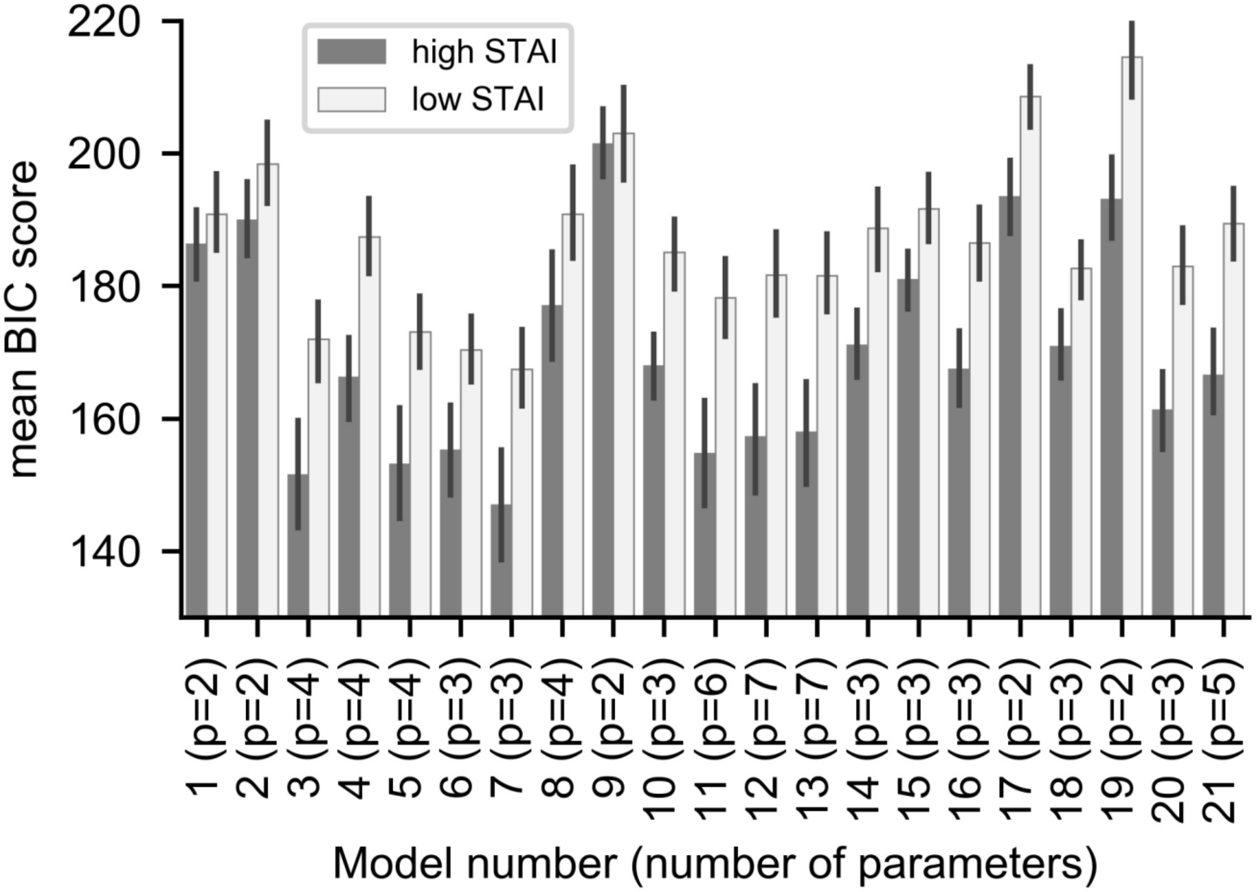

Supplementary Figure 6: BIC log likelihood results for all 21 models for participants grouped by a median split on STAI trait scores. The penalized log-likelihood values estimated using the Bayesian information criterion (BIC)) are shown for the four main models described in the main text and **Methods** and additional models 5 to 21 described in the **Supplementary Modeling Note.** For each model, the model number is given together with the number of parameters (p) in parentheses. We used a median split on STAI trait scores and averaged BIC scores across participants in each sub-group. Dark grey bars give mean BIC values for each model for the high (above-median) STAI trait anxiety group; light grey bars give mean BIC values for each model for the 'low' (below-median) STAI-trait anxiety group. Error bars represent standard deviation from the mean.

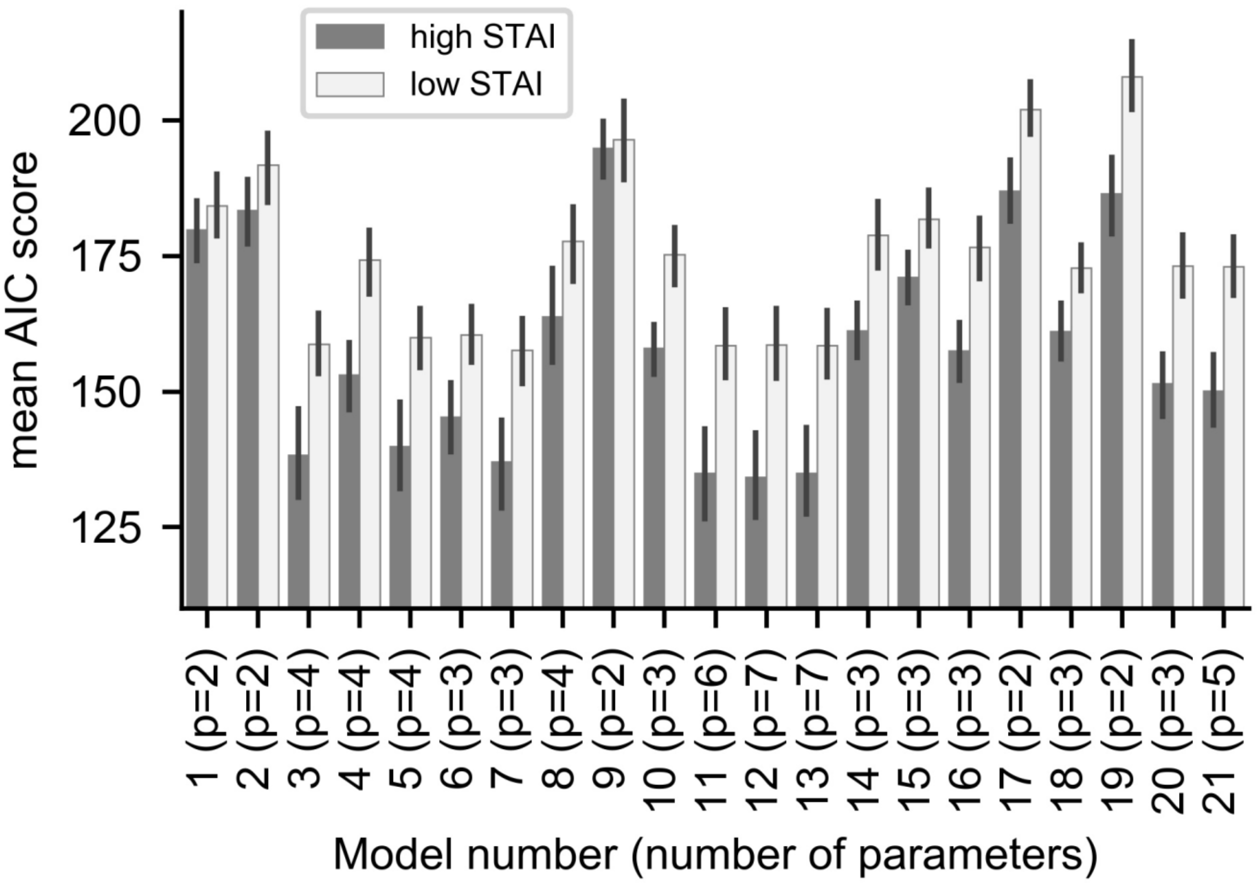

Supplementary Figure 7: AIC log likelihood results for all 21 models for participants grouped by a median split on STAI-trait scores. The penalized log-likelihood values estimated using the Akaike information criterion (AIC) are shown for the four main models described in the main text and **Methods** and additional models 5 to 21 described in the **Supplementary Modeling Note.** For each model, the model number is given together with the number of parameters (p) in parentheses We used a median split on STAI trait scores and averaged AIC scores across participants in each sub-group. Dark grey bars give mean AIC values for each model for the high (above-median) STAI trait anxiety group; light grey bars give mean AIC values for each model for the 'low' (below-median) STAI-trait anxiety group. Error bars represent standard deviation from the mean.

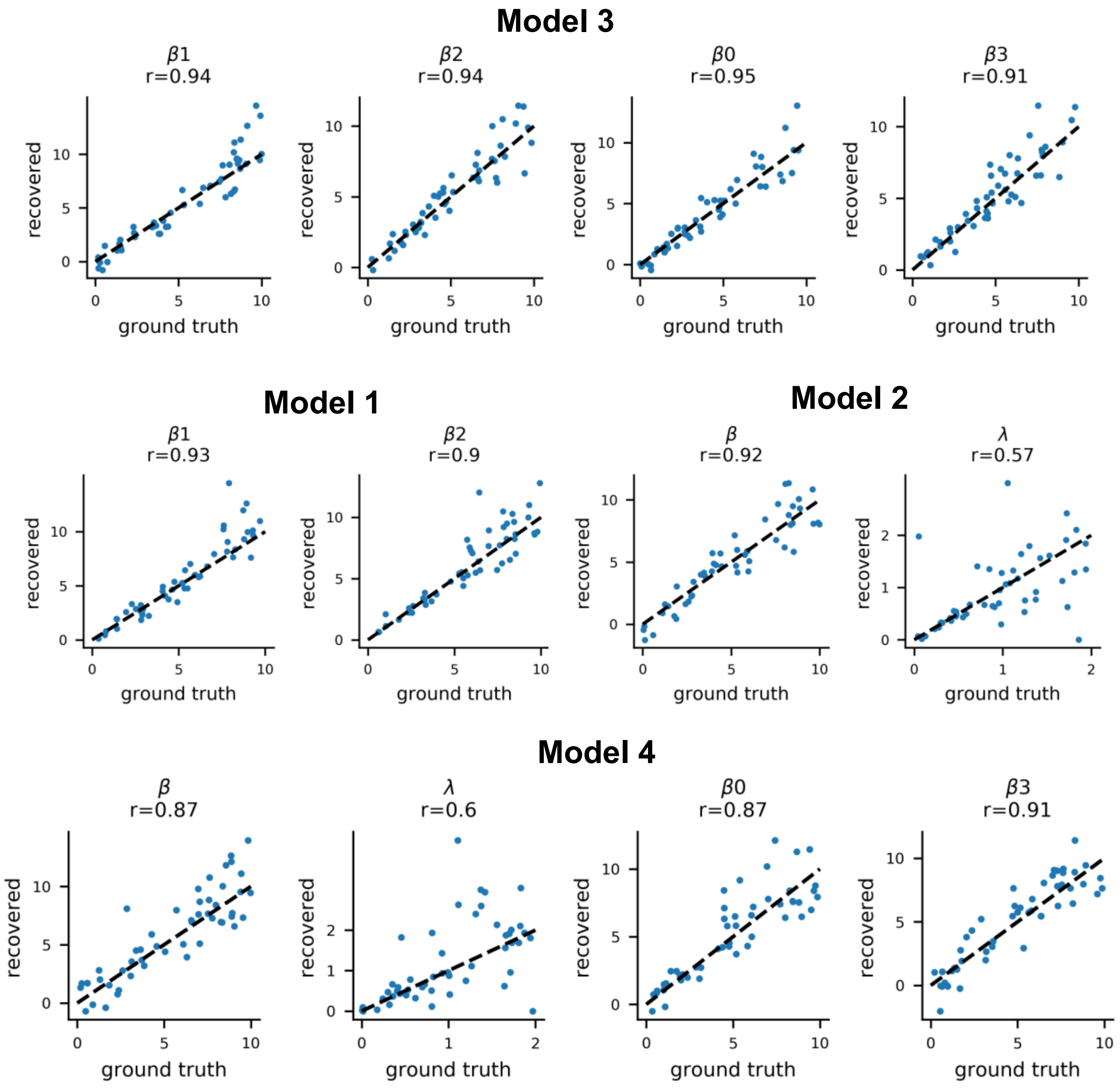

=

Supplementary Figure 8: Parameter recovery results.
Parameter recovery analyses were conducted for models 1 to 4. For each model, new parameter values were randomly selected from a range of possible values; this was repeated 100 times and each set of 'ground truth' parameter values were used to generate a simulated dataset (see the **Methods** for details). The model in question was then fit to these 100 simulated datasets and parameter values 'recovered' (i.e. re-estimated). Here, we plot recovered parameter values against the ground truth values. The results for model 3, the winning model, are shown in the top row; those for models 1 and 2 in the middle row, and those for model 4, in the bottom row. For the winning model, model 3, recovered parameter values showed strong correlations with ground truth parameter values, rs (98)>0.9, confirming that the model parameters were recoverable. This was also the case for model 1 (i.e. the base model version of model 3 omitting parameters for categorical and parametric ambiguity). For the two EU models, models 2 and 4, parameter recovery was moderate but not as good.

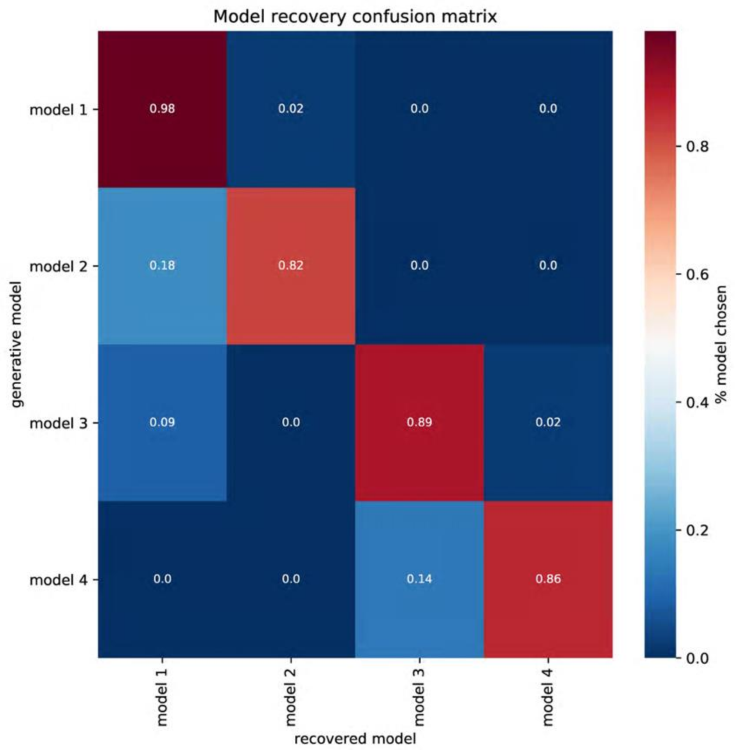

Supplementary Figure 9: Results of the model identification analysis. This reveals how often model comparison, using the Bayesian information criterion (BIC) as the comparison metric, correctly selects the model that was used to simulate a dataset. Models 1-4 and parameter values randomly selected from a range chosen to encompass all participants' estimated values were used to simulate 100 datasets (for details, see the **Methods**). All four models were fit to each dataset and the model with the lowest log-likelihood (i.e. lowest BIC score) was recorded as the model recovered. In this figure, we plot the proportion of times each model was selected or 'recovered' against the model used to generate the simulated dataset (the generative model). The winning model, model 3, was correctly identified 89% of the time when it was used to generate the data. Models 1, 2 and 4, were also correctly identified on over 80% of occasions. This analysis confirms that these four models make sufficiently distinct predictions to be distinguished from one another on the basis of participants' choice data.

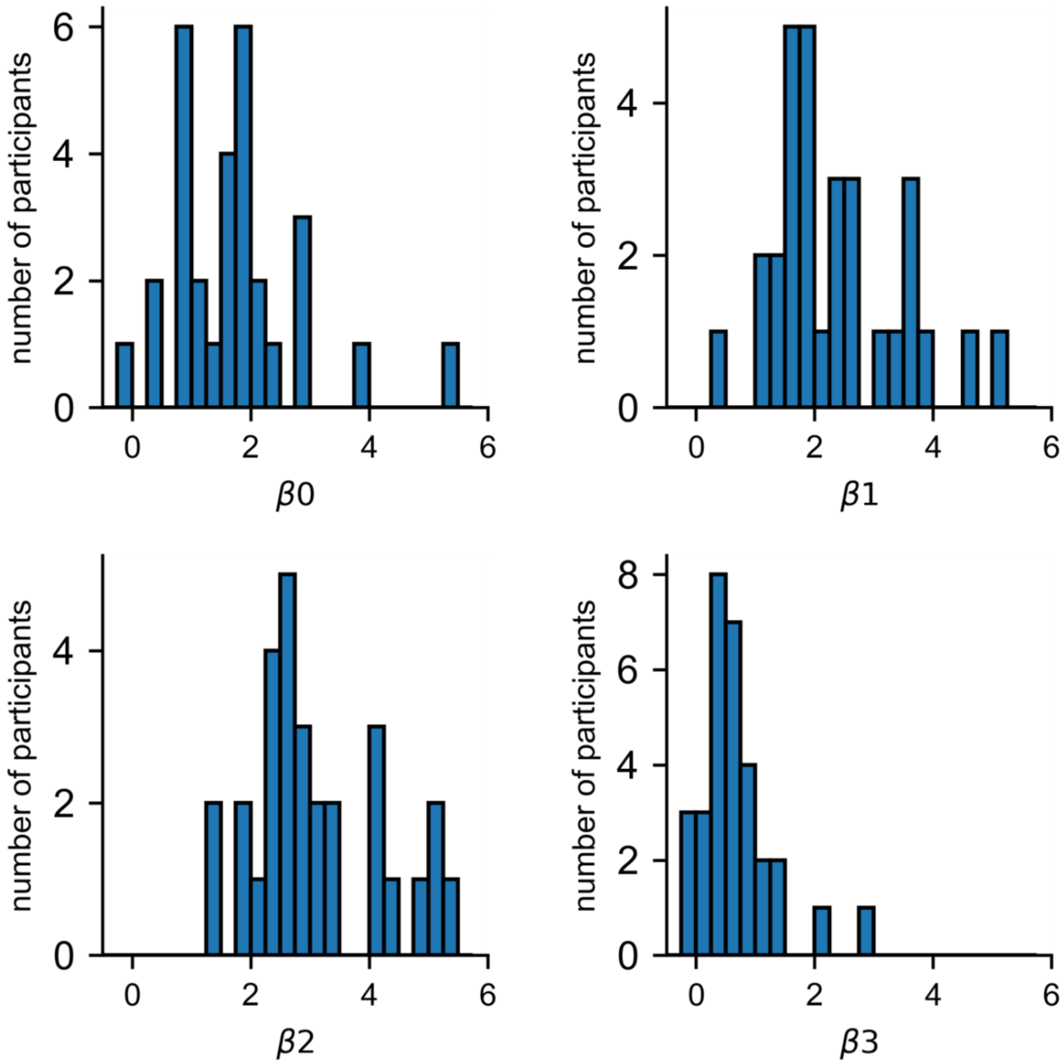

Supplementary Figure 10: Distribution of parameter estimates from the main model (model 3). Histograms showing the distribution of parameter estimates (beta0, beta1, beta2 and beta3) across all subjects, as obtained by fitting the experimental data using the main model, model 3.

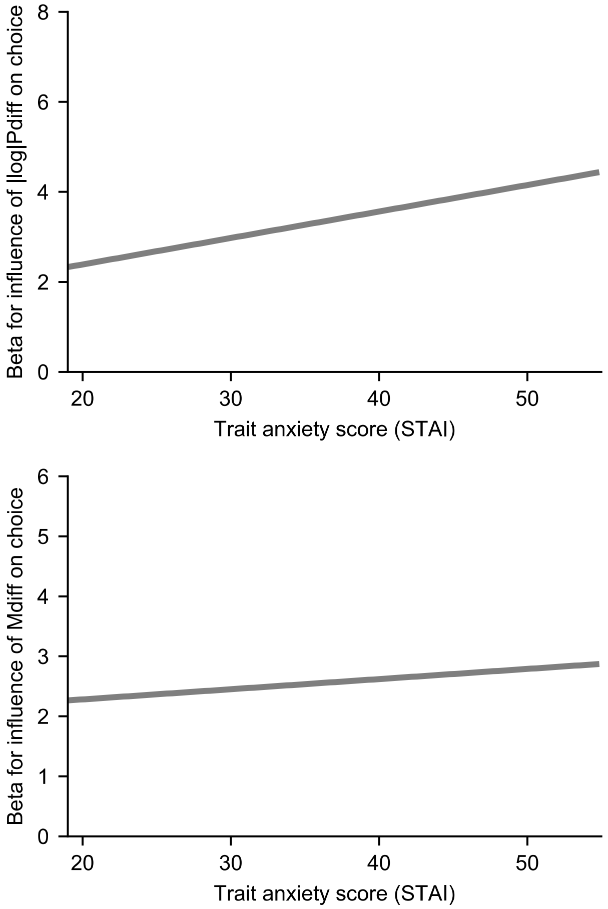

Supplementary Figure 11: Influence of outcome probability difference and outcome magnitude difference on urn choice as a function of trait anxiety. (a) Trait Anxiety, as assessed by scores on the trait subscale of the Spielberger State Trait Anxiety Inventory (STAI trait), was positively correlated with the extent to which difference in outcome probability between urns (|log|*Pdiff)* influenced choice behavior, $\beta_{2:}$ rho(29)=0.37, p=0.043 (Spearman, two-tailed). We note that we did not make a-priori predictions regarding the relationship between STAI trait scores and β_1 or β_2 values and this relationship would not survive correction for multiple comparisons across model parameters. (**b**) Trait anxiety was not correlated with the influence of difference in outcome magnitude (*Mdiff*) on choice behavior; $\beta_{1:}$ rho(29) = -0.0014, p=0.99 (Spearman, two-tailed). In both graphs, shaded regions represent +/- one standard error in the regression coefficients (obtained by resampling the data 10,000 times with replacement).

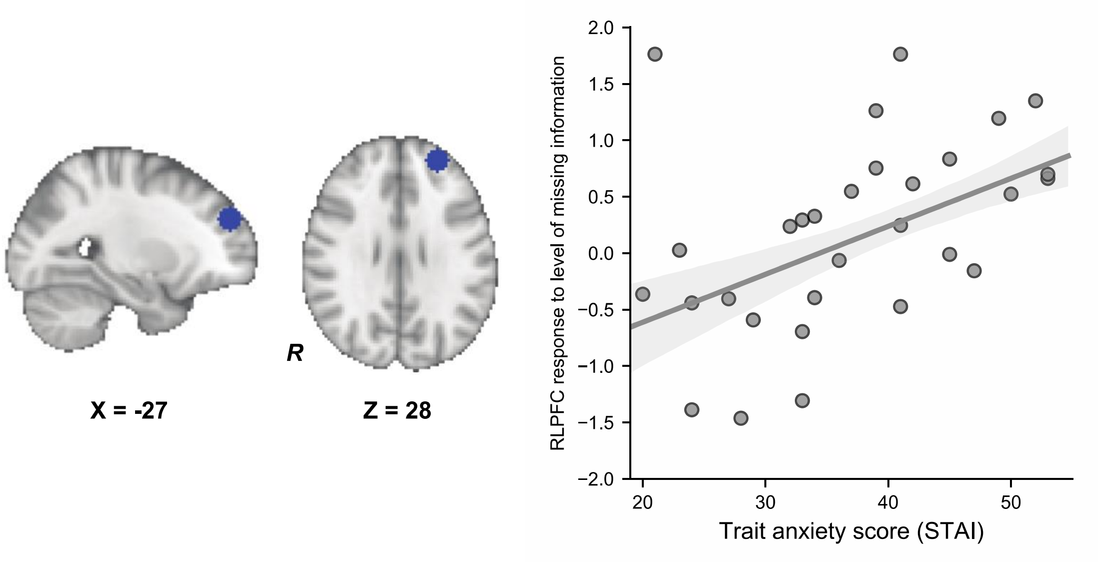

Supplementary Figure 12: Trait Anxiety was associated with heightened left RLPFC activity to missing information on ambiguous trials. Left: Sagittal and axial views of the left Rostrolateral Prefrontal Cortex ROI (RLPFC). This is an 8mm-radius spherical ROI centered at MNI co-ordinates -27, 50, 28 (see **Methods).** Radiological convention is used (L indicates left hemisphere). Right: The extent to which mean activation within the left RLPFC ROI, time-locked to urn presentation, varied as a function of level of missing information (*A*) on ambiguous trials was estimated for each participant (see **Methods**). The resulting z-score values are plotted against participant trait anxiety level. Trait anxiety was positively correlated with left RLPFC activation to missing information, r(29)=0.51, p=0.0038, p_{corr}=0.019, Spearman two-tailed. Note: Shaded regions represent +/- one standard error in the regression coefficients (obtained by resampling the data 10,000 times with replacement.) Additional analyses revealed that the relationship between trait anxiety and left RLPFC activity to missing information did not vary significantly as a function of urn chosen (p>0.2, Spearman, two-tailed).

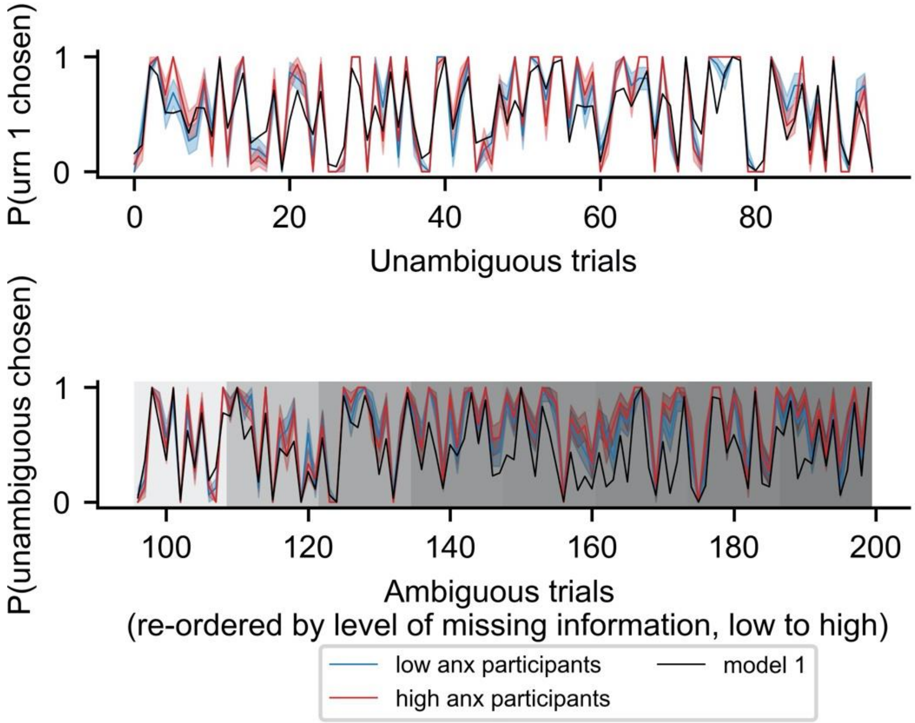

Supplementary Figure 13: Trial-wise choice behavior of high and low trait anxious participants as compared to baseline model 1. Participants are divided into high and low trait anxious groups using a median split on STAI trait scores. In the top panel, we plot the proportion of participants in each group that chose urn 1 on each unambiguous trial. Here, trials are numbered according to the order in which they were presented. In the bottom panel, we plot the proportion of participants in each group that chose the unambiguous urn on each ambiguous trial; here trials have been re-sorted by level of missing information with the lowest level (indicated by the lightest shade of grey) on the left and the highest level (indicated by the darkest shade of grey) on the right. In each panel, the sequential points corresponding to trials are joined to give a line plot. The data for the high trait anxious group is shown in red, the data for the low trait anxious group is shown in blue. The lighter colored shaded area round each line gives the standard error of the mean (standard deviation of participants' choices (0,1) divided by the square root of the sample size). To obtain predictions from model 1 on each trial, we applied model 1 to each participant's parameter estimates; this outputted an estimated probability (i.e. a number between 0 and 1) that the participant chose the unambiguous urn on that trial. This was repeated for all participants and the mean taken; the mean is plotted for each trial and the sequential points joined to give a line plot (in black). Model 1 applies a rational beta-binomial adjustment to estimates of outcome probability for the ambiguous urn. Parameters for the influence upon choice of outcome magnitude difference between urns and outcome probability difference between urns are estimated across all trials (ambiguous and unambiguous). It can be seen that deviations from model 1 become particularly noticeable at higher levels of missing information. When the blue and red lines are above the black model line on ambiguous trials, it means that participants are choosing the unambiguous urn (and avoiding the ambiguous urn) more than model 1 would predict; it can be seen that the red line tends to also be higher than the

blue line on these occasions, indicating that high trait anxious individuals are engaging in this behavior to a greater extent.

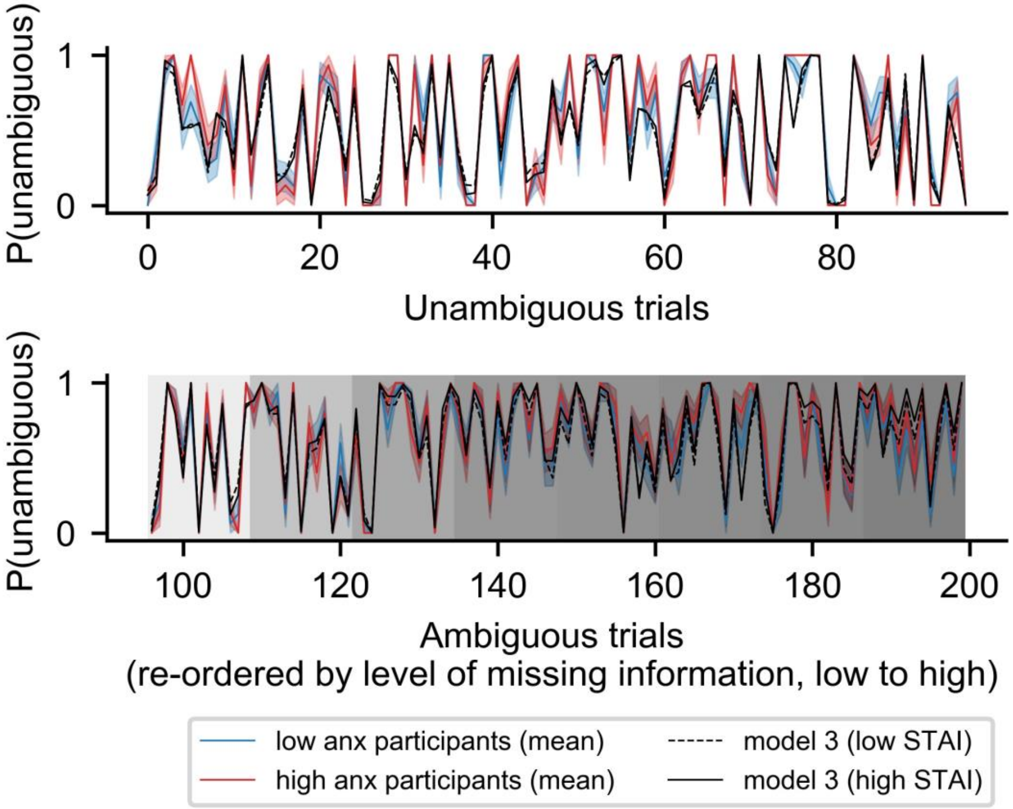

Supplementary Figure 14: Trial-wise choice behavior of high and low trait anxious participants as compared against model 3. Participants are divided into high and low trait anxious groups using a median split on STAI trait scores. In the top panel, we plot the proportion of participants in each group that chose urn 1 on each unambiguous trial; here trials are numbered according to the order in which they were presented. In the bottom panel, we plot the proportion of participants in each group that chose the unambiguous urn on each ambiguous trial; here trials have been re-sorted by level of missing information with the lowest level (indicated by the lightest shade of grey) on the left and the highest level (indicated by the darkest shade of grey) on the right. In each panel, the sequential points corresponding to trials are joined to give a line plot. The data for the high trait anxious group is shown in red, the data for the low trait anxious group is shown in blue. The lighter colored shaded area round each line gives the standard error of the mean (standard deviation of participants' choices (0,1) divided by the square root of the sample size). To obtain predictions from model 3, on each trial we applied model 3 to each participant's parameter estimates; this outputted an estimated probability (i.e. a number between 0 and 1) that the participant chose the unambiguous urn on that trial. This was repeated for all participants in each group (high STAI; low STAI) and the mean for each group estimated; the mean was plotted for each trial and the sequential points joined to give a line plot. The solid black line gives the model's choice prediction for the high STAI group and the dotted black line gives the model's choice prediction for the low STAI group. Model 3 is based on Model 1 but also includes additional parameters for the effect of categorical ambiguity and

missing information level on choice. Comparison of this figure and Figure S13 reveals that model 3 show a particular improvement, relative to model 1, in capturing participants' choice data on trials with high levels of missing information.
