# Supplementary material for: The Computational and Neural Substrates of Ambiguity Avoidance in Anxiety
Source: Comput Psychiatr. 2022 Feb 3;6(1):8–33. doi: 10.5334/cpsy.67 (PMC9223033; doi:10.5334/cpsy.67)
Supplement: Supplementary Modeling Note. — Here we present supplementary models 5–22. [file cpsy-6-1-67-s3.pdf]

Supplementary Modeling Note.

For description of the main model (Model 3) please see the main text and **Methods**. For Models 1, 2 and 4 please also see the **Methods**. For model comparison methods and results for models 1-4 please see the **Methods** and **Figure 3**. Here we present supplementary models 5-22. Mean log likelihoods for these models are given in Figures S3-S6.

<u>Model 5: Main model (Model 3), without log modulus.</u>

$$P(U) = \frac{1}{1 + \exp\left(-(\beta 0 * C + \beta 1 * Mdiff + \beta 2 * Pdiff + \beta 3 * A)\right)}$$

This model includes 4 parameters: $\beta_{0,}$ $\beta_{1,}$ $\beta_{2,}$ β_3

We used the log modulus to transform $Pdiff$ values in model 3 (and model 1) to reduce the influence of extreme probability differences. Here, we construct an alternate version of model 3 without the use of the log modulus. As in model 3, on each ambiguous trial, P(U) is the event that the unambiguous urn is chosen. On unambiguous trials, P(U) is replaced with P(1), the event that Urn 1 is chosen. To avoid side biases, the left urn was labelled Urn 1 on 50% of unambiguous trials (selected randomly) and the right urn was labelled Urn 1 on the remaining 50% of unambiguous trials. M_a, M_u, M_1, M_2, P_a, P_u, $P_{1,}$ P_2, *Mdiff* and *Pdiff* are as defined in Model 3. As in Model 3, β_0 allows for an influence of the categorical presence or absence (C = 1,0) of ambiguity (on unambiguous trials, β_0 is 0 as C is 0) whereas β_3 allows for the influence of missing information *(A*) on choice. $A = 1- \sqrt{(n/50)}$. A is 0 on unambiguous trials as there is no missing information; values of A are z scored across ambiguous trials.

Comparing mechanisms by which ambiguity may influence choice behavior: models 6-10.

In the models considered in the main manuscript (models 1-4), we account for the influence of ambiguity upon choice behavior in three ways. First, estimating the probability of pulling an 'O' out of the ambiguous urn (P_a) using a beta-binomial correction (E(p), p~Beta(1+k, 1+n-k), where k =number of 'O's shown and n = the total number of tokens revealed, allows for the rational adjustment of P_a to take into account the extent of missing information. As an example, if no information is missing, and there are 40 'O's and 10 'X's, P_a is 0.8. If 20 tokens are hidden and the ratio of revealed tokens is the same (i.e. 24 'O's and 6 'X's), P_a will be adjusted a little towards 0.5 due to the missing information (specifically, Pa = 0.78); if 45 tokens are hidden and the ratio of revealed tokens is the same (i.e. 4 'O's and 1 'X'), P_a will be adjusted towards 0.5 to a greater extent (specifically, Pa = 0.71). Over and above this rational adjustment of P_a, in models 3 and 4, we also include parameters that allow for an additional irrational influence of ambiguity on choice. Specifically, positive values of β_0 capture a general categorical preference for the unambiguous urn over the ambiguous urn while β_3 captures the extent to which avoidance of the ambiguous urn increases (or decreases) as a function of the level of missing information. Note, the beta-binomial correction of P_a will effectively mean that high P_a estimates (as calculated purely on the basis of the ratio of 'O's to 'X's) will be reduced when missing information is high but low estimates of P_a (as calculated purely on the basis of the ratio of 'O's to 'X's) will be increased under the same high levels of missing information. In contrast, β_3 captures an increasing tendency to avoid urns as a function of missing information that is irrespective of the ratio of 'O's to 'X's revealed.

In the models below, we examine the impact of removing the beta binomial correction in the calculation of P_a or removing the parameters capturing either categorical ambiguity

avoidance (β_0) or increased avoidance of the ambiguous urn as a function of missing information level (β_3).

Model 6: Removing the parameter capturing increased avoidance of the ambiguous urn as a function of missing information level (β_3).

$$P(U) = \frac{1}{1 + \exp\left(-(\beta 0 * C + \beta 1 * Mdiff + \beta 2 * |\log| Pdiff)\right)}$$

This model includes 3 parameters $\beta_{0,}$ $\beta_{1,}$ β_2

This model is identical to model 3 (that is, the winning model of models 1-4) except that the parameter allowing for an irrational influence of missing information level on choice (β_3* A) has been removed. Note, this model still includes the beta-binomial correction of P_a (as in model 3) and a parameter allowing for a difference in urn preference according to the categorical presence or absence of ambiguity (β_0). All other variables are as defined in model 3. As in model 3, on each ambiguous trial, P(U) is the event that the unambiguous urn is chosen. On unambiguous trials, P(U) is replaced with P(1), the event that Urn 1 is chosen.

Model 7: Removing the parameter capturing preference for the unambiguous urn over the ambiguous urn (effect of the categorical absence versus presence of ambiguity, β_0).

$$P(U) = \frac{1}{1 + \exp\left(-(\beta 1 * Mdiff + \beta 2 * |\log| Pdiff + \beta 3 * A)\right)}$$

This model includes 3 parameters: $\beta_1, \beta_2, \beta_3$

Model 7 is based on model 3. The parameter (β_0) allowing for a difference in preference for urns according to the categorical presence or absence of ambiguity (*C*= 1 or 0) has been removed. Note, this model still includes the beta-binomial correction of P_a (as in model 3). There is also still a parameter allowing for an additional influence of missing information level on choice (β_3* *A*); this captures avoidance of the ambiguous urn that increases as missing information increases. In this model, A is not z-scored; with β_0 omitted, this is necessary to distinguish unambiguous trials from ambiguous trials with an average level of ambiguity. Other variables are as defined in model 3. As in model 3, on each ambiguous trial, P(U) is the event that the unambiguous urn is chosen. On unambiguous trials, P(U) is replaced with P(1), the event that Urn 1 is chosen.

Model 8: No rational use of missing information to adjust Pa (probability of drawing an 'O' from the ambiguous urn).

$$P(U) = \frac{1}{1 + \exp\big(-(\beta 0 + \beta 1 * Mdiff + \beta 2 * |\log| Pdiff + \beta 3 * A)\big)}$$

This model includes 4 parameters: $\beta_0, \beta_1, \beta_2, \beta_3$. *Pdiff* on ambiguous trials = P_a - P_u where P_a= k/n

On each ambiguous trial, P(U) is the event that the unambiguous urn is chosen. On unambiguous trials, P(U) is replaced with P(1), the event that Urn 1 is chosen. In model 3, estimating the probability of drawing an 'O' out of the ambiguous urn (P_a) using a beta-binomial correction (E(p), p~Beta(1+k, 1+n-k) where k =number of 'O's shown and n = the total number

of tokens revealed) allows for the rational adjustment of P_a to take into account the extent of missing information. In model 8, we replace this by simply using the observed proportion of 'O's (k/n) to estimate P_a (as is the case for P_u, P_1 and P_2). In this model, ambiguity can still influence choice through avoidance (or seeking) of ambiguous urns in general (β_0), or increasing avoidance (or seeking) of ambiguous urns as a function of missing information level (β_3). These biases are irrational as they do not take into account outcome probability or magnitude (as specified in the Methods, difference in outcome magnitude and outcome probability were varied orthogonally with respect to both categorical ambiguity and level of missing information).

<u>Model 9: No accounting (rational or irrational) for influence of missing information on choice behavior</u>

$$P(U) = \frac{1}{1 + \exp\left(-(\beta 1 * Mdiff + \beta 2 * |\log|Pdiff)\right)}$$

This model includes 2 parameters: $\beta_{1,}$ β_2.

Pdiff on ambiguous trials = P_a - P_u where P_a= k/n; *Mdiff* = M_a-M_u.

On each ambiguous trial, P(U) is the event that the unambiguous urn is chosen. On unambiguous trials, P(U) is replaced with P(1), the event that Urn 1 is chosen; here, *Pdiff* = P_2 - P_1, *Mdiff* = M_2 - M_1. In this model, we remove all influences of ambiguity on choice from model 3. P_a is calculated using k/n as for P_u, P_1 and P_2, where k is the number of 'O's revealed and n is the total number of tokens revealed. The parameters allowing for an influence of categorical ambiguity (β_0) and level of missing information upon choice (β_3) are also both removed. Note, this model differs from model 1 in that model 1 allows missing information to rationally

influence choice through use of the beta-binomial correction to estimate P_a. All variables not otherwise specified are as defined in model 3 (and model 1).

Model 10: Expected Utility model with additive ambiguity parameter (accounting for categorical ambiguity aversion).

$$P(U) = \frac{1}{1 + \exp\left(-\left(\beta 0 * C + \beta 1 * (EU_a - EU_u)\right)\right)}$$

$EU = M^{\lambda} * P$

This model includes 3 parameters: $\beta_0, \beta_1, \lambda$

The expected utility models perform more poorly as a class (as previously discussed in relation to models 2 and 4). However, we include model 10 for comparability with other work in the literature that has explored decision-making under ambiguity in healthy adults[10]. This model is based on Model 2 but includes a parameter (β_0) to allow for the influence of the categorical presence or absence of ambiguity (*C*=1 or 0) upon urn choice. On each ambiguous trial, P(U) is the event that the unambiguous urn is chosen. On unambiguous trials, P(U) is replaced with P(1), the event that Urn 1 is chosen. On these trials, EU_a is replaced by EU_2, and EU_u is replaced by EU_1. Variables not specified here are as defined in Model 2. Missing information informs the estimate of P_a via the beta-binomial correction (as detailed in model 1). Unlike model 4, no additional influence of missing information on choice is included (i.e. $\beta_3 * A$ is excluded).

Section 3. Exploring the influence of ambiguity on use of probabilities and magnitudes: models 11-16.

In the models considered so far, we have modeled the influence of missing information upon participants' choice through applying a beta-binomial correction in the estimation of P_a and

through parameters (β_0, β_3) that allow the presence and level of missing information (*C, A* respectively) to have an additive effect on participants' choice (in addition to outcome probability and magnitude). In the models below, we explore whether the presence or level of missing information influences the weighting given to probability or magnitude information, such that these variables differentially influence behavior on unambiguous and ambiguous trials.

Model 11: Model 3 is extended to allow the influence upon choice of the difference in outcome probability between urns and that of the difference in outcome magnitude between urns to vary between ambiguous and unambiguous trials.

$$P(U) = \frac{1}{1 + \exp\left(-(\beta 0 * C + \beta 1_a * Mdiff + \beta 2_a * |\log|Pdiff + \beta 3 * A)\right)}$$

$$P(1) = \frac{1}{1 + \exp\left(-(\beta 1_u * Mdiff + \beta 2_u * |\log|Pdiff)\right)}$$

This model includes 6 parameters: $\beta 0, \beta 1_a, \beta 2_a, \beta 1_u, \beta 2_u, \beta 3$.

Note *C* and *A* are 0 on unambiguous trials so fall out of the equation for P(1).

On each ambiguous trial, P(U) is the event that the unambiguous urn is chosen. On unambiguous trials, P(U) is replaced with P(1), the event that Urn 1 is chosen. Model 11 is identical to model 3 except that the influence of magnitude difference and probability differences on choice is allowed to vary between ambiguous and unambiguous trials. See Model 3 for definition of variables.

Model 12: Model 11 with additional intercept ($\beta 0u$) paramater for unambiguous trials.

$$P(U) = \frac{1}{1 + \exp\left(-(\beta 0_a + \beta 1_a * Mdiff + \beta 2_a * |\log|Pdiff + \beta 3 * A)\right)}$$

$$P(1) = \frac{1}{1 + \exp\left(-(\beta 0_u + \beta 1_u * Mdiff + \beta 2_u * |\log|Pdiff)\right)}$$

This model includes 7 parameters: $\beta 0_a, \beta 1_a, \beta 2_a, \beta 3, \beta 0_u, \beta 1_u, \beta 2_u$

In this model we include a separate intercept for unambiguous trials ($\beta 0_u$). Effectively, ambiguous and unambiguous trials are modelled separately by P(U) and P(1) respectively. Given left and right urns are randomly allocated to be Urn 1 or Urn 2 on unambiguous trials we do not expect inclusion of this additional parameter to improve model fit relative to model 11.

Model 13: Model 11 with an additional parameter allowing an interactive effect of the probability of drawing an 'O' from the ambiguous urn and missing information level.

$$P(U) = \frac{1}{1 + \exp\left(-(\beta 0 * C + \beta 1_a * Mdiff + \beta 2_a * |\log|Pdiff + \beta 3 * A + \beta 4 * A * P_a)\right)}$$

$$P(1) = \frac{1}{1 + \exp\left(-(\beta 1_u * Mdiff + \beta 2_u * |\log|Pdiff)\right)}$$

This model includes 7 parameters: $\beta 0, \beta 1_a, \beta 2_a, \beta 1_u, \beta 2_u, \beta 3, \beta 4$

This model uses Model 11 as its base (Model 11 was selected over Model 12 due to its lower mean BIC score, see **Figure S4**) and additionally includes a free parameter for the effect on choice of the interaction between A and P_a. We modelled this interaction term to capture potential differences in the use of outcome probability to inform choice at different levels of missing information. We note this term has a different impact to the beta-binomial correction

used to estimate P_a as the beta-binomial correction upwards corrects low probabilities and downward corrects high probabilities at high levels of missing information.

Model 14: Expected Utility model with multiplicative ambiguity factor.

$$P(U) = \frac{1}{1 + \exp\left(-\left(\beta 1 * (EU_a - EU_u)\right)\right)}$$

For ambiguous urns: $EU_a = m * (M_a^{\lambda}) * P_a;$ for all other urns: $EU = M^{\lambda} * P$

Variables not specified are as defined in Model 2.

This model includes 3 parameters: β_1, λ, m

m boundaries are -25, 25

Model 14 is a second EU model included for comparability with other work in the literature that has explored decision-making under ambiguity in healthy adults[10]. In this model, the categorical presence of ambiguity influences the expected utility difference between the ambiguous and unambiguous urn by exaggerating or reducing the expected utility of the ambiguous urn by the multiplicative factor m. This allows the slope of the expected utility function to differ between ambiguous and unambiguous urns. On each ambiguous trial, P(U) is the event that the unambiguous urn is chosen. On unambiguous trials, P(U) is replaced with P(1), the event that Urn 1 is chosen. On these trials, EU_a is replaced by EU_2, and EU_u is replaced by EU_1

Model 15: Expected Utility model with ambiguity altering the weighting of the subjective risk parameter

$$P(U) = \frac{1}{1 + \exp\left(-\left(\beta 1 * (EU_a - EU_u)\right)\right)}$$

$$EU_a = (M_a^{\lambda ambig}) * P_a$$
$$EU_u = (M_u^{\lambda unambig}) * P_u$$

This model includes 3 parameters: $\beta_1, \lambda_{ambig}, \lambda_{unambig}$

This is an alternative formulation of the effect of categorical ambiguity upon the trade-off of outcome probability and outcome magnitude to that used in model 14. As for model 14, we include this for comparability with other work in the literature that has explored decision-making under ambiguity in healthy adults[10.]

On each ambiguous trial, P(U) is the event that the unambiguous urn is chosen. On unambiguous trials, P(U) is replaced with P(1), the event that Urn 1 is chosen. On these trials, EU_a is replaced by EU_2, and EU_u is replaced by EU_1. The categorical presence or absence of ambiguity is allowed to alter the relative weighting of probability and magnitude information. In practice, this model is identical to model 2 (see Methods) except that separate λ parameters are used for ambiguous urns (λ_{ambig} is used for ambiguous urns, urn A) and unambiguous urns ($\lambda_{unambig}$ is used for unambiguous urns, urn U, on ambiguous trials and for both Urn 1 and Urn 2.)

<u>Model 16. EU model with missing information informing second order probabilities (SOP).</u>

$$P(U) = \frac{1}{1 + \exp\left(-\left(\beta 1 * (EU_a - EU_u)\right)\right)}$$

$$P(1) = \frac{1}{1 + \exp\left(-\left(\beta 1 * (EU_2 - EU_1)\right)\right)}$$

$EU = M^{\lambda} * P$

$$P_a = \int \phi(p; c)\, Beta(p;\ k + 1, n - k + 1)\, dp$$

$$\phi(p; c) = \frac{1 - exp(-p * c)}{1 - exp(c)}$$

This model includes 3 parameters: β_1, λ, c

This model also belongs to the EU class and is included for comparability with other work in the literature that has explored decision-making under ambiguity in healthy adults[10]. On each ambiguous trial, P(U) is the event that the unambiguous urn is chosen. On unambiguous trials, P(U) is replaced with P(1), the event that Urn 1 is chosen. On these trials, EU_a is replaced by EU_2, and EU_u is replaced by EU_1. M_a, M_u, M_1 and M_2 are as defined in Models 1-4. For unambiguous urns, the probability of drawing an 'O' (P_u, P_1 and P_2) is simply calculated by k/n where k is the number of 'O's and n = 50 (the total number of tokens revealed). Hence EU_u, EU_1 and EU_2 are as defined in model 2. For ambiguous urns, the first order probability (p) that an 'O' will be drawn from the urn is estimated using ϕ(p;c)=(1-exp(-p*c))/(1-exp(c)) where c allows for non-linear effects of extreme probabilities. The second-order distribution of these probabilities is given by $\mathrm{Beta}(p;\ k + 1, n - k + 1)$. P_a is then calculated as the integral of the first and second order probabilities, i.e. $P_a = \int \phi(p;c)\ \mathrm{Beta}(p;\ k+1,n-k+1)\ dp$. This is effectively an alternate formulation of the rationale influence of missing information on estimation of P_a (versus using

the beta-binomial correction adopted in models 1-4) that also allows for non-linear effects of extreme probabilities.

Section 5 Exploring Biased Priors: models 17-21.

One possible explanation for increased avoidance of the ambiguous urn as a function of missing information might be that participants had a prior bias to treat obscured tokens as if they were 'O's, i.e. a pessimistic prior that fills in missing information by assuming a negative scenario. Conversely, some participants may have optimistic priors, where they are more likely to assume obscured tokens are 'harmless 'X's'. To explore this possibility, we fitted five additional models with fixed or variable pessimistic priors. In each of these models we sought to determine if a pessimism prior alone could account for irrational avoidance of the ambiguous urns, hence we did not include either $\beta_0 * C$ or $\beta_3 * A$ in these models.

Model 17: Assuming application of a pessimistic prior when information is missing.

$$P(U) = \frac{1}{1 + \exp\left(-(\beta 1 * Mdiff + \beta 2 * |\log|Pdiff)\right)}$$

This model includes 2 parameters: β_1, β_2.

P_a is defined as $P_a = \frac{k+50-n}{50}$; P_u, P_1, P_2 = k/50 where k is the number of 'O's in the tokens revealed and n is the number of tokens revealed.

On each ambiguous trial, P(U) is the event that the unambiguous urn is chosen. On unambiguous trials, P(U) is replaced with P(1), the event that Urn 1 is chosen; urns are

randomly allocated to be Urn 1 or Urn 2 as described for models 1-4. In order to investigate whether choice behavior when confronted with missing information can be explained by use of a pessimistic prior, we fit a model where all missing tokens are assumed to be 'O's. The probability of drawing an 'O' from the ambiguous urn is now calculated as $P_a = \frac{k+50-n}{50}$ where k is the number of 'O's in the tokens revealed and n is the number of tokens revealed. P_u, P_1 and P_2 are calculated as k/50 as before. *Pdiff* and *Mdiff* are otherwise as defined in model 3.

Model 18: Allowing individuals to vary in their degrees of optimism or pessimism

$$P(U) = \frac{1}{1 + \exp\left(-(\beta 1 * Mdiff + \beta 2 * |\log|Pdiff)\right)}$$

For unambiguous urns: P_u = k/n where k is the number of 'O's in the tokens revealed, and n is the number of tokens revealed.

For ambiguous urns: $P_{bayes} = \int p * Beta(p;\ k+1, n-k+1)\ dp$

i.e. P_{bayes} is the unbiased beta-binomial corrected estimate of the proportion of 'O's used as P_a in models 1-4.

Pessimism/optimism biases are then incorporated as follows:

If $\alpha < 1$ $P_a = \alpha P_{bayes} + (1-\alpha)P_{pess}$ where $P_{pess} = \frac{k+50-n}{50}$

If $\alpha > 1$ $P_a = (2-\alpha)P_{bayes} + \left(1-(2-\alpha)\right) * P_{opt}$ where $P_{opt} = \frac{k}{50}$

This model includes 3 parameters: β_1, β_2, α

On each ambiguous trial, P(U) is the event that the unambiguous urn is chosen. On unambiguous trials, P(U) is replaced with P(1), the event that Urn 1 is chosen. On unambiguous trials, P_1 and P_2 are given by k/n as for P_u (as in model 3). *Pdiff* and *Mdiff* are calculated as in model 3. Missing information is allowed to influence calculations of P_a as follows. A flexible pessimism/optimism bias is modelled using a participant-specific constant α which allows participants to be biased away from the beta-binomial corrected estimate of P_a used in models 1-4 (here termed P_{bayes}) towards a 'fully' pessimistic or optimistic prior (i.e. towards the assumption that all obscured tokens are either 'O's or 'X's). The direction and extent of this bias (as estimated by α) can vary across participants. We note that that modelling a flexible pessimism/ optimism prior in this way improved model fit versus use of a model with a fully pessimistic prior (i.e. model 17) but did not explain participant behavior as well as model 3, suggesting that participants' behavior when faced with missing information cannot be fully captured by this combined use of pessimistic/ optimistic priors together with a rational beta-binomial adjustment of the probability of drawing an 'O' as a function of missing information.

Model 19: Expected Utility model with pessimistic prior

$$P(U) = \frac{1}{1 + \exp\left(-\left(\beta 1 * (EU_a - EU_u)\right)\right)}$$

$$EU = (M^{\lambda}) * P$$

$P_a = \frac{k+50-n}{50}$; P_u, P_1, P_2 = k/n

where k is the number of 'O's in the tokens revealed, and n is the number of tokens revealed

This model includes 2 parameters: β_1, λ

This model takes the baseline EU model (model 2) and uses the pessimistic prior formulation from Model 17 to calculate P_a. All missing tokens are assumed to be 'O's: $P_a = \frac{k+50-n}{50}$. On each ambiguous trial, P(U) is the event that the unambiguous urn is chosen. On unambiguous trials, P(U) is replaced with P(1), the event that Urn 1 is chosen, EU_u is replaced by EU_1 and EU_a is replaced by EU_2. The expected utility (EU) is calculated for each urn, on each trial, as follows: EU = $M^{\lambda} * P$. Both λ and β_1 are estimated across both ambiguous and unambiguous trials. As in the other EU models, expected utilities are not z-scored.

<u>Model 20: Expected Utility model allowing individuals to vary in their degrees of either optimism or pessimism</u>

$$P(U) = \frac{1}{1 + \exp\left(-\left(\beta 1 * (EU_a - EU_u)\right)\right)}$$

$$EU = (M^{\lambda}) * P$$

For unambiguous urns: P = k/n where k is the number of 'O's in the tokens revealed, and n is the number of tokens revealed.

For ambiguous urns: $P_{bayes} = \int p * Beta(p;\ k+1, n-k+1)\ dp$

i.e. P_{bayes} is the unbiased beta-binomial corrected estimate of the proportion of 'O's used as P_a in models 1-4.

Pessimism/optimism biases are then incorporated as follows:

If $\alpha < 1$ $P_a = \alpha P_{bayes} + (1-\alpha)P_{pess}$ where $P_{pess} = \frac{k+50-n}{50}$

If $\alpha > 1$ $P_a = (2-\alpha)P_{bayes} + \left(1-(2-\alpha)\right) * P_{opt}$ where $P_{opt} = \frac{k}{50}$

This model includes 3 parameters: β_1, λ, α

This model takes the baseline EU model (model 2) and introduces a flexible pessimism/optimism bias as used in model 18. As in model 18, a participant-specific constant α allows participants to be biased away from the beta-binomial corrected estimate of P_a used in models 1-4 (here termed P_{bayes}) towards a 'fully' pessimistic or optimistic prior (i.e. towards the assumption that all obscured tokens are either 'O's or 'X's). The direction and extent of this bias (as estimated by α) can vary across participants.

As in model 2, on each ambiguous trial, P(U) is the event that the unambiguous urn is chosen. On unambiguous trials, P(U) is replaced with P(1), the event that Urn 1 is chosen, EU_u is replaced by EU_1 and EU_a is replaced by EU_2. The expected utility (EU) is calculated for each urn, on each trial, as follows: $EU = M^{\lambda} * P$. Both λ and β_1 are estimated across both ambiguous and unambiguous trials. Expected utilities are not z-scored. All variables not specified here are as defined in model 2.

Model 21: EU model with missing information informing second order probabilities (SOP) and a flexible pessimism/optimism bias

$$P(U) = \frac{1}{1 + \exp\left(-\left(\beta 1 * (EU_a - EU_u)\right)\right)}$$

$$EU = (M^{\lambda}) * P$$

For unambiguous urns: P = k/n where k is the number of 'O's in the tokens revealed, and n is the number of tokens revealed.

For ambiguous urns: $P_{SOP} = \int \phi(p;c)\, Beta(p;\ k+1, n-k+1)\, dp$

$$\phi(p;c) = \frac{1 - exp(-p * c)}{1 - exp(c)}$$

$$If\ \alpha < 1 \qquad P_a = \alpha P_{SOP} + (1-\alpha)P_{pess} \text{ where } P_{pess} = \frac{k+50-n}{50}$$

$$If\ \alpha > 1 \qquad P_a = (2-\alpha)P_{SOP} + \big(1-(2-\alpha)\big) * P_{opt} \text{ where } P_{opt} = \frac{k}{50}$$

This model includes 4 parameters: $\beta_1, \lambda, \alpha, c$

This model is also included for comparability with other work in the literature that has explored decision-making under ambiguity in healthy adults[10]. It effectively combines model 16 and model 20. On each ambiguous trial, P(U) is the event that the unambiguous urn is chosen. On unambiguous trials, P(U) is replaced with P(1), the event that Urn 1 is chosen. On these trials, EU_a is replaced by EU_2, and EU_u is replaced by EU_1. M_a, M_u, M_1 and M_2 are as defined in Models 1-4. For unambiguous urns, the probability of drawing an O (P_u, P_1 and P_2) is simply calculated by k/n where k is the number of 'O's and n = 50 (the total number of tokens revealed). Hence EU_u, EU_1 and EU_2 are as defined in model 2. For ambiguous urns, the first order probability (p) that an O will be drawn from the urn is estimated using ϕ(p;c)=(1-exp(-p*c))/(1-exp(c)) where c allows for non-linear effects of extreme probabilities. The second-order distribution of these probabilities is given by $\mathrm{Beta}(p;\ k+1, n-k+1)$. P_{SOP} is then calculated as the integral of the first and second order probabilities, i.e. P_a=∫ϕ(p;c) Beta(p; k+1,n-k+1) dp (as in model 16). Finally, a participant-specific constant α allows participants to be biased away from the P_{SOP} estimate towards a 'fully' pessimistic or optimistic prior (i.e. towards the assumption that all obscured tokens are either 'O's or 'X's). The direction and extent of this bias (as estimated by α) can vary across participants (this bias is implemented in the same manner as for models 18 and 20).
