# Supplementary material for: The Computational and Neural Substrates of Ambiguity Avoidance in Anxiety
Source: Comput Psychiatr. 2022 Feb 3;6(1):8–33. doi: 10.5334/cpsy.67 (PMC9223033; doi:10.5334/cpsy.67)
Supplement: Supplementary Analyses: Response Time Analyses. [file cpsy-6-1-67-s4.pdf]

Response Time Analyses

On each trial in our task, two urns were presented, and participants decided which urn to choose. To avoid contamination of BOLD activity related to urn comparison by activity related to participant's motor response, urns were presented for 2.5 – 5.5 seconds prior to the question mark indicating that participants had up to 5 seconds to enter their choice. We prioritized the ability to separate out BOLD activity linked to different trial stages over the ability to conduct response time (RT) analyses, as our key behavioral measure was urn choice. Nonetheless, RT analyses might enable us to address, in part at least, 'difficulty' related accounts of our findings. A common metric used as a proxy for trial difficulty is absolute expected value difference, $|EV_2-EV_1|$, where EV = Outcome Probability x Outcome Magnitude. Larger expected value differences indicate easier trials, as the superior urn is more easily apparent. Choice behavior in our task is better explained by separate influences of differences in outcome magnitude and probability as opposed to a single parameter of difference in expected value. Nevertheless, EV difference remains the most succinct and applicable proxy for overall trial difficulty. (Note, in models 2 and 4, we allow the relative weighting of probability and magnitude to vary between individuals, and hence use the term Expected Utility as opposed to Expected Value, with $EU = M^{\lambda} * P$). We hence commenced by investigating whether EV difference on unambiguous trials influenced RT. We did not apply a log modulus transform to the proportion of 'O's, in line with conventional formations of expected value; however, we note that the results reported here hold if a log modulus transform is applied. At a group level, expected value difference ($|EV_2-EV_1|$) on unambiguous trials had a significant effect on response time, as measured from urn presentation, with larger expected value differences being associated with shorter RTs, $t(30)=-2.9$, $p=0.0066$; sign-rank, $z=-2.3$, $p=0.023$, two-tailed (the betas for the effect of expected value difference on

RTs were not normally distributed across participants). The effect of $|EV_2\text{-}EV_1|$ on unambiguous trial response time did not correlate significantly with trait anxiety, p>0.6, Spearman two-tailed.

The above analysis was a basic sanity check to see if we obtain previously reported effects of EV difference on choice response times in our paradigm despite the jittered interval prior to the response window. We next examined response times as a function of trial type (AT,UT) and urn choice (AC, UC) and whether these varied as a function of anxiety. These distributions did not violate assumptions of normality. Our findings revealed, that at a group level, ambiguous trials had longer RTs than unambiguous trials, t(30)=4.7, p=5.1e-5, two-tailed (see **Table S3**). The relationship between the difference in RTs for ambiguous versus unambiguous trials and trait anxiety did not reach significance; r(29) = 0.27, p =0.15, Pearson two-tailed.

On ambiguous trials, across all levels of missing information, there was no significant difference between mean reaction times when participants chose the ambiguous urn versus the unambiguous urn

	**All Subjects**		**Low Anxiety**		**High Anxiety**	
	**Mean (ms)**	**Standard Error (ms)**	**Mean (ms)**	**Standard Error (ms)**	**Mean (ms)**	**Standard Error (ms)**
**Ambiguous Trials**	5049.3	52.9	5022.6	53.1	5074.4	91.1
**Unambiguous Trials**	4951.7	51.4	4964.5	66.0	4939.8	80.1
**Ambiguous Chosen Trials**	5006.7	62.8	4965.0	62.4	5045.8	108.1
**Unambiguous Chosen Trials**	5068.1	50.8	5049.8	53.0	5085.3	86.6

Supplementary Table 3: Response times for urn choice. Means and standard errors (ms) are presented for response time from urn presentation to choice by trial type: ambiguous trials, unambiguous trials, ambiguous chosen trials and unambiguous chosen trials. These values are shown for the whole participant sample and for participant subgroups created by a median split on STAI trait anxiety scores (Low anxiety = below median split; High anxiety = above median split).

(AC – UC), $t(30) = -1.7$, $p = 0.11$, two-tailed. There was also no significant correlation between the time taken to choose the ambiguous urn versus the unambiguous urn (AC – UC) and trait anxiety, $r(29) = 0.18$, $p = 0.33$, Pearson two-tailed.

Turning to the effect of missing information level; at a group level there was a significant effect of missing information level on response time, $t(30)=-2.7$, $p=0.012$, two-tailed; RTs decreased as missing information level increased. This finding could be accounted for by participants increasingly adopting a simple ambiguity avoidance heuristic that does not entail rationale evaluation of outcome probability and magnitude at high missing information levels. Trait anxiety showed no significant relationship with the change in RTs as a function of missing information level ($p = 0.19$, Pearson two-tailed).

At a group-level, the effect of missing information on RTs was particularly apparent on trials where participants avoided the ambiguous option (unambiguous chosen trials). On these trials there was a significant influence of missing information on RTs, $t(30)=-2.976$, $p=0.0057$, two-tailed (i.e. participants were faster at choosing the unambiguous urn as level of missing information increased). On trials where participants chose the ambiguous option, there was no significant influence of missing information on RTs, $t(30)=-0.037$, $p=0.97$, two-tailed. These findings are also consistent with ambiguity avoidance involving greater reliance on heuristics than choice of the ambiguous urn. Of note, when trials were broken down in this fashion, there was still no significant modulation by trait anxiety of the effect of missing information on RTs (Ambiguous Chosen (AC) trials: $p>0.6$, Pearson two-tailed; Unambiguous Chosen (UC) trials: $p>0.3$, Pearson two-tailed.

These supplementary RT results suggest that differences in trial difficulty are unlikely to underlie the relationship between high trait anxiety and elevated frontal activation as a function of missing information on ambiguous chosen (AC) trials, or across ambiguous trials in general. First, group-level RT effects as a function of missing information were only seen on UC trials and not AC trials. Second, there was no relationship between trait anxiety and the effect of missing information upon RTs for either AC or UC trials.

Finally, given absolute expected value difference can be used as a proxy of trial difficulty, we also examined how this varied as a function of missing information and urn choice on ambiguous trials. This revealed that, on trials where the ambiguous urn was chosen (AC), absolute expected value difference increased with missing information level, with this showing a trend-level tendency to be more pronounced in high trait anxious participants (**Figure S15**). Given frontal activation linked to task difficulty would be expected to decrease with absolute expected value difference, this finding is also inconsistent with a task difficulty explanation of the relationship between trait anxiety and increased DACC and IFS activation as a function of missing information level on ambiguous chosen trials.

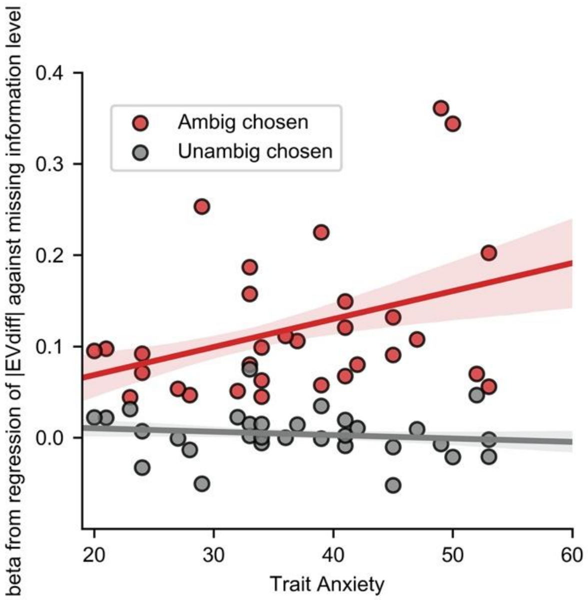

Supplementary Figure 15. The extent to which absolute expected value difference varies with missing information level as a function of urn choice is plotted against trait anxiety. For each participant, we z-scored the absolute expected value difference between urns (|EVdiff|) across trials and missing information level (A) across trials. We then divided trials as a function of the urn chosen (ambiguous or unambiguous) and regressed absolute expected value difference on missing information level. Here we plot the betas from these regressions against participant trait anxiety (as indexed by STAI trait scores). Betas for trials where the ambiguous urn was chosen (AC) are shown in red and betas for trials where the unambiguous urn was chosen (UC) are shown in grey. On AC trials, all participants showed positive betas for the regression of absolute expected value difference on missing information level, group-level signed rank test of beta values against zero: $z = 4.86$, $p = 1.17e\text{-}06$; the magnitude of these betas showed a trend to increase as a function of trait anxiety, $\rho(29) = 0.32$, $p=0.077$, Spearman, 2-tailed. On UC trials, for participants as a group, there was no significant relationship between absolute expected value difference and level of missing information, $z = 1.04$, $p = 0.30$; here there was a non-significant decrease in betas for the regression of absolute expected value difference on missing information level as a function of trait anxiety, $\rho(29) = -0.26$, $p = 0.16$, Spearman two-tailed.
