# Supplementary material for: The Computational and Neural Substrates of Ambiguity Avoidance in Anxiety
Source: Comput Psychiatr. 2022 Feb 3;6(1):8–33. doi: 10.5334/cpsy.67 (PMC9223033; doi:10.5334/cpsy.67)
Supplement: Supplementary Analyses: fMRI analyses. — This comprises analyses of the relationship between ILDAA scores and ROI activity, alternate ROI definition and results, results from a whole brain analysis and outcome time analyses. [file cpsy-6-1-67-s5.pdf]

Supplementary fMRI analyses.

ILDAA correlations with ROI activity to level of missing information.

Given the positive relationship between trait anxiety and both information-level dependent ambiguity aversion and increased dACC and IFS activity to missing information, we directly examined the relationship between ILDAA scores and activity in our regions of interest as a function of missing information level. Higher ILDAA scores were associated with increased dACC activity as a function of level of missing information; rho(29) = 0.488, p= 0.0059, pcorr = 0.030, Spearman, two-tailed. This relationship did not vary significantly as a function of urn choice, pcorr>.1. ILDAA scores were also associated with increased activity in left IFS to level of missing information on ambiguous trials where participants chose the ambiguous urn but this did not survive correction for multiple comparisons (rho(29) = 0.404, p= 0.0248, pcorr = 0.12, Spearman, two-tailed), nor was this relationship significantly different to that with activity on trials where participants chose the unambiguous urn. There were no other significant associations between ILDAA scores and ROI activity to level of missing information.

Alternate ROI definition and results.

Two main strategies for functional definition of regions of interest (ROIs) can be identified within the fMRI literature. The first, as adopted in the analyses reported in the main manuscript, uses activation co-ordinates reported by prior studies of related cognitive processes. The second uses orthogonal contrasts from the current dataset to define the regions of interest. The latter strategy has the advantage that the ROIs are defined using data from the same

participants. A potential disadvantage is that if individuals low or high in anxiety contribute more to the cross-group activation maps, activation in these individuals might be differentially represented in the ROI definition. In this section, we conduct additional analyses using ROIs defined by this second approach to increase confidence that our choice of a-priori ROIs did not selectively influence the reported results.

We were primarily interested in activation to level of missing information across all ambiguous trials or with these trials broken down according to the urn chosen; additionally, we were especially interested in how ROI activity, for these contrasts, was modulated by trait anxiety. To define ROIs for these analyses we used a group-level contrast of all ambiguous trials versus all unambiguous trials (this contrast is orthogonal to contrasts examining changes in missing information within ambiguous trials and does not selectively include or exclude participants as a function of anxiety levels). This was conducted using FSL's randomise tool with 5000 permutations, and a minimum cluster forming threshold of $z = 3.1$ and a cluster p threshold of 0.05. We masked the resulting t-stat map with the thresholded z-stat map, to leave only clusters that survived whole-brain cluster-based correction for multiple comparisons. This revealed clusters that spanned the dorsal anterior cingulate cortex and left and right inferior frontal sulcus (see **Whole brain results section** below**)**. No clusters were evident at this threshold in right or left rostrolateral prefrontal cortex. To obtain dACC and IFS ROIs of equivalent volume to those used in the main analyses, we increased the minimum t-stat until the new ROIs reduced to a comparable volume: t=4 for dACC, t=5.5. for left IFS and t=4.1 for right IFS. These ROIs, as shown in **Figure S16**, were used for the analyses reported below.

Group-level results

Within these new ROIs for dACC, right IFS and left IFS, we examined activity yoked to urn presentation on ambiguous trials as a function of level of missing information (*A*). All statistics reported here are multiple comparison corrected for the 3 ROIs investigated. Activity in all three ROIs increased linearly as a function of level of missing information: dACC: $t(30) = 2.9$, $p = 0.0063$, $p_{corr} = 0.019$; left IFS: $t(30) = 3.4$, $p = 0.0022$, $p_{corr} = 0.0066$; right IFS: $t(30) = 4.1$, $p = 0.00029$, $p_{corr} = 0.00088$, two-tailed.

We next examined activation to missing information, on ambiguous trials, as a function of whether participants went on to choose the unambiguous urn (unambiguous chosen, UC) or the ambiguous urn (ambiguous chosen, AC), see **Methods**.

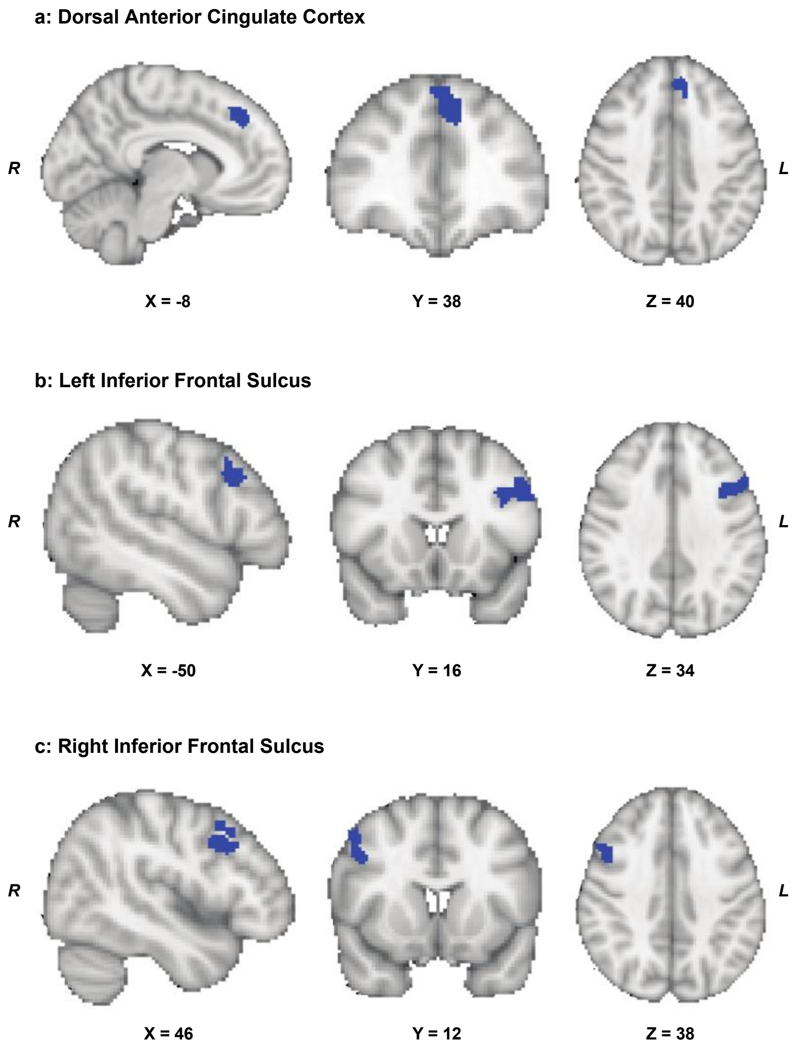

Supplementary Figure 16: Alternate regions of interest were created using group-level contrasts orthogonal to those of interest. We used the group-level contrast for ambiguous trials versus unambiguous trials to create regions of interest within which to investigate activation as a function of missing information (conducted within ambiguous trials alone). Here we present sagittal, coronal and axial views of (a) the dorsal anterior cingulate cortex (dACC) ROI; (b) the left inferior frontal sulcus (lIFS) ROI and (c) the right inferior frontal sulcus (rIFS) ROI. Radiological convention is used (R indicates right hemisphere).

Across participants, dACC and IFS activation yoked to urn presentation increased as a function of missing information level on trials where participants subsequently chose the ambiguous urn; dACC: $t(30) = 3.5$, $p = 0.0016$, $p_{corr} = 0.0048$; left IFS: $t(30) = 4.0$, $p = 0.00039$, $p_{corr} = 0.0012$; right IFS: $t(30) = 5.9$, $p = 1.8e\text{-}6$, $p_{corr} = 5.3e\text{-}6$, two-tailed. As previously observed using the ROIs defined by coordinates taken from the literature (see main manuscript), the response in dACC and IFS to missing information level was weaker on trials where participants chose the unambiguous urn; dACC: $t(30) = 0.95$, $p = 0.35$ uncorrected; left IFS: $t(30) = 2.0$, $p = 0.054$, $p_{corr} = 0.16$, right IFS: $t(30) = 2.7$, $p = 0.013$, $p_{corr} = 0.038$, two-tailed); however we note that the right IFS response to missing information level on unambiguous chosen trials now reaches significance. The difference in response to missing information by trial type gave the following results: dACC, $t(30) = 2.1$, $p = 0.044$; left IFS: $t(30) = 1.1$, $p = 0.26$, right IFS: $t(30) = 1.8$, $p = 0.083$, two-tailed.

Effects of trait anxiety upon the prefrontal cortical response to missing information.

We next investigated the influence of trait anxiety on the response to missing information in the newly defined dACC and IFS ROIs. The group-level analyses reported above indicated that the response to missing information in both dACC and IFS was somewhat stronger on trials where participants went on to select the ambiguous urn. Replicating the ROI results reported in the main text, correlational analyses revealed that this pattern was amplified in high trait anxious individuals. Specifically, on trials where participants went on to choose the ambiguous urn (AC), trait anxiety was significantly positively correlated with the response to level of missing information in both dACC: $rho(29) = 0.43$, $p = 0.015$, $p_{corr} = 0.045$, Spearman two-tailed and left IFS: $rho(29) = 0.56$, $p = 0.0010$, $p_{corr} = 0.0031$, Spearman two-tailed. The effect was in the same direction in right IFS but did not reach significance, $rho(29) = 0.34$, $p = 0.059$, $p_{corr} = 0.18$,

Spearman two-tailed. No equivalent relationship was observed in either dACC or IFS on trials where the unambiguous urn was selected; dACC: rho(29)= -0.092, p>0.1; left IFS: rho(29)= -0.12, p>0.1; right IFS: rho(29) = 0.034, p>0.1, all Spearman, two-tailed. The difference in activation to missing information as a function of whether the ambiguous or the unambiguous urn was selected showed a positive correlation with trait anxiety in both dACC and left IFS: dACC, rho(29) = 0.40, p = 0.028; new left IFS: rho(29) =0.40, p = 0.026 , Spearman, two-tailed. As reported for the ROIs used in the main analyses, there were no significant effects of trait anxiety upon outcome-yoked ROI activation in these alternately defined ROIs.

Whole Brain Results

For completeness, we provide additional results from the whole brain analysis used to functionally define dACC and IFS ROIs. The results shown in Figures S17 to S19 and reported in Tables S4 -S7 are whole brain corrected using FSL's randomise tool with 5000 permutations, a minimum cluster forming

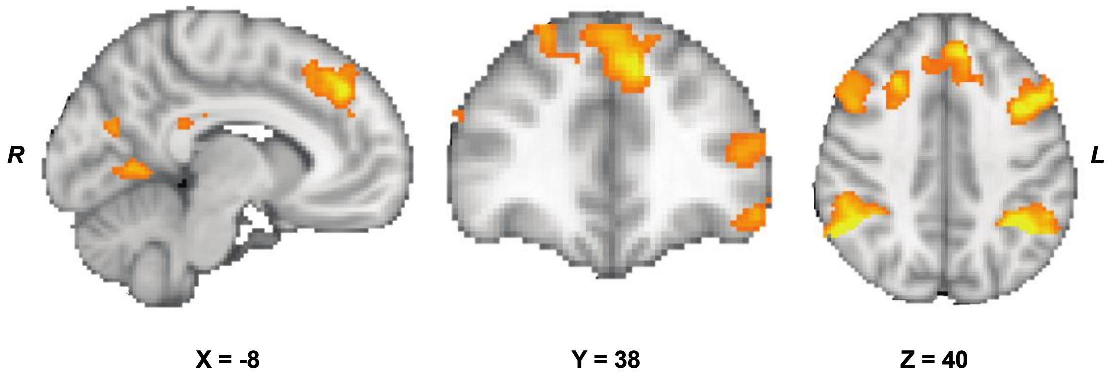

threshold of z = 3.1 and a cluster p threshold of 0.05.

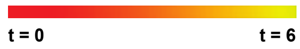

Supplementary Figure 17: BOLD activity to the categorical presence versus absence of ambiguity.

Here we show sagittal, coronal and axial views of the group-level contrast for blood oxygen level dependent (BOLD) activity on ambiguous versus unambiguous trials at urn presentation time. We display the T-stat activation map for clusters that survive whole brain correction using a cluster forming threshold of z = 3.1 and a cluster p threshold of 0.05. The cluster-correction analysis was conducted using randomise (FMRI Expert Analysis Tool, v6.00) with 5000 permutations. The group-level t-stat map is masked with the z-stat whole brain corrected map to leave only whole brain corrected voxels. Radiological convention is used (R indicates right hemisphere). Coordinates are given in MNI space; activation clusters are displayed on the MNI_152_T1_2mm brain.

Region (Harvard - Oxford atlas) First region listed = peak voxel. Other regions spanned by the cluster are also listed	Number of Voxels	Max t-stat (df = 30)	Peak voxel (X, Y, Z)	Center of Gravity (X, Y, Z)
Left middle frontal gyrus; left and right inferior frontal gyrus, left and right superior frontal gyrus, paracingulate gyrus extending into cingulate (anterior division), right middle frontal gyrus, left frontal pole	7497	7.11	-50,16, 34	-13, 25, 34
Right temporal occipital fusiform Cortex; right lingual gyrus, left supramarginal gyrus, left and right precuneus cortex	3224	9.27	30, -42, -14	-5, -57, 13
Left temporal occipital fusiform Cortex; left occipital fusiform gyrus, left middle temporal gyrus, left inferior temporal gyrus	1879	7.48	-30, -54, -8	-48, -47, -11
Right angular gyrus; right lateral occipital cortex, superior division, right supramarginal gyrus, posterior division	1395	7.67	48, -52, 40	49, -55, 32
Right middle temporal gyrus, temporooccipital part; right inferior temporal gyrus, temporooccipital part	1177	6.15	58, -52, -10	59, -47, -11
Right cerebellum (from cerebellar atlas: right crus II)	672	6.53	40, -72, -46	34, -71, -46
Right posterior cingulate gyrus (cluster extends bilaterally)	468	5.45	6, -28, 28	1, -29, 28

Supplementary Table 4: Activation clusters for the categorical presence versus absence of ambiguity (ambiguous trials versus unambiguous trials). Here we report significant BOLD activation clusters for the group level contrast of Ambiguous Trials versus Unambiguous Trials. Clusters reported survived whole brain correction using a cluster forming threshold of z = 3.1 and a cluster p threshold of 0.05 (conducted using randomise from FMRI Expert Analysis Tool, v6.00 with 5000 permutations). For each cluster, we report the region in the Harvard-Oxford atlas corresponding to the voxel in the cluster with the highest t-stat, followed by other regions spanned by the same cluster. We also report the following for each cluster: Number of Voxels (the number of voxels in the cluster), Max t-stat (the maximum t-statistic for any voxel in the cluster), Peak voxel (the X,Y,Z coordinates of the voxel with the maximum t-statistic in MNI space), Center of Gravity (the X,Y,Z coordinates of the center of gravity in MNI space).

Region (Harvard - Oxford atlas) First region listed = peak voxel. Other regions spanned by the cluster are also listed	Number of Voxels	Max t-stat (df = 30)	Peak voxel (X, Y, Z)	Center of Gravity (X, Y, Z)
Left occipital pole; left occipital fusiform gyrus, left lingual gyrus, left lateral occipital cortex,	2713	9.37	-16, -98, 8	-19, -92, 0
Right occipital pole; right occipital fusiform gyrus, right lateral occipital cortex, right lingual gyrus	1939	8.5	18, -96, 8	21, -89, 1
Right anterior supramarginal gyrus; right posterior supramarginal gyrus, right parietal operculum cortex, right planum temporale	791	5.27	64, -32, 28	58, -34, 21
Right insular cortex; right central opercular cortex, right frontal operculum cortex	501	5.87	34, 16, 6	36, 13, 7

Supplementary Table 5: **Activation clusters for the categorical absence versus presence of ambiguity (unambiguous trials versus ambiguous trials).** Here we report significant BOLD activation clusters for the group level contrast of Ambiguous Trials versus Unambiguous Trials. This is the reverse of the contrast reported in Table S4. Clusters reported survived whole brain correction using a cluster forming threshold of z = 3.1 and a cluster p threshold of 0.05 (conducted using randomise from FMRI Expert Analysis Tool, v6.00 with 5000 permutations). For each cluster, we report the region in the Harvard-Oxford atlas corresponding to the voxel in the cluster with the highest t-stat, followed by other regions spanned by the same cluster. We also report the following for each cluster: Number of Voxels (the number of voxels in the cluster), Max t-stat (the maximum t-statistic for any voxel in the cluster), Peak voxel (the X,Y,Z coordinates of the voxel with the maximum t-statistic in MNI space), Center of Gravity (the X,Y,Z coordinates of the center of gravity in MNI space).

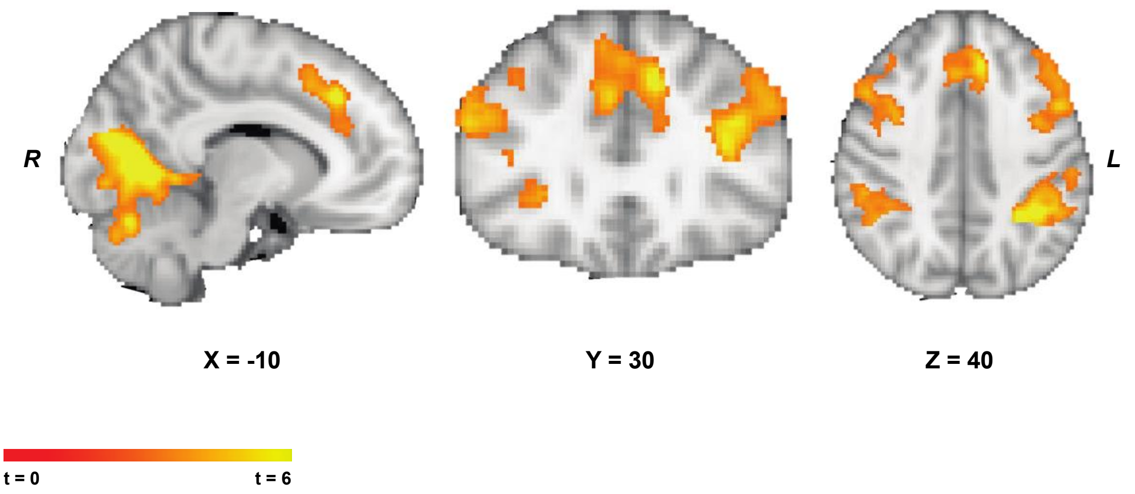

Supplementary Figure 18: Clusters where activation at urn presentation increases with missing information level on trials where participants subsequently chose the ambiguous urn. Here, we present sagittal, coronal and axial views of the cluster corrected t-stat map for activations that varied positively with missing information level at urn presentation time on trials where participants went on to choose the ambiguous urn. Clusters depicted survive whole brain correction using a cluster forming threshold of z = 3.1, and a cluster p threshold of 0.05. The cluster-correction analysis was conducted using randomise (FMRI Expert Analysis Tool, v6.00) with 5000 permutations. The group-level t-stat map is masked with the z-stat whole brain corrected map to leave only whole brain corrected voxels. Radiological convention is used (R indicates right hemisphere). Coordinates are given in MNI space; activation clusters are displayed on the MNI_152_T1_2mm brain.

Region (Harvard - Oxford atlas) First region listed = peak voxel. Other regions spanned by the cluster are also listed	Voxels	Max t-stat (df = 30)	Peak voxel (X, Y, Z)	Center of Gravity (X, Y, Z)
Lingual gyrus (primarily right V1); right temporal occipital fusiform cortex, left and right precuneus cortex, left and right intracalcarine cortex, left and right cuneal cortex, right inferior lateral occipital cortex, left and right cerebellum, right thalamus, left anterior and posterior supramarginal gyrus.	10339	9.83	6, -72, 4	3, -65, 5
Left middle frontal gyrus; left frontal pole, left inferior frontal gyrus, left precentral gyrus	2182	5.75	-34, 30, 18	-42, 23, 29
Left inferior temporal gyrus, temporooccipital part; left lateral occipital cortex, left temporal occipital fusiform cortex, left cerebellum	2138	6.36	-44, -50, -10	-43, -66, -4
Right frontal pole; right middle frontal gyrus, right Precentral gyrus, right inferior frontal gyrus, right insular cortex, right frontal orbital cortex	2043	6.57	52, 36, 24	42, 20, 30
Left paracingulate gyrus; right paracingulate gyrus, left and right anterior cingulate gyrus, left and right superior frontal gyrus	1273	6.35	-10, 30, 40	-1, 26, 42
Right posterior supramarginal gyrus; right superior parietal lobule, right angular gyrus	506	5.78	56, -40, 50	48, -41, 42

Supplementary Table 6: **Clusters where BOLD activity increases with levels of missing information, on trials where participants chose the Ambiguous Urn.** Here we report clusters where BOLD activation significantly increased with missing information level, on trials where participants went on to choose the ambiguous urn. Clusters reported survived whole brain correction using a cluster forming threshold of z = 3.1 and a cluster p threshold of 0.05 (conducted using randomise from FMRI Expert Analysis Tool, v6.00 with 5000 permutations). For each cluster, we report the region in the Harvard-Oxford atlas corresponding to the voxel in the cluster with the highest t-stat, followed by other regions spanned by the same cluster. We also report the following for each cluster: Number of Voxels (the number of voxels in the cluster), Max t-stat (the maximum t-statistic for any voxel in the cluster), Peak voxel (the X,Y,Z coordinates of the voxel with the maximum t-statistic in MNI space), Center of Gravity (the X,Y,Z coordinates of the center of gravity in MNI space). Note: the reverse contrast (regions where BOLD activity decreases with level of missing information on Ambiguous chosen trials) produced no significant clusters.

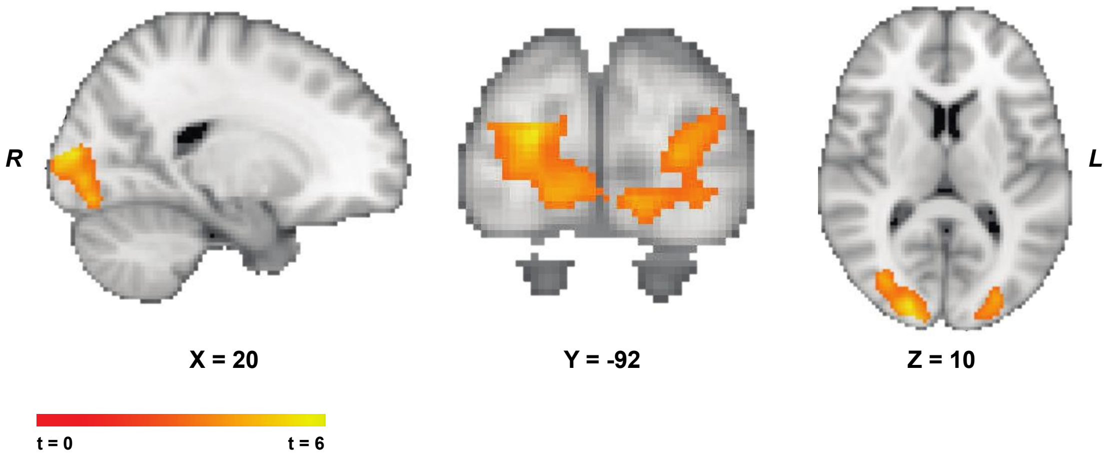

Supplementary Figure 19: Clusters where activation at urn presentation increases with information level (i.e. decreases with missing information level) on trials where participants subsequently chose the unambiguous urn. Here, we present sagittal, coronal and axial views of the cluster corrected t-stat map for activations that varied negatively with missing information level at urn presentation time on trials where participants went on to choose the unambiguous urn. Clusters depicted survive whole brain correction using a cluster forming threshold of z = 3.1, and a cluster p threshold of 0.05. The cluster-correction analysis was conducted using randomise (FMRI Expert Analysis Tool, v6.00) with 5000 permutations. The group-level t-stat map is masked with the z-stat whole brain corrected map to leave only whole brain corrected voxels. Radiological convention is used (R indicates right hemisphere). Coordinates are given in MNI space; activation clusters are displayed on the MNI_152_T1_2mm brain.

**Region (Harvard - Oxford atlas)**<br>**First region listed = peak voxel. Other regions spanned by the cluster are also listed**	**Voxels**	**Max t-stat (df = 30)**	**Peak voxel (X, Y, Z)**	**Center of Gravity (X, Y, Z)**
right occipital pole; left occipital pole, left and right lateral occipital cortex, left and right occipital fusiform gyrus, right lingual gyrus	2158	5.58	20, -92, 10	7, -89, 3

Supplementary Table 7. Clusters where BOLD activity decreases with level of missing information, on trials where participants chose the Unambiguous Urn. Here we report clusters where BOLD activation increased as missing information decreased on trials where participants went on to choose the unambiguous urn. Clusters reported survived whole brain correction using a cluster forming threshold of z = 3.1 and a cluster p threshold of 0.05 (conducted using randomise from FMRI Expert Analysis Tool, v6.00 with 5000 permutations). For each cluster, we report the region in the Harvard-Oxford atlas corresponding to the voxel in the cluster with the highest t-stat, followed by other regions spanned by the same cluster. We also report the following for each cluster: Number of Voxels (the number of voxels in the cluster), Max t-stat (the maximum t-statistic for any voxel in the cluster), Peak voxel (the X,Y,Z coordinates of the voxel with the maximum t-statistic in MNI space), Center of Gravity (the X,Y,Z coordinates of the center of gravity in MNI space). Note: the reverse contrast (regions where BOLD activity increases with level of missing information on Unambiguous chosen trials) produced no significant clusters.

Outcome Time Analyses

Following participant urn choice, a token was randomly selected by the computer from the chosen urn (with each of the 50 tokens in the urn being equally likely to be drawn) and displayed to participants, i.e. whether an ‘X’ or an ‘O’ had been drawn was revealed. While outcome yoked activation patterns were not a focus of our primary hypotheses, we report them here for completeness. We first examined activation patterns for our five primary regions of interest: dACC, left and right IFS and left and right RLPFC. Out of these regions, it was predominantly IFS that showed activation during the outcome period of the trial as detailed further below. We also conducted exploratory analyses of activity in the nucleus accumbens and amygdala given that these regions have been suggested to play a role in response to rewarding and aversive outcomes and implicated in the signalling of reward related and aversive prediction errors.

Group Level Results

<u>Primary ROIs: dACC, left and right IFS and left and right RLPFC.</u>

Activation at outcome time by trial type and urn chosen

Across participants, IFS showed significant activation at outcome time for both ambiguous and unambiguous trials, (ambiguous trials: right IFS: t(30)=4.10, p=0.00029, pcorr=0.0015, left IFS: t(30)= 3.39,p=0.0020, pcorr=0.0099; unambiguous trials: right IFS: t(30)=3.17 p=0.0034, pcorr=0.017, left IFS: t(30)= 2.28,p=0.030, pcorr=0.15. IFS activation was numerically weaker at outcome time on unambiguous trials, however the difference in activation

between unambiguous and ambiguous trials did not reach significance in either the left or right IFS ROI.

Breaking ambiguous trials down into those where participants had chosen the ambiguous urn and those where they had chosen the unambiguous urn revealed that right IFS activity at outcome was significantly stronger on ambiguous trials where participants had chosen the ambiguous option than on those where participants had chosen the unambiguous option ($t(30)= 3.77, p=0.00072$, pcorr=0.0036). Neither dACC nor RLPFC showed significant activation during the outcome period of ambiguous or unambiguous trials, nor did activity at outcome in these regions differ significantly as a function of whether participants had chosen the ambiguous or unambiguous urn.

Activation at outcome time as a function of level of missing information.

On ambiguous trials, activity in right IFS during the outcome period varied positively as a function of level of missing information (right IFS: $t(30) = 2.86$, $p = 0.0076$, pcorr = 0.038). No significant effects linked to this contrast were observed in any of the other ROIs.

We next broke ambiguous trials down according to the urn selected. On trials where participants chose the ambiguous urn, activity at outcome varied positively with level of missing information level in left and right IFS and dACC (left IFS: $t(30)= 4.05, p=0.00033$, pcorr=0.0017; right IFS: $t(30)=3.76$, $p=0.00073$, pcorr=0.0037; dACC: $t(30)=2.97$, $p=0.0058$, pcorr=0.029.) No equivalent pattern was seen on trials where participants chose the unambiguous urn ($ps>0.3$). The difference in effect of missing information as a function of urn selected was as follows: left IFS: $t(30)=2.61, p=0.014$, pcorr=0.0699; right IFS: $t(30)=2.71$, $p=0.011$, pcorr =0.055; dACC: $t(30) = 1.81$, $p=0.080$, pcorr=0.4.

Activity tracking outcome surprise

We characterised surprise as the -log ($P_{outcome\ token}$ (either X or O) drawn from the chosen urn) so that an outcome was more surprising the lower the probability that the computer would draw it given the tokens shown. Note, for trials where the ambiguous urn was selected, the beta-binomial corrected probability was used for $P_{outcome\ token}$ as for P_a . I.e., $P_{outcome\ token}$ was estimated by E(p), p~Beta(1+k, 1+n-k) where, here, k =number of the token that was drawn as the outcome by the computer shown in the urn and n = the total number of tokens revealed. We also characterised 'signed surprise' (i.e. the prediction error) as the signed version of surprise such that positive values corresponded to a better-than-expected outcome, and negative values to a worse-than-expected outcome.

At a group level, we did not observe significant activation in any of our primary ROIs to either surprise or signed surprise for either ambiguous trials or unambiguous trials. However, when ambiguous trials were broken down by urn chosen, we observed increased left and right IFS activity to surprise on trials where the ambiguous urn was chosen relative to trials where the unambiguous urn was chosen (surprise AC - UC: left IFS: t(30)= 3.32, p=0.0023, pcorr=0.012; right IFS: t(30)=3.03, p=0.0050, pcorr=0.025).

Activity at outcome time as a function of outcome received.

An 'O' represents the most negative outcome possible (potential receipt of shock) and an 'X' the most positive outcome possible on a given trial (no shock). As such, an 'X' outcome might be linked to the experience of relief. We next examined activation at outcome as a function of trial type and outcome received. Both dACC and right IFS showed an increase in

activity after receipt of a negative outcome on unambiguous trials, however this did not survive correction for multiple comparisons (dACC: t(30)= 2.58, p=0.015, pcorr=0.075; rIFS: t(30)=2.25, p=0.032, pcorr=0.16), nor did the trend for this pattern to be stronger on unambiguous than ambiguous trials (dACC: t(30)= 1.90, p=0.068 uncorrected, rIFS: t(30)=1.87, p=0.07 uncorrected). On ambiguous trials, response in our primary ROIs as a function of outcome received did not differ significantly between trials where the ambiguous urn was chosen and those where the unambiguous urn was chosen.

Trait Anxiety and activation within primary ROIs at Outcome Time

There was no significant correlation between trait anxiety and outcome time activity in dACC, left or right IFS or left or right RLPFC for any of the contrasts of interest described above.

<u>Additional ROIs for outcome analyses: bilateral Amygdala and Nucleus Accumbens.</u>

The Nucleus Accumbens (NAcc) and Amygdala have been implicated in both the response to outcome value and also in the response to outcome surprise, with the Nucleus Accumbens potentially primarily showing activation to positive prediction errors (i.e. positively signed surprise). We hence constructed additional regions of interest to examine activity in these regions following outcome receipt . Left and right ROIs for both structures were defined anatomically using the Harvard Oxford atlas. These analyses are exploratory as this was not our main focus of interest. Hence, we do not include these ROIs in our multiple comparisons

corrections and the results reported here need to be treated with caution and subject to replication.

Activation at outcome by trial type and urn chosen

Both right and left amygdala ROIs showed increased activity during the outcome period of both ambiguous and unambiguous trials (ambiguous trials: right amygdala: $t(30)=2.26$, $p=0.031$, left amygdala: $t(30)= 2.60$,$p=0.014$; unambiguous trials: right amygdala: $t(30)=3.48$ $p=0.0016$, left amygdala: $t(30)= 4.08$,$p=0.0003$. The right NAcc showed increased activity during the outcome period of ambiguous trials only (ambiguous trials: right NAcc: $t(30)=3.036$, $p=0.0049$.)

Activity tracking outcome surprise

Amygdala activity at outcome varied with how surprising the outcome was for unambiguous trials alone (unambiguous trials: right amygdala: $t(30)=2.286$ $p=0.0295$.) The extent to which amygdala activity tracked surprise on unambiguous trials varied positively with trait anxiety (right amygdala: $r(29)=0.398$, $p=0.026$, left amygdala: $r(29)=0.424$, $p=0.018$). This relationship reversed on ambiguous trials, with low anxious participants showing a stronger surprise signal in the amygdala (left amygdala: $r(29)= -0.411$, $p=0.022$). This led trait anxiety to also be significantly related to the difference in strength of surprise signal on ambiguous versus unambiguous trials (right amygdala: $r(29)=-0.385$, $p=0.032$, left amygdala: $r(29)=-0.44$, $p=0.013$). Activity in the nucleus accumbens did not significantly track outcome surprise at a

group level or as a function of trait anxiety. Neither the amygdala or nucleus accumbens showed a significant response to signed surprise, either at a group level or as a function of trait anxiety nor did the response to surprise or signed surprise in any of these ROIs vary significantly as a function of urn chosen.

Activity at outcome time as a function of outcome received.

The right nucleus accumbens showed an increase in activity after receipt of a positive (versus negative) outcome on ambiguous trials (NAcc: t(30)= 2.35, p=0.026), and a trend for greater activity after receipt of a positive (versus negative) outcome on ambiguous than unambiguous trials, t(30)= 1.76, p=0.089. This might conceivably reflect a 'relief' signal but we cannot distinguish this from simply a response to receiving the best available outcome. Activity in the amygdala did not vary as a function of outcome received by trial type. Across both regions, activity as a function of outcome received did not differ significantly between ambiguous trials where the ambiguous urn was chosen and those where the unambiguous urn was chosen.

Trait anxiety significantly modulated the response to outcome received across both left and right amygdala and nucleus accumbens regions of interest. In all four regions, elevated trait anxiety was associated with a stronger response to receipt of a positive (versus negative) outcome on ambiguous trials (right nucleus accumbens: r(29)=0.415, p=0.020, left nucleus accumbens: r(29)=0.402, p=0.025; right amygdala: r(29)=0.451, p=0.011, left amygdala: r(29)=0.39, p=0.028). No equivalent relationship between anxiety and activity to outcome received was observed on unambiguous trials in any of these four regions, ps>.1. This resulted in a positive relationship between trait anxiety and response to outcome received as a function of trial type (ambiguous versus unambiguous) which reached significance in the nucleus accumbens and showed a similar trend-level pattern in the amygdala: right nucleus accumbens: r(29)=0.406, p=0.024, left nucleus accumbens: r(29)=0.361, p=0.046; right amygdala: r(29)=0.345, p=0.058, left amygdala: r(29)=0.352, p=0.052). When ambiguous trials were broken down as a function of urn chosen, there was a trend level relationship between trait anxiety and activity to outcome received (positive versus negative) on urns where the ambiguous option was chosen in both the right nucleus accumbens and right amygdala (right NAcc: r(29)= .336,p=0.0642; right amygdala: r(29)= 0.342,p=0.0595). No other significant or trend level effects were observed.
